# Agnuside Stabilizes the Complex I Assembly Factor NDUFAF6 to Reinforce Mitochondrial Efficiency and Thermogenic Responsiveness

**DOI:** 10.1002/advs.202516501

**Published:** 2026-06-16

**Authors:** Qingwen Zhao, Li Xie, Qianzhuo Wang, Shuying Dai, Bei Li, Yingjuan Zhang, Zhe Sun, Yue Gao

**Affiliations:** ^1^ Zhejiang Key Laboratory of Traditional Chinese Medicine for the Prevention and Treatment of Senile Chronic Diseases, Affiliated Hangzhou First People's Hospital, School of Medicine Westlake University Hangzhou China; ^2^ College of Biotechnology and Bioengineering Zhejiang University of Technology Hangzhou China; ^3^ College of Mechanical Engineering Zhejiang University of Technology Hangzhou China

**Keywords:** agnuside, brown adipocytes, mitochondria, NDUFAF6, thermogenesis

## Abstract

Brown and beige adipocytes dissipate energy as heat, yet effective strategies to enhance their mitochondrial efficiency remain limited. Here, we identify Agnuside (AGN) as a selective stabilizer of the complex I assembly factor NDUFAF6. AGN directly binds cytosolic NDUFAF6, suppresses its ubiquitination, prolongs its half‐life, and facilitates mitochondrial import, thereby reinforcing complex I assembly and promoting coordinated stabilization of complexes III and IV within the respirasome, without altering complex II, complex V, or global mitochondrial biogenesis. Functionally, AGN exhibits a demand‐dependent metabolic profile. Under basal conditions, AGN enhances mitochondrial oxidative efficiency without activating overt UCP1‐dependent uncoupling. In contrast, cold exposure or chronic high‐fat feeding markedly potentiates its thermogenic impact, as evidenced by improved mitochondrial ultrastructure, increased UCP1 abundance, and elevated energy expenditure in brown adipose tissue, with similar mitochondrial reinforcement observed in inguinal white adipose tissue under sustained metabolic stress. Importantly, thermoneutral *Ucp1* knockdown does not abolish AGN‐mediated enhancement of respiratory complex assembly and ATP production, whereas genetic ablation of *Ndufaf6* eliminates these effects. Together, these findings establish AGN‐NDUFAF6 stabilization as a key regulatory mechanism governing adipose mitochondrial efficiency and thermogenic responsiveness, and highlight assembly‐factor targeting as a promising strategy to restore oxidative metabolism in metabolic dysfunction.

## 1 Introduction

Brown adipose tissue (BAT) is a primary site of non‐shivering thermogenesis, where mitochondria express uncoupling protein 1 (UCP1) to dissipate the proton gradient and generate heat rather than ATP [1]. Cold exposure or adrenergic stimulation markedly enhances BAT activity, promoting nutrient oxidation and oxygen consumption, and also induces the browning of inguinal white adipose tissue (iWAT), giving rise to beige adipocytes with thermogenic potential [2, 3]. During this process, the mitochondrial respiratory chain, composed of four multi‐heteromeric complexes (I‐IV), transfers electrons to oxygen while simultaneously pumping protons across the inner mitochondrial membrane [4, 5]. Thermogenic efficiency depends not only on mitochondrial abundance but also on the orderly assembly of these complexes and the structural stability of higher‐order supercomplexes, which enhance electron transfer efficiency, reduce leakage, and suppress excessive reactive oxygen species (ROS), thereby ensuring mitochondrial homeostasis and effective heat production [6].

Complex I (CI) assembly is coordinated by multiple factors, among which NDUFAF6 (C8orf38) has emerged as a pivotal non‐catalytic regulator [7, 8, 9]. Beyond its mitochondrial role, NDUFAF6 also acts in the cytosol, where it cooperates with HSP90 to stabilize nascent CI preproteins such as NDUFA10 and NDUFS3 prior to import. In the absence of NDUFAF6, these preproteins undergo ubiquitin‐proteasome degradation, leading to impaired CI assembly and elevated ROS [10]. Within mitochondria, NDUFAF6 associates with the ND1 module and NDUFS8 to promote early CI assembly [11]. This dual safeguard positions NDUFAF6 as a central hub for CI proteostasis and mitochondrial homeostasis; however, whether it functions as a rate‐limiting determinant of adipose thermogenesis, and whether its stabilization can be pharmacologically harnessed to enhance mitochondrial efficiency under metabolic stress, remains unresolved.

Natural small molecules with structural diversity and favorable safety profiles have emerged as attractive candidates to modulate adipose thermogenesis [12, 13]. Several plant‐derived compounds, including hyperforin from *Hypericum perforatum* and hydroxysafflor yellow A from safflower, have shown anti‐obesity effects in preclinical models [14, 15, 16]. Nevertheless, few natural products have been demonstrated to stably activate BAT or beige adipocytes, and most are limited by efficacy, selectivity, or safety concerns. Iridoid glycosides (IGs), a large class of monoterpenoid glycosides widely distributed in medicinal plants, are of particular interest for metabolic regulation [17]. Gentiopicroside and valepotriate suppress adipogenesis and counteract diet‐induced obesity, and agnuside prevents weight gain in ovariectomized rodents [18, 19, 20, 21]. Together, these results indicate that IGs influence metabolic homeostasis through diverse mechanisms, including inhibition of adipocyte differentiation, modulation of hormonal responses, and attenuation of diet‐induced fat accumulation. However, whether IGs can directly promote thermogenesis and enhance mitochondrial function in brown or beige adipocytes remains unknown, providing a critical rationale for our investigation.

Here, we identify agnuside (AGN), an IG isolated from *Vitex negundo*, as a natural small molecule that binds and stabilizes cytosolic NDUFAF6. This interaction suppresses NDUFAF6 ubiquitination, promotes its mitochondrial import, and enhances CI‐dependent supercomplex assembly. Stabilization of NDUFAF6 optimizes mitochondrial architecture, increases ATP production, and augments thermogenesis in a demand‐dependent manner, effects that are abolished by *Ndufaf6* depletion. Together, these findings establish NDUFAF6 as a central hub of adipose thermogenesis and highlight assembly factor pharmacology as a promising strategy for metabolic intervention.

## 2 Results

### 2.1 Agnuside Enhances UCP1 Protein Abundance and Mitochondrial Respiratory Capacity in Brown and Beige Adipocytes Independent of Differentiation

**FIGURE 1:**
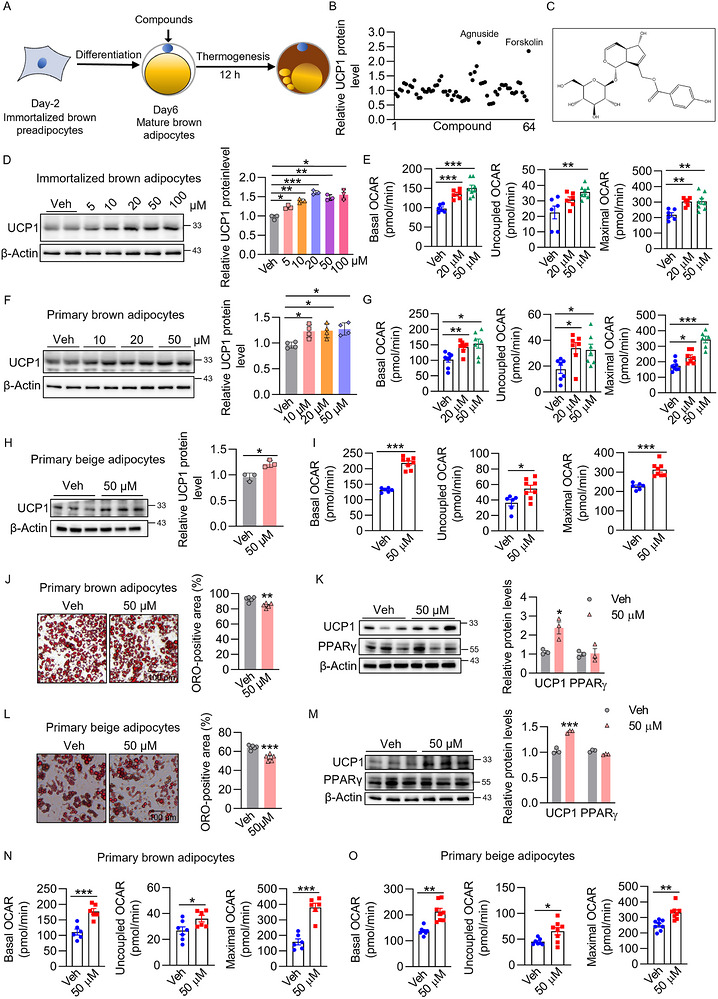
Agnuside enhances UCP1 protein abundance and mitochondrial respiratory capacity in brown and beige adipocytes independent of differentiation. (A) Schematic overview of the compound screening workflow. Immortalized brown preadipocytes were differentiated for 6 days and treated with individual compounds (10 µm) for 12 h. (B) Quantification of UCP1 protein levels from the western blot results shown in Figure S1A (*n* = 3 per group). Cells were treated with 64 compounds listed in Table S1 as in (A), followed by densitometric analysis. (C) Chemical structure of agnuside (AGN). (D) Immortalized brown adipocytes at day 6 of differentiation were treated with AGN at the indicated concentrations for 12 h. UCP1 protein expression is shown by western blot (left), with densitometric quantification on the right (*n* = 3 per group). (E) Basal, uncoupled, and maximal oxygen consumption rates (OCR) in cells treated as in (D) (*n* = 6–8 per group). The complete OCR trace is presented in Figure S1G. (F) Primary brown adipocytes differentiated from neonatal mouse interscapular BAT were treated with AGN at the indicated concentrations for 24 h. UCP1 protein expression is shown, with densitometric quantification on the right (*n* = 4 per group). (G) Basal, uncoupled, and maximal OCR in primary brown adipocytes treated as in (F) (*n* = 6–8 per group). The complete OCR trace is presented in Figure S1I. (H) Primary beige adipocytes differentiated from inguinal WAT of 2‐week‐old mice were treated with 50 µm AGN for 24 h. UCP1 protein expression is shown, with densitometric quantification on the right (*n* = 3 per group). (I) Basal, uncoupled, and maximal OCR in primary beige adipocytes treated as in (H) (*n* = 6–8 per group). The complete OCR trace is presented in Figure S1M. (J, L) Representative Oil Red O staining of primary brown adipocytes (J) and primary beige adipocytes (L). Cells were differentiated in the presence of 50 µm AGN, with medium replenished every 2 days. Quantification of lipid droplet accumulation is shown on the right (*n* = 5 per group). (K, M) Western blot analysis of primary brown adipocytes (K) and primary beige adipocytes (M), with densitometric quantification shown on the right (*n* = 3 per group). (N, O) Basal, uncoupled, and maximal OCR in primary brown adipocytes (N) and primary beige adipocytes (O) treated as in (J, L) (*n* = 6–8 per group). The complete OCR trace is presented in Figure S2C,E. All experiments were independently repeated three times with consistent results. Data are presented as mean ± SEM of biologically independent samples. Statistical significance was determined by one‐way ANOVA (D–G) or two‐tailed unpaired Student's *t*‐test (H–O). *p < 0.05, **p < 0.01, ***p < 0.001.

To identify compounds capable of modulating thermogenic capacity, we screened a library of 64 naturally occurring iridoid glycosides (Table S1) in differentiated brown adipocytes using UCP1 protein abundance as the primary phenotypic readout (Figure 1A,B). Among the tested compounds, gentiopicroside, aucubin, and valepotriate significantly suppressed UCP1 expression, consistent with previous reports (Figure 1B; Figure S1A, Table S1). In contrast, agnuside (AGN) uniquely induced a robust increase in UCP1 protein expression, reaching levels comparable to the classical activator forskolin (Figure 1B,C; Figure S1A, Table S1). Agnuside has been reported to possess anti‐inflammatory, antioxidant, hepatoprotective, and metabolic benefits in ovariectomized rat models of menopause, yet its direct role in adipose tissue has not been clearly defined [21].

We next evaluated the cytotoxicity of agnuside across multiple adipocyte precursor cell types. Even at concentrations up to 200 µm, agnuside did not impair cell proliferation or viability in immortalized brown adipocytes, primary brown adipocytes, or primary white adipocyte precursors (Figure S1B‐D). Dose‐response analyses revealed significant induction of UCP1 protein abundance at concentrations as low as 5 µm, with maximal effects observed at 20 µm, indicating a concentration‐dependent response with a plateau phase (Figure 1D). Oil Red O staining showed only a modest reduction in lipid droplet accumulation following treatment (Figure S1E). Notably, comprehensive qPCR profiling demonstrated that agnuside did not significantly alter the transcription of genes involved in thermogenesis (*Ucp1*), adipocyte differentiation (*Pparɣ2*, *Fabp4*), mitochondrial biogenesis (*Pgc‐1α*, *Pgc‐1β*, *Tfam*), lipid metabolism (*Atgl*, *Hsl*, *Mgll*, *Acadm*, *Cpt1b*), mitochondrial assembly (*Chchd3*), or electron transport chain components (*Sdhb*), indicating a predominantly post‐transcriptional mode of action (Figure S1F).

Mitochondrial respiratory function was subsequently evaluated using Seahorse extracellular flux analysis. Agnuside increased basal, maximal, and uncoupled oxygen consumption rate (OCR), indicating reinforcement of mitochondrial respiratory capacity and oxidative phosphorylation efficiency (Figure 1E; Figure S1G). These observations were recapitulated in primary brown adipocytes, where agnuside treatment increased UCP1 protein expression and respiratory capacity without altering thermogenic or lipid metabolic gene transcription (Figure 1F,G; Figure S1H–J). In beige adipocytes differentiated from inguinal white adipose tissue, agnuside robustly induced the beige‐selective markers *Tbx1* and *Tmem26*, accompanied by increased UCP1 protein abundance (UCP1 immunoblot is shown in Figure S1L), reduced lipid droplet accumulation, and significantly enhanced mitochondrial respiratory capacity (Figure 1H,I; Figure S1K–N). As observed in brown adipocytes, transcriptional programs related to thermogenesis and metabolism remained largely unaffected (Figure S1N).

To exclude potential effects on adipocyte differentiation, agnuside was administered throughout the entire differentiation process of primary brown and beige adipocytes (Figure S2A). Prolonged exposure did not alter lipid accumulation or the expression of key differentiation markers, including *Pparγ*, *Fabp4*, and *Pgc‐1α*, as confirmed by Oil Red O staining and gene expression analysis (Figure 1J–M; Figure S2B,D). Nevertheless, agnuside consistently elevated UCP1 protein abundance and mitochondrial respiratory capacity (Figure 1K,M–O; Figure S2C,E), mirroring the effects observed following short‐term treatment. These results demonstrate that agnuside enhances mitochondrial oxidative capacity and selectively elevates UCP1 protein abundance through post‐transcriptional mechanisms, without perturbing adipocyte differentiation or thermogenic gene expression.

### 2.2 Agnuside Selectively Potentiates Brown Adipose Thermogenesis Under Cold‐Induced Metabolic Stress

**FIGURE 2:**
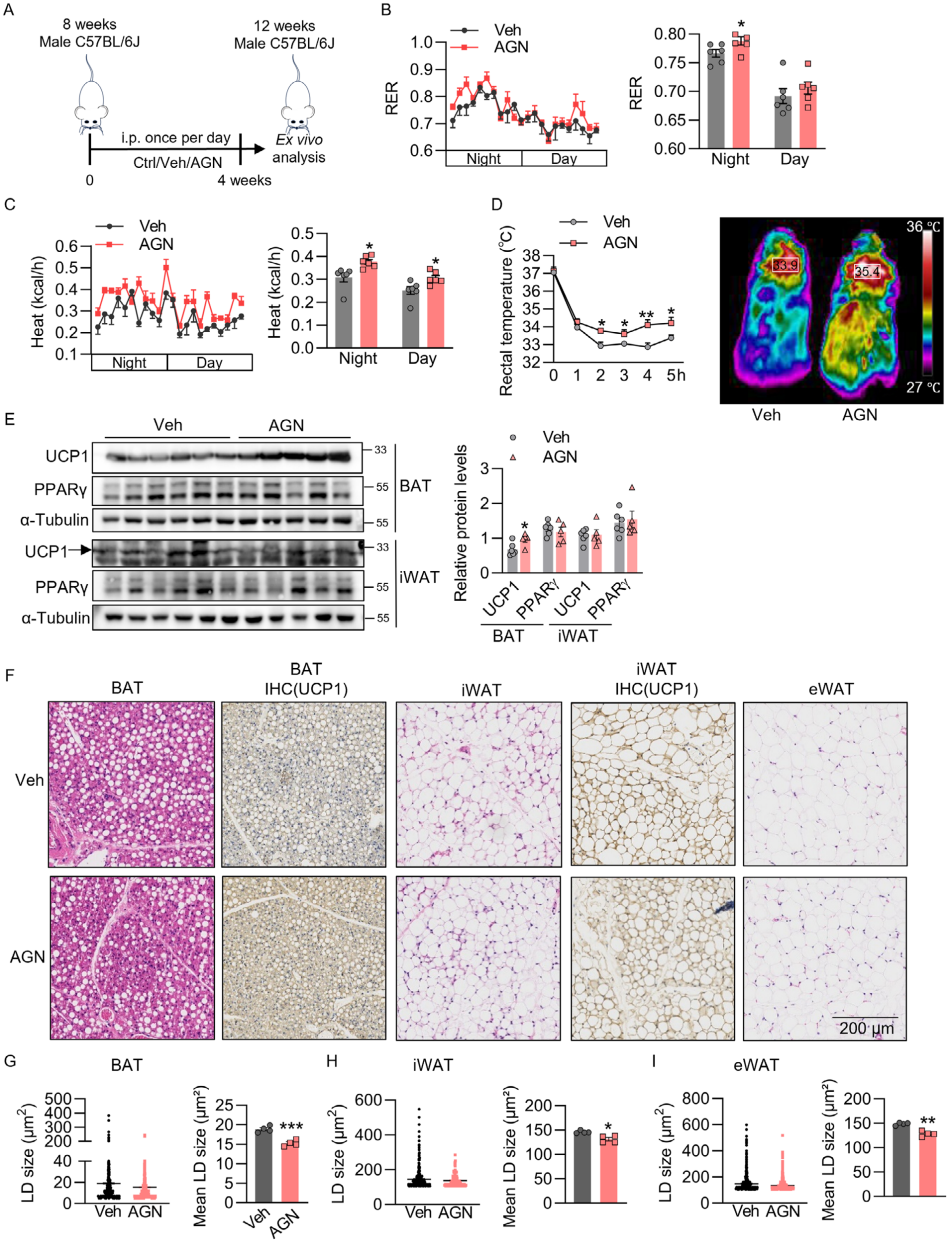
Agnuside selectively potentiates brown adipose thermogenesis and improves cold tolerance in vivo. (A) Schematic of the experimental protocol. Eight‐week‐old male C57BL/6J mice were divided into three groups: untreated control (Ctrl), vehicle‐treated (Veh), and agnuside‐treated (AGN). Mice in the AGN group received daily intraperitoneal injections of AGN (10 mg/kg), while the vehicle group received an equivalent volume of solvent, for four weeks under chow feeding before phenotypic analyses. (B,C) Respiratory exchange ratio (RER) (B) and energy expenditure (heat production) (C) in mice treated as in (A) (*n* = 6 per group), with quantification shown on the right. (D) Rectal temperature profiles of mice from (A) during acute cold exposure at 4°C for 5 h, recorded at 1 h intervals (*n* = 6 per group). Representative infrared thermographic images of dorsal surface temperature after 5 h of cold exposure are shown on the right. (E) Western blot analysis of protein expression in BAT and iWAT from mice treated as in (D), with densitometric quantification shown on the right (*n* = 5–6 per group). (F) Representative H&E staining and UCP1 immunohistochemistry (IHC) of BAT, iWAT, and eWAT from mice treated as in (D). Scale bar, 200 µm. (G–I) Lipid droplet size distribution in BAT (G), iWAT (H), and eWAT (I), quantified from images in (F). *n *= 2000 droplets each group in (G), and *n* = 500 droplets each group in (H, I). Pooled individual droplets are shown on the left, and the mean lipid droplet size per mouse is shown on the right (*n* = 4 per group). All experiments were independently repeated two times with consistent results. Data, except for the left panels in (G–I), are presented as mean ± SEM of biologically independent samples. Statistical significance was determined by two‐tailed unpaired Student's *t‐*test (B, E), ANCOVA with lean mass as a covariate (C), two‐way ANOVA (D), or two‐tailed Mann–Whitney test (G–I). *p < 0.05, **p < 0.01, ***p < 0.001.

Building upon our in vitro findings showing that agnuside enhances mitochondrial respiratory capacity and UCP1 protein abundance, we next investigated whether these molecular priming effects translate into functional thermogenic and metabolic responses in vivo. Eight‐week‐old C57BL/6J mice received daily intraperitoneal injections of agnuside for four weeks, alongside vehicle‐treated and untreated control groups (Figure 2A). Throughout the treatment period, body weight remained stable, and serum biochemical indices, including aspartate aminotransferase (AST), alanine aminotransferase (ALT), alkaline phosphatase (ALP), direct bilirubin (DBil), total bilirubin (TBil), blood urea nitrogen (BUN), and creatinine (Cr), showed no significant differences among groups (Figure S3A–H). Histological examination of the heart, liver, spleen, and kidney revealed comparable H&E staining patterns, collectively confirming the absence of overt systemic toxicity (Figure S3I).

Indirect calorimetry revealed that, despite comparable food intake, intestinal lipid absorption, and fecal lipid content, agnuside‐treated mice exhibited significantly increased oxygen consumption (VO_2_), carbon dioxide production (VCO_2_), heat generation, and respiratory exchange ratio (RER) during the dark phase, indicating a time‐dependent enhancement of whole‐body oxidative metabolism (Figure 2B,C; Figure S4A–F). In contrast, no differences were observed in the mass or histological architecture of brown adipose tissue (BAT), inguinal white adipose tissue (iWAT), or epididymal white adipose tissue (eWAT) (Figure S4G–K). Genomic DNA content in BAT and iWAT likewise remained unchanged, suggesting that agnuside did not influence adipocyte number or differentiation status (Figure S4L). UCP1 and PPARγ protein levels in both BAT and iWAT were comparable between groups, indicating that agnuside does not markedly activate thermogenic programs when basal energy demand is low (Figure S4M,N).

We therefore examined thermogenic performance under conditions of elevated energetic stress by subjecting mice to acute cold exposure (4°C). Agnuside‐treated mice displayed enhanced thermoregulatory capacity, maintaining significantly higher core body temperatures than control animals (Figure 2D). After five hours of cold challenge, dorsal skin temperature in the agnuside group was approximately 1.5°C higher than that of controls (Figure 2D). This improved thermal defense was accompanied by a robust increase in UCP1 protein abundance and marked lipid droplet mobilization in BAT, whereas UCP1 expression in iWAT remained unchanged, with only modest reductions in lipid droplet accumulation in iWAT and eWAT (Figure 2E–I). Consistent with our in vitro observations, transcript levels of thermogenic and adipogenic genes in both BAT and iWAT showed no systematic alterations, supporting a predominantly post‐transcriptional mode of regulation (Figure S4O,P). These results indicate that agnuside enhances systemic oxidative metabolism under basal conditions while selectively potentiating bona fide thermogenic responses only when energetic demand is elevated.

### 2.3 Agnuside Protects Against Diet‐Induced Obesity by Priming Thermogenic Responsiveness

Having established that agnuside primes thermogenic responsiveness under acute cold stress, we next examined whether this enhanced mitochondrial and thermogenic capacity confers sustained metabolic benefits under chronic nutrient excess. Six‐week‐old C57BL/6J mice were fed a high‐fat diet (HFD) for four weeks to induce early obesity, followed by twelve additional weeks of HFD feeding with daily intraperitoneal administration of agnuside (Figure S5A). Vehicle‐treated and untreated mice exhibited comparable body weights, and no differences were observed among groups in serum ALT, AST, ALP, DBil, TBil, BUN, or creatinine levels (Figure S5B–I). Histological analyses of major organs (heart, liver, spleen, and kidney) revealed no pathological alterations, indicating good tolerability of long‐term agnuside treatment (Figure S5J). Food intake, intestinal lipid absorption, and fecal lipid content were indistinguishable between vehicle‐ and agnuside‐treated mice (Figure S6A‐C).

**FIGURE 3:**
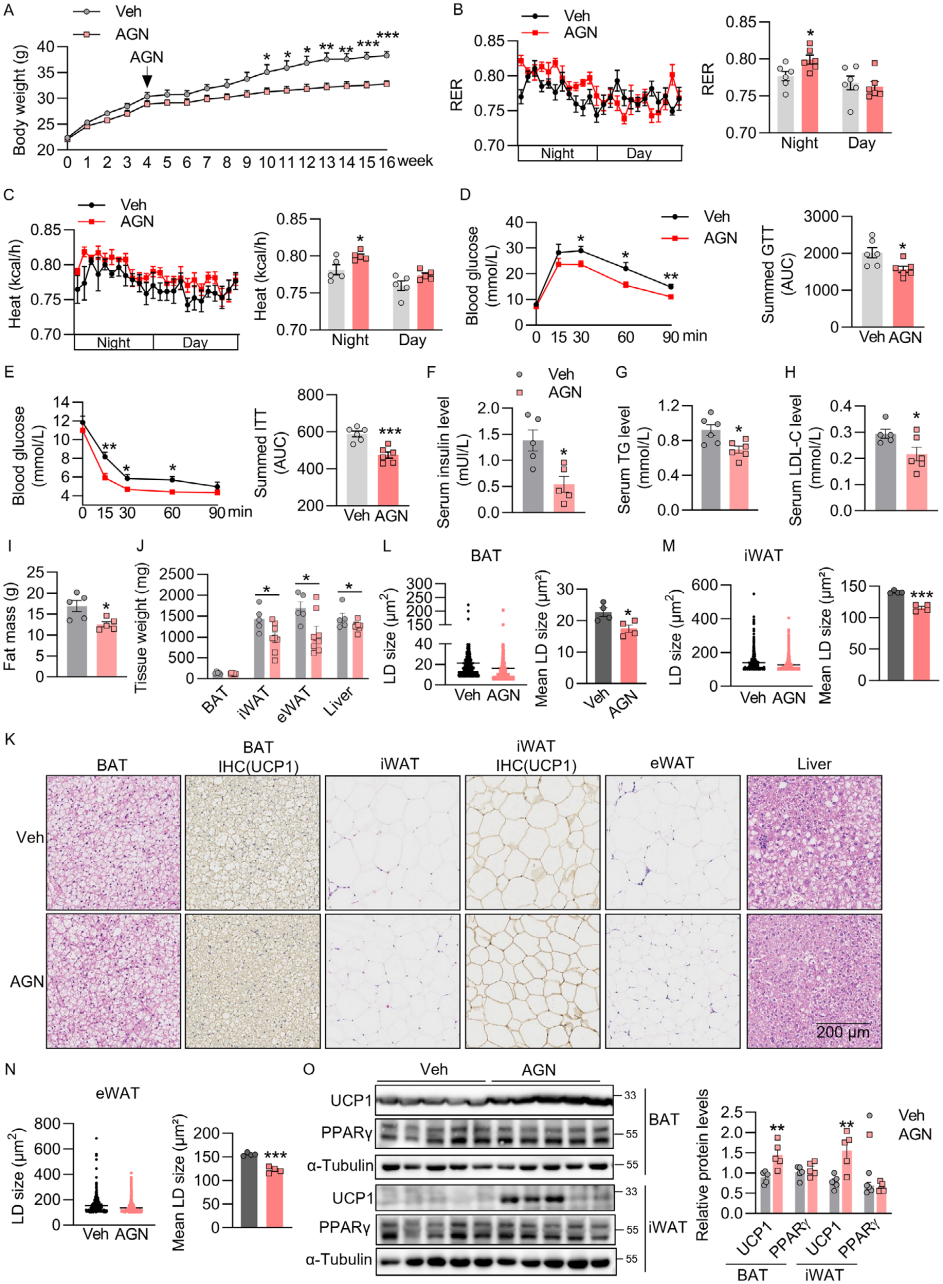
Agnuside limits diet‐induced obesity and improves systemic metabolic homeostasis during chronic high‐fat feeding. (A) Body weight trajectories of male C57BL/6J mice. Six‐week‐old mice were fed an HFD for 4 weeks, followed by daily intraperitoneal injections of AGN (7.5 mg/kg) or Veh while HFD feeding was continued for an additional 12 weeks. Body weight was recorded weekly (*n* = 6 per group). (B‐C) RER (B) and energy expenditure (C) in mice treated as in (A) (*n* = 5 per group). (D, E) Glucose tolerance test (GTT) (D) and insulin tolerance test (ITT) (E) in mice treated as in (A) (*n* = 6 per group). (F–H) Serum insulin (F) (*n* = 5 per group), triglycerides (TG) (G) (*n* = 6 per group), and LDL‐C (H) (*n* = 5–6 per group) in mice treated as in (A). (I) Fat mass measured using a body composition analyzer in mice treated as in (A) (*n* = 5 per group). (J) Weights of BAT, iWAT, eWAT, and liver from mice treated as in (A) (*n* = 5 per group). (K) Representative H&E staining of BAT, iWAT, eWAT, and liver from mice treated as in (A). Scale bar, 200 µm. (L–N) Lipid droplet size distribution in BAT (L), iWAT (M), and eWAT (N), quantified from images in (K). *n* = 2000 each group in (L), and *n* = 500 each group in (M,N). Pooled individual droplets are shown on the left, and the mean lipid droplet size per mouse is shown on the right (*n* = 4 per group). (O) Western blot analysis of protein expression in BAT and iWAT from mice treated as in (A), with densitometric quantification shown on the right (*n* = 5 per group). All experiments were independently repeated two times with consistent results. Data, except for the left panels in (L–N), are presented as mean ± SEM of biologically independent samples. Statistical significance was determined by two‐tailed unpaired Student's *t*‐test (A,B,D–J,O), ANCOVA with lean mass as a covariate (C), or two‐tailed Mann–Whitney test (L–N). *p < 0.05, **p < 0.01, ***p < 0.001.

From week 10 onward, agnuside‐treated mice showed a progressive reduction in body weight, reaching an approximate 6 g difference by week 16 (Figure 3A). Indirect calorimetry revealed sustained increases in nocturnal VO_2_, VCO_2_, heat production, and RER, reflecting agnuside selectively enhances energy expenditure during the active phase, rather than globally elevating basal metabolism (Figure 3B,C; Figure S6D,E). Accordingly, agnuside markedly improved glucose tolerance and insulin sensitivity, accompanied by reduced fasting insulin levels (Figure 3D–F). Enhanced Akt phosphorylation across adipose depots and liver further supported improved systemic insulin signaling (Figure S6F). Serum triglycerides (TG) and low‐density lipoprotein cholesterol (LDLC) were significantly reduced, whereas high‐density lipoprotein cholesterol (HDLC) and total cholesterol (TC) remained unchanged (Figure 3G,H; Figure S6G,H).

Body composition analysis demonstrated a pronounced reduction in fat mass with preservation of lean mass (Figure 3I; Figure S6I). Consistently, the weights of iWAT, eWAT, and liver were significantly decreased (Figure 3J). Histological examination revealed smaller adipocytes and reduced lipid accumulation in adipose tissues, accompanied by attenuated expression of inflammatory mediators, including *Tnfα*, *Ccl5*, and *Col1a1* in eWAT, as well as diminished hepatic *Tnfα* expression (Figure 3K–N; Figure S6J,K). Consistently, *Lep* expression decreased while *AdipoQ* expression increased in eWAT, indicating a shift toward a metabolically favorable adipokine profile (Figure S6J). At the molecular level, transcription of thermogenic and oxidative genes, including *Ucp1*, *Cidea*, *Cox7a1*, *Cpt1b*, in BAT and iWAT remained largely unchanged (Figure S6L,M). In contrast, UCP1 protein abundance was markedly elevated in both depots, including nearly a twofold increase in iWAT, without alterations in PPARγ expression (Figure 3O). Collectively, these findings demonstrate that agnuside improves metabolic homeostasis and attenuates diet‐induced weight gain during chronic high‐fat feeding.

### 2.4 Agnuside Promotes Thermogenesis Through Mitochondrial Assembly Remodeling

**FIGURE 4:**
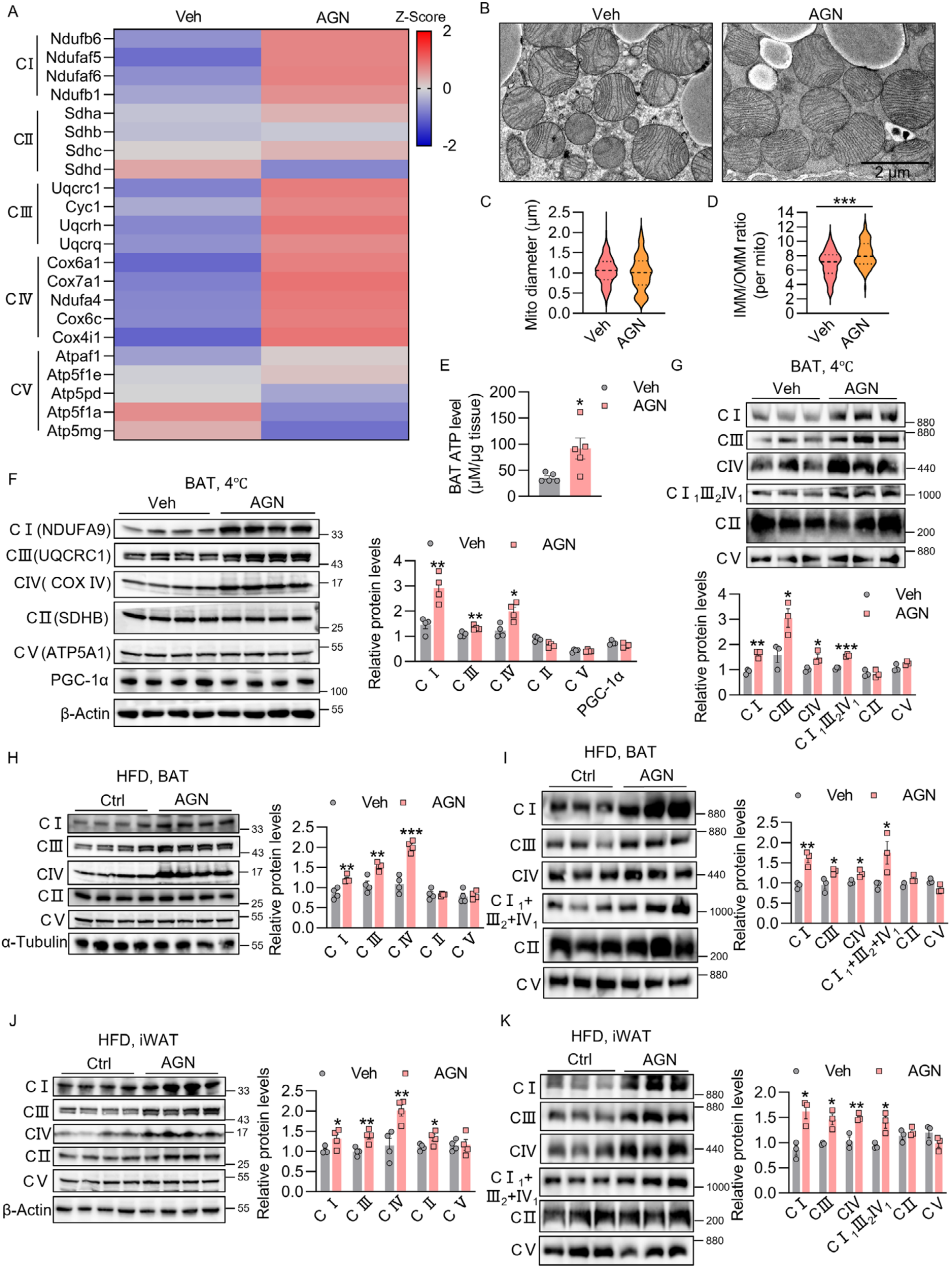
Agnuside reinforces mitochondrial respiratory complex assembly and cristae architecture in thermogenic adipose tissue. (A) Heatmap of protein abundance in BAT from mice exposed to acute cold (4°C, 5 h). Eight‐week‐old male C57BL/6J mice received daily intraperitoneal injections of AGN (7.5 mg/kg) for 4 weeks before cold exposure. Proteomic data were z‐score‐normalized and visualized as heatmaps (*n *= 4 per group). (B) Representative transmission electron microscopy (TEM) images of BAT mitochondria from mice treated as in (A). Scale bar, 2 µm. (C,D) Quantification of mitochondrial diameter (C) and cristae abundance (D) from TEM images shown in (B). *n* = 300 mitochondria each group in (C), and *n* = 80 mitochondria each group in (D). (E) ATP content in BAT tissues from mice treated as in (A) (*n* = 5 per group). (F) Western blot analysis of representative subunits of mitochondrial respiratory complexes in BAT from mice treated as in (A), with densitometric quantification on the right (*n* = 4 per group). (G) Blue native‐PAGE (BNPAGE) analysis of isolated BAT mitochondria from mice in (A), with quantification of respiratory complexes shown below (*n* = 3 per group). (H,J) Western blot analysis of representative subunits of mitochondrial respiratory complexes in BAT (H) and iWAT (J) from HFD‐fed mice treated with AGN, with quantification shown on the right (*n* = 4 per group). (I,K) BN‐PAGE analysis of mitochondria isolated from BAT (I) and iWAT (K) from HFD‐fed mice treated with AGN, with quantification shown on the right (*n* = 3 per group). All experiments were independently repeated two times with consistent outcomes. Data, except for panels (A,C,D), are presented as mean ± SEM of biologically independent samples. Statistical significance was determined by a two‐tailed unpaired Student's *t*‐test (D–K). *p < 0.05, **p < 0.01, ***p < 0.001.

To define the molecular basis underlying agnuside‐enhanced thermogenic responsiveness, we performed proteomic profiling of BAT from mice subjected to acute cold exposure. This analysis revealed coordinated upregulation of multiple assembly factors associated with mitochondrial complexes I, III, and IV, while their corresponding transcript levels remained largely unchanged, indicating post‐transcriptional reinforcement of respiratory machinery (Figure 4A; Figure S7A). Consistent with these proteomic results, transmission electron microscopy demonstrated that agnuside did not alter mitochondrial abundance or size but markedly increased cristae density in BAT, a structural hallmark of enhanced oxidative capacity and inner membrane expansion (Figure 4B–D; Figure S7B). In parallel, BAT ATP content was significantly elevated (Figure 4E). Immunoblotting further confirmed increased protein abundance of representative subunits from complexes I (NDUFA9), III (UQCRC1), and IV (COX4), whereas complex II (SDHB), complex V (ATP5A1), and the mitochondrial biogenesis regulator PGC‐1α remained unchanged, supporting selective optimization of existing respiratory architecture rather than de novo mitochondrial biogenesis (Figure 4F). Blue native PAGE further demonstrated enhanced assembly of complexes I, III, and IV together with increased formation of the I_1_III_2_IV_1_ respirasome, while complexes II and V were unaffected (Figure 4G).

Notably, this mitochondrial reinforcement exhibited demand‐dependent and depot‐selective characteristics. In cold‐exposed iWAT, agnuside selectively increased NDUFA9 protein levels without broader changes in assembly factors or transcripts, consistent with the lower basal oxidative capacity of this depot (Figure S7C‐E). In contrast, in HFD‐fed mice subjected to chronic agnuside treatment, both BAT and iWAT exhibited elevated protein abundance of representative subunits from complexes I, III, and IV, as detected by immunoblotting (Figure 4H,J). Consistently, blue native PAGE revealed enhanced assembly of complexes I, III, and IV together with increased formation of the I_1_III_2_IV_1_ supercomplex, whereas complexes II and V remained unchanged (Figure 4I,K). When mice were maintained at 22°C, agnuside failed to induce cristae remodeling or broad respiratory complex assembly, producing only modest increases in NDUFA9, in parallel with the absence of UCP1 recruitment when thermogenic demand is minimal (Figure S7F–I).

In vitro, primary brown and beige adipocytes treated with agnuside recapitulated the cold‐challenge phenotype, exhibiting marked upregulation of complexes I, III, and IV and increased mitochondrial membrane potential (Figure S7J–M), reflecting cell‐autonomous mitochondrial structural priming in the absence of systemic gating constraints. Collectively, these findings demonstrate that agnuside enhances thermogenesis by post‐transcriptionally reinforcing mitochondrial respiratory chain assembly and cristae architecture, thereby establishing a structurally primed mitochondrial state that supports thermogenic activation in a demand‐dependent manner.

### 2.5 Agnuside Directly Targets NDUFAF6 to Enhance Its Stability via Inhibition of Ubiquitination

**FIGURE 5:**
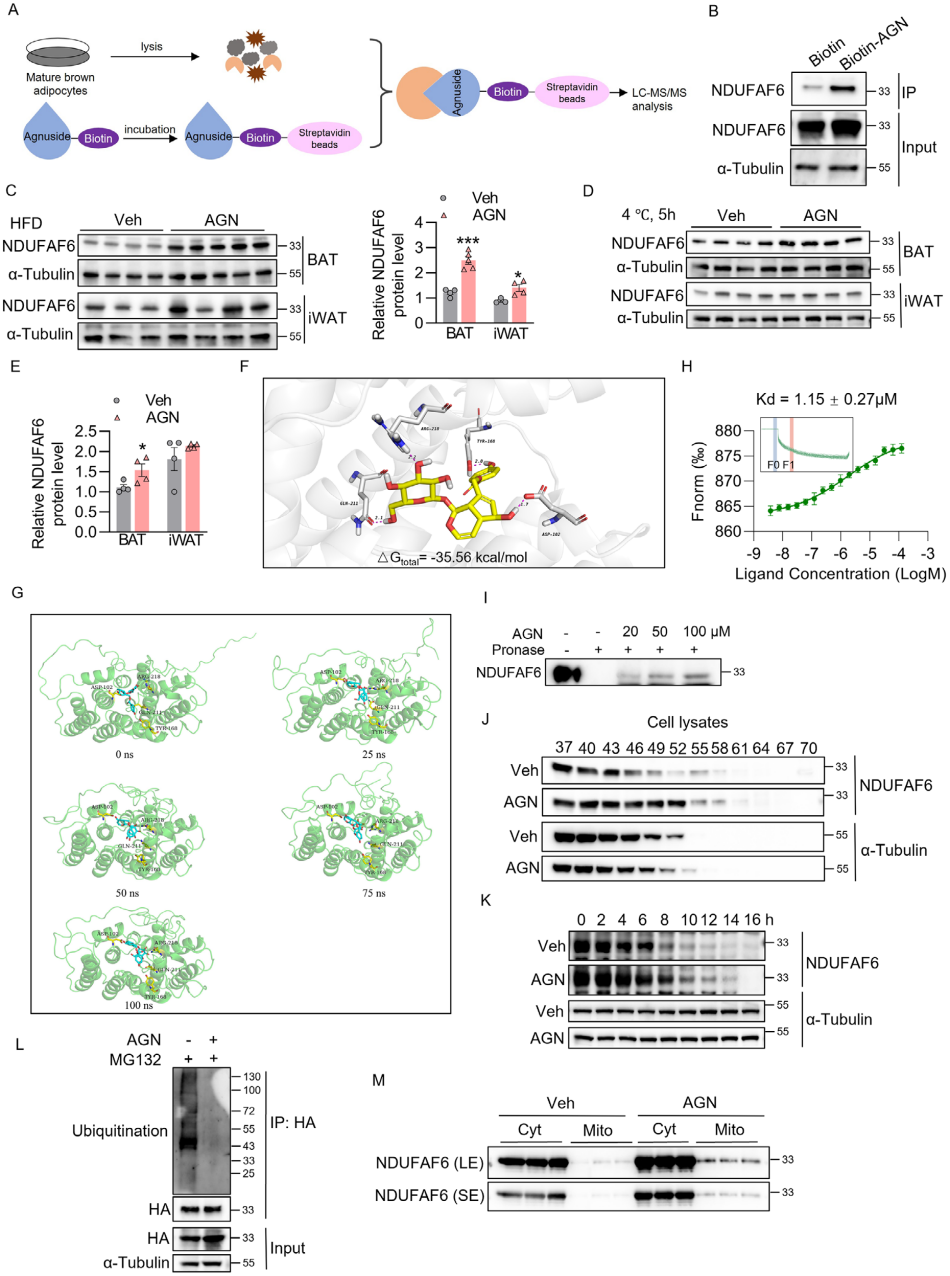
Agnuside directly binds and stabilizes NDUFAF6 by suppressing its ubiquitination. (A) Schematic illustration of the biotinylated AGN pull‐down assay. Biotin‐conjugated AGN was incubated with lysates of mature brown adipocytes, with unconjugated biotin as a negative control. Streptavidin‐coated beads were used for affinity purification, and precipitated proteins were analyzed by LC‐MS/MS to identify AGN‐interacting partners. (B) Validation of the AGN‐NDUFAF6 interaction. Mature brown adipocyte lysates were incubated with biotin‐conjugated AGN, and NDUFAF6 in the precipitates was detected by western blotting. (C) Western blot analysis of NDUFAF6 protein levels in BAT and iWAT from HFD‐fed mice treated with AGN, with quantification shown on the right (*n* = 4–5 per group). (D,E) Western blot analysis of NDUFAF6 protein levels in BAT and iWAT from mice treated with AGN and subjected to acute cold exposure. Quantification is shown in (E) (*n* = 4 per group). (F) Molecular docking of mouse NDUFAF6 with AGN. The protein backbone is shown in grey, nitrogen atoms in blue, oxygen atoms in red, and hydrogen atoms in white; AGN is represented as yellow sticks. Hydrogen bonds (purple dashed lines) were observed at Asp102, Tyr168, Gln211, and Arg218, with distances of 1.7, 2, 2.1, and 2.2 Å, respectively. Δ*G*_total_ = −35.56 kcal/ mol. (G) Molecular dynamics simulations of the AGN‐NDUFAF6 complex highlighting interactions centered on Asp102, Tyr168, Gln211, and Arg218 over a 100‐ns trajectory. (H) Binding affinity of AGN to NDUFAF6 determined by MST (*n* = 3 per group). The inset shows thermophoretic movement of fluorescently labeled proteins. Kd represents the dissociation constant. (I) DARTS analysis of NDUFAF6 in mature brown adipocytes treated with increasing concentrations of AGN. (J) CETSA of NDUFAF6 in mature brown adipocytes following AGN treatment across a temperature gradient. (K) Half‐life analysis of NDUFAF6. Mature brown adipocytes were treated with AGN (50 µm, 24 h), followed by cycloheximide (50 µm) for the indicated times. NDUFAF6 protein levels were assessed by western blotting. (L) AGN suppresses ubiquitination of cytosolic NDUFAF6. Brown adipocyte precursors were transduced with HA‐tagged NDUFAF6 lentivirus, differentiated, and treated with MG132 (10 µm, 6 h). Cytosolic fractions were immunoprecipitated with HA‐beads, and ubiquitination was detected by anti‐ubiquitin immunoblotting. (M) Western blot analysis of NDUFAF6 protein expression in cytosolic and mitochondrial fractions of BAT from mice treated as in (D). SE, short exposure; LE, long exposure. Note: The cytosolic fraction was obtained as the 10 000 g supernatant without additional ultracentrifugation, and may therefore include minor microsomal contamination. All experiments were independently repeated three times with consistent results. Data are presented as mean ± SEM of biologically independent samples. Statistical significance was determined by a two‐tailed unpaired Student's *t*‐test (C,E). *p < 0.05, ***p < 0.001.

Given that agnuside selectively reinforced mitochondrial respiratory chain assembly and cristae architecture without inducing mitochondrial biogenesis, we next sought to define its proximal molecular target mediating these post‐transcriptional effects. Because agnuside lacks lipophilic domains and mitochondrial targeting features (Figure 1C), we first tested whether it directly enters mitochondria. Time‐resolved mitochondrial fractionation followed by mass spectrometry failed to detect agnuside within mitochondrial compartments at any examined time point (Figure S8A), indicating that its action is mediated through extramitochondrial regulatory mechanisms. To identify cytosolic intermediates linking agnuside to mitochondrial remodeling, we synthesized a biotin‐conjugated agnuside probe and performed affinity purification in differentiated brown adipocytes (Figure 5A; Table S2). Proteomic profiling revealed 70 proteins uniquely associated with agnuside, among which the complex I assembly factor NDUFAF6 emerged as a prominent candidate (Table S3). Previous studies have shown that NDUFAF6 acts as an HSP90 co‐chaperone in the cytosol to stabilize nascent NDUFA10 and NDUFS3 during late‐stage folding, while following mitochondrial import it associates with the ND1 module and cooperates with NDUFS8 to drive early complex I assembly, thereby linking cytosolic proteostasis to respiratory chain organization [10, 11].

Direct binding between agnuside and NDUFAF6 was validated by pull‐down assays using biotin‐conjugated agnuside (Figure 5B). Consistent with functional activation, chronic agnuside administration markedly increased NDUFAF6 protein abundance in BAT and iWAT under HFD conditions. In contrast, neither NDUFAF6 nor representative respiratory complex subunits showed detectable changes in liver, heart, or skeletal muscle (Figure 5C; Figure S8B–D). Under acute cold exposure, agnuside robustly upregulated NDUFAF6 in BAT, whereas no apparent induction was observed in iWAT, liver, heart, or skeletal muscle (Figure 5D,E; Figure S8E). At 22°C, this AGN‐induced increase remained selectively confined to BAT (Figure S8F), mirroring the demand‐dependent thermogenic responses described above.

Molecular docking analysis further supported high‐affinity binding between agnuside and NDUFAF6, with stable hydrogen bond interactions involving residues Asp102, Tyr168, Gln211, and Arg218, with a calculated binding energy of −35.56 kcal/ mol (Figure 5F; Figure S9A). Molecular dynamics simulations confirmed the stability of this complex, with favorable RMSD, RMSF, Rg, SASA, and hydrogen‐bonding profiles (Figure 5G; Figure S9B–G). Consistent with structural predictions, Tyr168, Gln211, and Arg218 cluster within the same surface patch of NDUFAF6 that was identified by deep mutational scanning as highly sensitive to substitution, indicating its critical role as an interaction interface with complex I subunits such as NDUFS8. In contrast, Asp102 is positioned at the degenerated catalytic site of the prenyltransferase‐like fold, serving primarily as a structural scaffold rather than a catalytic residue [11]. Microscale thermophoresis (MST) measurements determined a dissociation constant (*K*_d_) of 1.15 ± 0.27 µm, consistent with high‐affinity binding (Figure 5H; Figure S9H).

Because small‐molecule binding often enhances protein stability, we next tested whether agnuside protects NDUFAF6 from degradation. Drug affinity responsive target stability (DARTS) assays revealed dose‐dependent resistance of NDUFAF6 to proteolysis following agnuside treatment (Figure 5I). Cellular thermal shift assays (CETSA) further demonstrated increased thermal stability of NDUFAF6 across a temperature gradient (Figure 5J). Pulse‐chase experiments further showed a prolonged NDUFAF6 half‐life in agnuside‐treated cells (Figure 5K). Mechanistically, ubiquitination assays using HA‐tagged NDUFAF6 revealed that agnuside markedly suppressed cytosolic ubiquitination of NDUFAF6 (Figure 5L). Subcellular fractionation of BAT following acute cold exposure demonstrated that NDUFAF6 is predominantly localized in the cytosolic compartment in control conditions, whereas agnuside treatment markedly increased its abundance in both cytosolic and mitochondrial fractions (Figure 5M; Figure S9I). Together, these results establish cytosolic NDUFAF6 as a direct and high‐affinity molecular target of agnuside. By inhibiting ubiquitin‐mediated degradation, agnuside stabilizes NDUFAF6, promotes its mitochondrial import, and thereby enhances respiratory chain assembly and mitochondrial structural remodeling.

### 2.6 NDUFAF6 Is Essential for Mitochondrial Integrity and Agnuside‐Driven Thermogenic Activation

**FIGURE 6:**
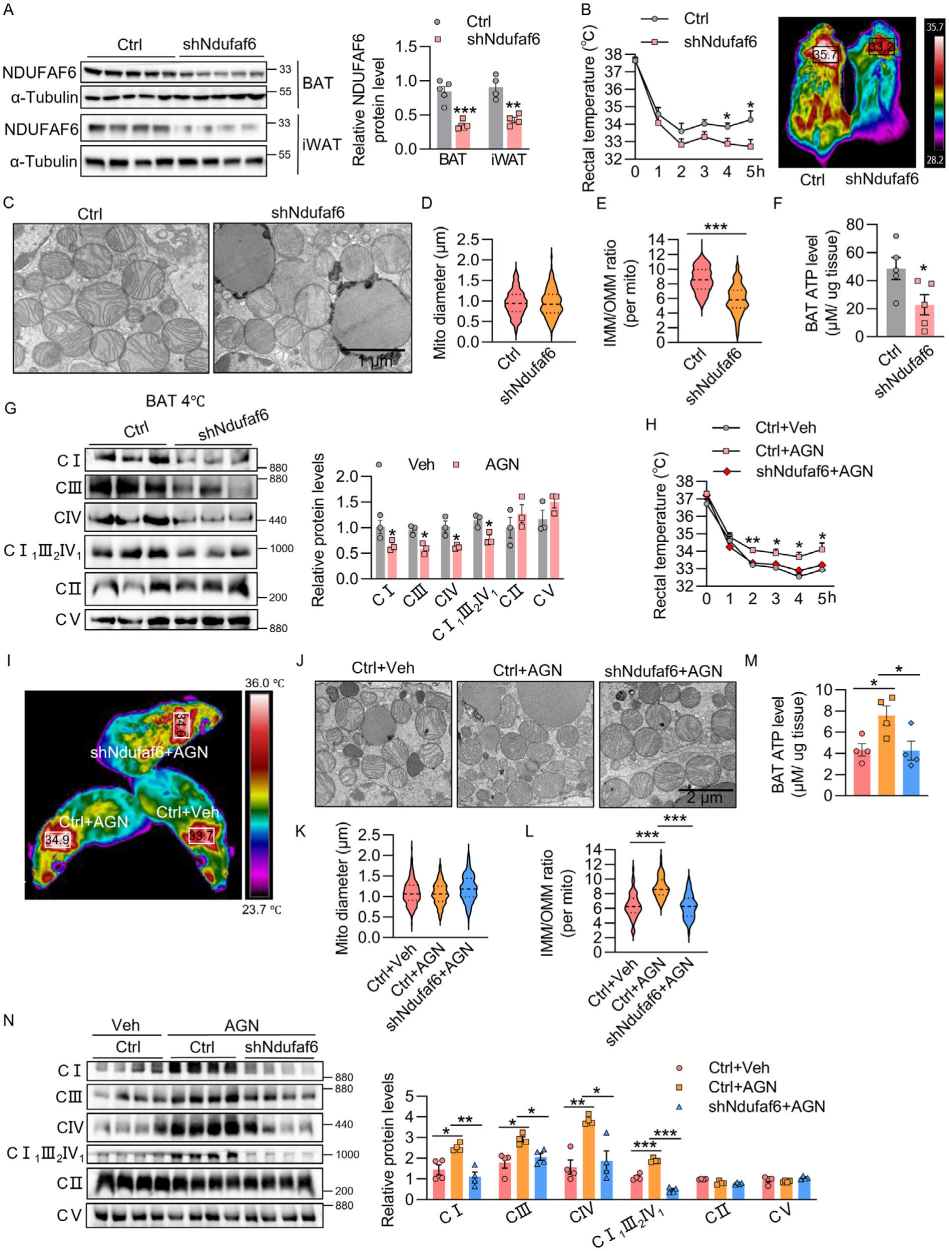
NDUFAF6 is required for mitochondrial integrity and agnuside‐driven thermogenic activation (A) Western blot analysis of *Ndufaf6* knockdown efficiency in BAT and iWAT, with quantification shown on the right (*n* = 4–5 per group). Adeno‐associated virus (AAV) expressing shRNA against *Ndufaf6* under the control of the *AdipoQ* promoter was locally injected into BAT and iWAT depots of 10‐week‐old male mice. Animals were maintained on normal chow for 4 weeks before phenotypic analyses. (B) Core body temperature in *Ndufaf6* knockdown mice during 4°C cold exposure, measured hourly for 5 h (*n* = 6–7 per group). Representative infrared thermography images of dorsal surface temperature were obtained after 5 h. (C) Representative TEM images of BAT from *Ndufaf6* knockdown mice following 4°C cold exposure for 5 h. (D,E) Quantification of mitochondrial diameter (D) and cristae abundance (E) from TEM images in (C). *n* = 250 mitochondria per group in (D); *n* = 70 mitochondria per group in (E). (F) ATP levels in BAT from mice treated as in (B) (*n* = 5 per group). (G) BN‐PAGE of mitochondria isolated from BAT of mice treated as in (B), with quantification of respiratory complexes shown in the right panels (*n* = 3 per group). (H–I) Core body temperature was recorded hourly for 5 h during cold exposure at 4°C (*n* = 6 per group) (H), and representative infrared thermography images of the dorsal surface were captured after the 5 h challenge (I). Ten‐week‐old *Ndufaf6* knockdown mice were treated with daily intraperitoneal injections of AGN (10 mg/kg) for 4 weeks prior to phenotypic analyses. (J) Representative TEM images of BAT from mice treated as in (H). (K,L) Quantification of mitochondrial diameter (K) and cristae abundance (L) from TEM images in (J). *n* = 200 mitochondria per group in (K); *n* = 60 mitochondria per group in (L). (M) ATP levels in BAT from mice treated as in (H) (*n* = 4 per group). (N) BN‐PAGE of mitochondria isolated from BAT of mice treated as in (H), with quantification of respiratory complexes shown in the right panels (*n* = 4 per group). All experiments were independently repeated two times with consistent results. Data, except for panels (D,E,K,L), are presented as mean ± SEM of biologically independent samples. Statistical significance was determined by two‐tailed unpaired Student's *t*‐test (A,E–G), two‐way ANOVA (B), or one‐way ANOVA (H,L–N). *p < 0.05, **p < 0.01, ***p < 0.001.

To establish the functional relevance of NDUFAF6 in adipose thermogenesis, we first examined its distribution across major fat depots and found that NDUFAF6 was highly enriched in BAT relative to iWAT and eWAT (Figure S10A). Selective depletion of *Ndufaf6* in BAT and iWAT using an *AdipoQ* promoter‐driven AAV‐shRNA achieved efficient knockdown of approximately 60%–70% (Figure 6A; Figure S10B).

When mice were maintained at 22°C, *Ndufaf6* deficiency did not alter body weight, adipose mass, or adipocyte morphology (Figure S10C–G). However, transmission electron microscopy revealed a marked reduction in mitochondrial cristae density in BAT, without changes in mitochondrial size (Figure S10H–J). Consistently, the protein abundance of the complex I subunit NDUFA9 was significantly decreased (Figure S10K,L). Indirect calorimetry further showed reduced nocturnal VO_2_, VCO_2_, and heat production, indicating impaired oxidative metabolism (Figure S10M–P).

Upon 4°C acute cold exposure, *Ndufaf6*‐deficient mice exhibited profound thermogenic impairment, characterized by rapid declines in core body temperature and reduced dorsal surface temperature (Figure 6B). BAT displayed increased lipid accumulation and persistent loss of cristae density without changes in mitochondrial number, accompanied by significantly reduced ATP levels (Figure 6C–F; Figure S11A–D). At the molecular level, *Ndufaf6* knockdown decreased UCP1 protein abundance and representative subunits of complexes I, III, and IV, while blue native PAGE confirmed defective assembly of these complexes and the I_1_III_2_IV_1_ supercomplex (Figure 6G; Figure S11E). In contrast, transcript levels of thermogenic genes and assembly factors remained largely unchanged (Figure S11F), supporting post‐transcriptional regulation.

Consistent with the role of NDUFAF6 as an HSP90 co‐chaperone, *Ndufaf6* knockdown reduced the protein abundance of NDUFA10 and NDUFS3 in BAT without affecting HSP90 levels, and weakened their association with HSP90 (Figure S11E,G). Subcellular fractionation further demonstrated reduced levels of these proteins in both cytosolic and mitochondrial compartments, establishing NDUFAF6 as a critical determinant of complex I proteostasis and incorporation (Figure S11H). In iWAT, *Ndufaf6* selectively impaired complex I components without affecting UCP1 or other respiratory complexes (Figure S11I). These defects were recapitulated in primary brown adipocytes, where *Ndufaf6* knockdown reduced respiratory complex abundance and ATP production (Figure S11J,K).

We next tested whether NDUFAF6 is required for agnuside‐mediated thermogenic enhancement. In primary brown adipocytes, *Ndufaf6* silencing abolished agnuside‐induced increases in UCP1, respiratory complex abundance, ATP production, and oxygen consumption (Figure S12A–G). In vivo, selective depletion of *Ndufaf6* in BAT and iWAT did not affect body weight or adipose mass following 4‐week agnuside administration, but eliminated the ability of agnuside to improve thermoregulation during cold exposure, as evidenced by persistently reduced core body temperature and dorsal skin temperature (Figure 6H,I; Figure S12H,I). Consistently, agnuside failed to promote mitochondrial cristae densification or restore ATP levels in BAT of *Ndufaf6*‐deficient mice, despite unchanged mitochondrial size (Figure 6J–M). At the molecular level, agnuside was unable to rescue the reduced abundance of UCP1 and representative subunits of complexes I, III, and IV caused by *Ndufaf6* knockdown, nor reverse the impairment of mitochondrial supercomplex assembly (Figure 6N; Figure S12J). Moreover, the stabilizing effects of agnuside on the NDUFAF6 client proteins NDUFA10 and NDUFS3 and their interaction with HSP90 were completely abolished (Figure S12J–L). Together, these results demonstrate that NDUFAF6 is indispensable for agnuside‐mediated mitochondrial remodeling and thermogenic activation.

### 2.7 NDUFAF6 Is Required for Agnuside‐Driven Mitochondrial Efficiency and Metabolic Benefits

**FIGURE 7:**
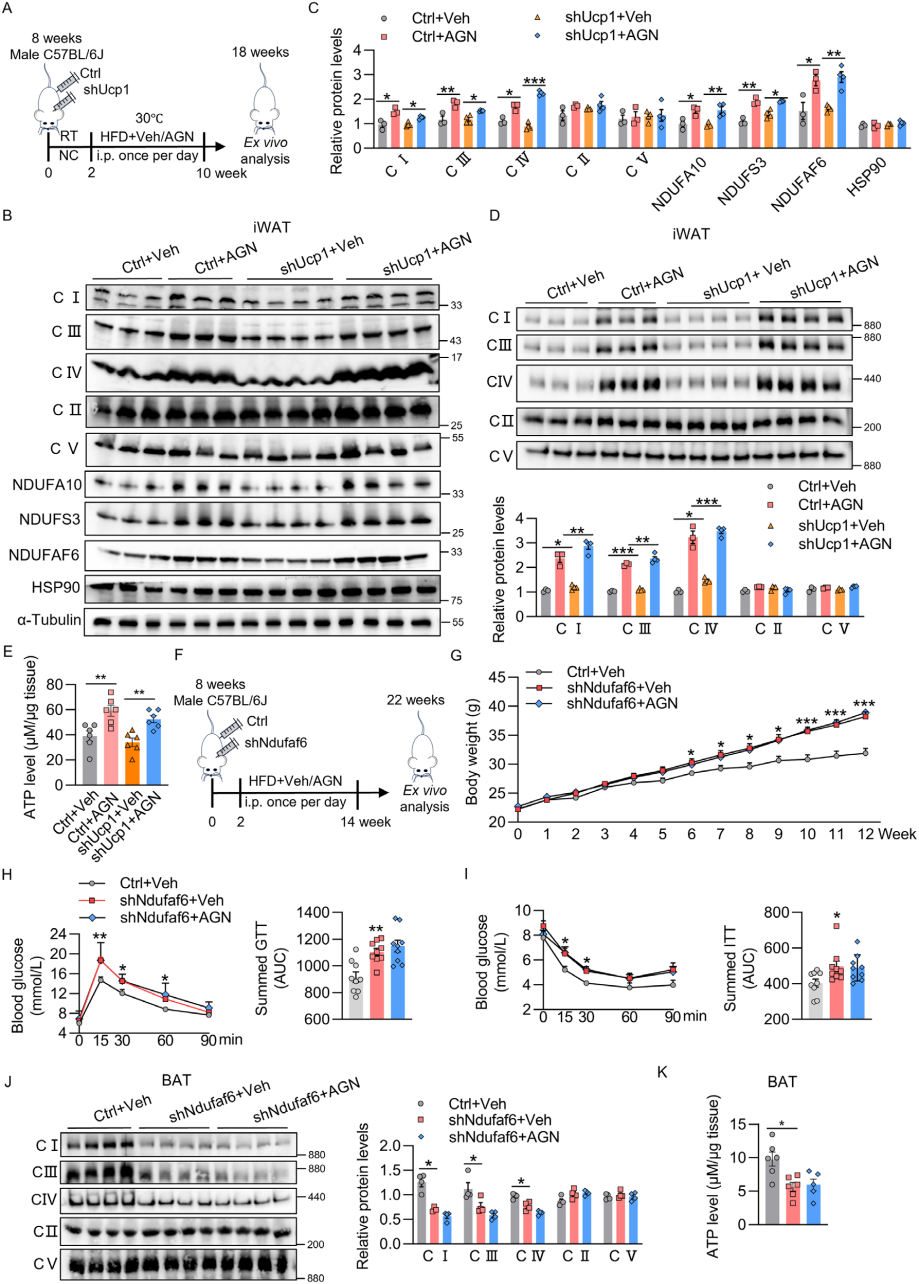
Agnuside improves mitochondrial efficiency and metabolic homeostasis independently of UCP1 but requires NDUFAF6. (A) Schematic of the experimental protocol. Eight‐week‐old male mice received AAV‐mediated *Ucp1* knockdown in BAT and iWAT, followed by 2 weeks of recovery at room temperature. Mice were then maintained at thermoneutrality (30°C) under HFD feeding with daily intraperitoneal injections of AGN (7.5 mg/kg) or vehicle for 8 weeks. (B,C) Western blot analysis of in iWAT (B), with quantification shown in (C) (*n* = 3–4 per group). (D) BN‐PAGE analysis of mitochondria isolated from iWAT, with quantification shown below (*n* = 3–4 per group). (E) ATP levels in iWAT from mice treated as in (A) (*n* = 6 per group). (F,G) Experimental scheme (F) and body weight trajectories (G) of mice. Eight‐week‐old mice received local injections of AAV expressing shRNA against *Ndufaf6* under the *AdipoQ* promoter into BAT and iWAT depots. Mice were maintained on standard chow at room temperature for 2 weeks to allow viral expression, followed by 12 weeks of high‐fat diet feeding with daily intraperitoneal injections of AGN (7.5 mg/kg) or Veh before phenotypic analyses. Body weight was recorded weekly (G) (*n* = 9 per group). (H,I) GTT (H) and ITT (I) analysis in mice treated as in (F) (*n* = 9 per group). (J) BN‐PAGE analysis of mitochondria isolated from BAT of mice treated as in (F), with quantification shown on the right (*n *= 4 per group). (K) ATP levels in BAT from mice treated as in (F) (*n* = 5–6 per group). All experiments were independently repeated two times with consistent results. Data are presented as mean ± SEM of biologically independent samples. Statistical significance was determined by one‐way ANOVA (C–E,G–K). *p < 0.05, **p < 0.01, ***p < 0.001.

To determine whether UCP1 is required for agnuside‐induced mitochondrial remodeling, we selectively silenced *Ucp1* in BAT and iWAT using AAV‐mediated shRNA under thermoneutral high‐fat diet conditions, in which basal thermogenic demand is minimized (Figure 7A). Despite efficient UCP1 depletion (>80%), agnuside treatment robustly increased the protein abundance of representative subunits from respiratory complexes I, III, and IV in iWAT, whereas complexes II and V remained unchanged (Figure 7B,C; Figure S13A). Consistently, BN‐PAGE analysis revealed enhanced mitochondrial supercomplex assembly together with a significant increase in ATP levels, particularly in iWAT (Figure 7D,E).

These mitochondrial effects occurred without changes in food intake, intestinal lipid absorption, or fecal lipid content (Figure S13B–D), indicating that agnuside‐driven metabolic remodeling was independent of nutrient handling. Although *Ucp1*‐deficient mice exhibited greater weight gain under thermoneutral high‐fat feeding, agnuside induced a comparable reduction in body weight in both control and *Ucp1*‐silenced mice (Figure S13E). Systemic metabolic benefits, including improved glucose tolerance and insulin sensitivity, were likewise largely preserved in *Ucp1*‐deficient animals (Figure S13F,G).

Adipose tissue analyses further showed that agnuside markedly reduced iWAT and eWAT mass in *Ucp1*‐silenced mice, accompanied by diminished lipid droplet accumulation (Figure S13H–L). In contrast, mitochondrial complex assembly in BAT remained largely unchanged under thermoneutral conditions (Figure S13M), suggesting that iWAT represents the principal depot mediating UCP1‐independent metabolic benefits when thermogenic drive is low. Consistent with the adipose tissue mitochondrial remodeling described above, indirect calorimetry in an independent thermoneutral HFD cohort showed that AGN‐treated mice exhibited higher basal metabolic rates before CL316,243 administration, as reflected by increased VO_2_, VCO_2_, and heat production. Following acute β3‐adrenergic stimulation, CL316,243 markedly increased VO_2_, VCO_2_, and heat production in vehicle‐treated mice, whereas AGN‐treated mice showed a relatively blunted metabolic response (Figure S14A–D). Consequently, post‐CL316,243 metabolic rates became comparable between the two groups. RER was not significantly altered by AGN treatment or CL316,243 stimulation (Figure S14A‐‐D). Together, these findings indicate that, rather than acting as an acute enhancer of β3‐adrenergic thermogenesis, AGN establishes a higher basal oxidative metabolic state under chronic metabolic stress, thereby supporting mitochondrial oxidative efficiency and systemic metabolic homeostasis independently of UCP1 under conditions of low thermogenic demand.

We next investigated whether the mitochondrial assembly factor NDUFAF6 is required for these UCP1‐independent metabolic effects. To this end, *Ndufaf6*‐deficient mice were subjected to high‐fat feeding and treated with agnuside (Figure 7F). Despite comparable food intake, lipid absorption, and fecal lipid excretion, *Ndufaf6*‐deficient mice displayed accelerated weight gain, and agnuside completely failed to reduce body weight or improve glucose tolerance and insulin sensitivity in these animals (Figure 7G–I; Figure S15A–C). Body composition analysis revealed markedly increased fat mass, accompanied by significant enlargement of BAT, iWAT, eWAT, and liver depots (Figure S15D,E). Consistently, all adipose depots exhibited pronounced adipocyte hypertrophy and elevated expression of inflammatory markers, including *Tnfα* and *IL‐6*, together with increased *Lep* and reduced *AdipoQ* expression in eWAT, reflecting severe metabolic deterioration (Figure S15F–K).

At the molecular level, *Ndufaf6* knockdown selectively reduced the protein abundance of respiratory complexes I, III, and IV in both BAT and iWAT, with minimal effects on complexes II and V (Figure S16A–D). BN‐PAGE analysis confirmed defective supercomplex assembly, accompanied by markedly reduced ATP production (Figure 7J,K; Figure S16E,F). Notably, agnuside failed to restore respiratory complex abundance, supercomplex formation, or ATP levels in *Ndufaf6*‐deficient tissues. Furthermore, the NDUFAF6 client proteins NDUFA10 and NDUFS3 were profoundly reduced in both cytosolic and mitochondrial fractions, and their stabilization by agnuside was completely abolished (Figure S16G,H). Collectively, these findings demonstrate that agnuside‐mediated metabolic improvements are preserved under thermoneutral conditions and *Ucp1* deficiency, but critically depend on NDUFAF6‐driven mitochondrial assembly.

## 3 Discussion

This study identifies AGN as a natural small molecule that enhances adipose thermogenic competence through selective stabilization of the mitochondrial complex I assembly factor NDUFAF6. Rather than directly activating canonical thermogenic transcriptional programs, AGN reinforces mitochondrial respiratory architecture at the post‐transcriptional level, thereby establishing a structurally and functionally primed mitochondrial state. This priming enables efficient thermogenic activation under elevated energetic demand while preserving physiological gating under basal conditions. Genetic ablation of *Ndufaf6* abolishes these effects across cellular and organismal contexts, establishing the AGN‐NDUFAF6 axis as a central determinant of mitochondrial efficiency and thermogenic responsiveness.

Mechanistically, our findings indicate that AGN primarily acts through cytosolic regulation of NDUFAF6. AGN directly binds NDUFAF6, suppresses its ubiquitination, prolongs its half‐life, and promotes mitochondrial import. Stabilized NDUFAF6 enhances the folding and preservation of nascent complex I subunits, including NDUFA10 and NDUFS3, through reinforced interaction with HSP90, thereby safeguarding early assembly intermediates prior to mitochondrial incorporation. This extramitochondrial quality‐control step selectively increases the assembly of complexes I, III, and IV and stabilizes the I_1_III_2_IV_1_ respirasome, leading to increased cristae density and ATP‐generating capacity without affecting complexes II and V or inducing mitochondrial biogenesis.

The selectivity of this assembly remodeling is consistent with established principles of respiratory chain organization. Early complex I (CI) assembly intermediates can function as structural scaffolds that nucleate the recruitment of complexes III and IV, thereby biasing respiratory chain organization toward I‐III‐IV supercomplex formation, whereas complexes II and V are not integral components of these modules [22]. Consistent with this model, studies in human cells indicate that respirasome biogenesis does not require prior completion of individual respiratory complexes but can instead be initiated by CI membrane‐arm intermediates that facilitate CIII and CIV incorporation [23]. Accordingly, stabilization of CI selectively biases respiratory chain organization toward I‐III‐IV assembly without altering CII or CV abundance. Supporting this framework, distinct classes of assembly and organizing factors have been shown to couple CI maturation to downstream respiratory chain architecture. MITRAC15/COA1 coordinates CI and CIV biogenesis during early assembly stages, and its loss selectively reduces both CI and CIV without affecting CII or CV, reflecting a biogenetic interdependence rather than direct supercomplex stabilization [24]. TIMMDC1, a bona fide CI assembly factor required for membrane‐arm maturation, serves as a permissive determinant for respirasome formation by stabilizing CI intermediates that subsequently enable incorporation of CIII and CIV [25]. In contrast, SCAF1/COX7A2L acts as a supercomplex‐specific organizer, directly stabilizing the CIII_2_‐CIV interaction and thereby promoting CI‐III‐IV respirasome integrity while leaving CII and CV unaffected [26]. Collectively, these examples provide a mechanistic rationale for our observation that AGN promotes CI stabilization and downstream I‐III‐IV assembly, while leaving CII and CV unaltered.

A defining feature of AGN action is its pronounced demand dependence, which helps reconcile mitochondrial remodeling with UCP1 recruitment and thermogenic output. At 22°C, AGN‐treated mice displayed increased nocturnal VO_2_, VCO_2_, and calculated heat production (Figure 2B,C; Figure S4D–F), indicating elevated whole‐body oxidative metabolism during the active phase. However, because locomotor activity and running distance were not recorded during indirect calorimetry, a contribution of altered dark‐phase activity or behavior cannot be excluded. We therefore interpret these data conservatively as increased nocturnal oxidative metabolism rather than direct evidence of enhanced BAT thermogenesis. Consistent with this interpretation, UCP1 abundance, mitochondrial ultrastructure, and respiratory complex assembly remain largely unchanged at 22°C, except for a modest increase in the complex I subunit NDUFA9 that did not translate into higher‐order respirasome remodeling (Figure S4M,N; Figure S7F–I). These findings indicate that AGN enhances mitochondrial oxidative efficiency without overtly engaging uncoupled thermogenesis under low thermogenic demand. In contrast, when energetic demand was elevated by acute cold exposure or chronic high‐fat feeding, AGN markedly amplified thermogenic and mitochondrial responses, including increased cristae densification, ATP production, UCP1 protein abundance, and lipid mobilization in BAT, with similar mitochondrial reinforcement extending to iWAT under sustained metabolic stress (Figure 2D–I, Figures 3 and 4).

The causal hierarchy underlying these effects is further resolved by thermoneutral *Ucp1* knockdown experiments (Figure 7A). Under conditions in which sympathetic tone and thermogenic drive are minimized, AGN preserves its ability to enhance respiratory complex I/III/IV assembly, respirasome formation, and ATP production, particularly in iWAT, while retaining substantial metabolic benefits at the whole‐body level (Figure 7B–E; Figure S13). These observations establish improved mitochondrial efficiency as a primary, UCP1‐independent mechanism of AGN action, with UCP1 serving as a secondary amplifier that becomes functionally engaged only under elevated energetic demand. Together, these findings position AGN as a mitochondrial priming agent that augments thermogenic responsiveness without overriding physiological gating mechanisms.

Beyond elucidating the mechanism of agnuside, this work expands the conceptual framework of mitochondrial regulation by highlighting assembly‐factor proteostasis as a modifiable determinant of oxidative efficiency. Assembly factors such as NDUFAF6 are non‐enzymatic and have traditionally been regarded as undruggable, yet our findings demonstrate that selective stabilization of an assembly factor is sufficient to reprogram respiratory chain architecture, enhance mitochondrial efficiency, and potentiate thermogenic responsiveness under energetic stress [27]. This paradigm extends mitochondrial pharmacology beyond direct modulation of electron transport or uncoupling and suggests that targeting proteostatic control points may represent a generalizable strategy to optimize mitochondrial performance in metabolically challenged tissues.

At the same time, the present study is intentionally positioned as a mechanistic investigation rather than a preclinical drug‐development effort. Although agnuside exhibited favorable tolerability in vivo, as evidenced by unaltered serum chemistry and the absence of overt histopathological abnormalities following repeated intraperitoneal administration, comprehensive pharmacokinetic profiling, oral bioavailability assessment, and long‐term toxicology studies will be required to evaluate its translational potential. Importantly, these considerations do not detract from the central conclusion of this work: that pharmacological stabilization of mitochondrial assembly machinery can serve as a decisive upstream lever for controlling respiratory chain organization and thermogenic competence. Future efforts aimed at identifying regulatory enzymes governing NDUFAF6 turnover and developing more optimized chemical modulators may further extend the therapeutic relevance of this assembly‐factor‐centric strategy.

## 4 Experimental Section

### 4.1 Animal Studies

All animal experimental protocols were approved by the Ethics Committee of Zhejiang University of Technology (approval number: ZH20231029030). Five‐week‐old C57BL/6J mice were obtained from Shanghai SLAC Laboratory Animal Co., Ltd. (Shanghai, China) and maintained in a controlled environment under a 12‐hour light/dark cycle with free access to a standard chow diet (P1101F, Shanghai SLAC Animal Inc.) and water. Six‐week‐old mice were fed a high‐fat diet (D12492, Research Diet).

Agnuside (AGN) was first dissolved in DMSO and then diluted in a solution containing PEG300, Tween‐80, and physiological saline for intraperitoneal injection. For short‐term studies (4‐week treatment), mice received 10 mg/kg/day, whereas for long‐term metabolic experiments (8‐ or 12‐week treatment), AGN was administered at 7.5 mg/kg/day. Control groups received an equivalent volume of the vehicle solution containing DMSO. All treatments were performed once daily.

For food intake measurement, mice were individually housed with free access to food and water. Pre‐weighed food was provided, and the remaining food was weighed at the indicated time points. Food intake was calculated after correction for spillage and converted to energy intake based on the caloric density of the diet. Data were expressed as kcal per mouse per week.

For indirect calorimetry measurements, mice were individually housed in a comprehensive lab animal monitoring system (PhenoMaster, TSE Instruments) and acclimated for 24 h. Subsequently, oxygen consumption (VO_2_), carbon dioxide production (VCO_2_), heat generation, and respiratory exchange ratio (RER) were continuously monitored over the next 24 h. Data were expressed as averages over the 12‐hour dark and light periods.

All mice were euthanized via carbon dioxide inhalation. Serum insulin levels were determined using an ELISA kit (10‐1113‐01, Mercodia). Serum triglyceride (TG), high‐density lipoprotein cholesterol (HDL‐C), total cholesterol (TC), and low‐density lipoprotein cholesterol (LDL‐C) concentrations were measured using an automated chemistry analyzer (Roche). Serum free fatty acid levels were quantified using a commercial assay kit (A042‐2‐1, Nanjing Jiancheng).

### 4.2 GTT and ITT

GTT and ITT were performed after 16 and 4 h fasting, respectively. Mice received intraperitoneal injections of *D*‐glucose (2 g/kg for the 16‐week HFD cohort; 1 g/kg for the 8‐week thermoneutral HFD and 12‐week room‐temperature HFD cohorts) or insulin (0.75 U/kg for the 16‐week HFD cohort; 0.5 U/kg for the 8‐week thermoneutral HFD and 12‐week room‐temperature HFD cohorts). Blood glucose levels were measured from tail vein samples at the indicated time points.

### 4.3 Cold Tolerance Test

For cold exposure experiments, mice were transferred to pre‐chilled cages maintained at 4°C with water provided ad libitum but with food removed. Rectal temperature was measured at designated time points following cold exposure using an electronic animal thermometer (Alcott Biotech, Shanghai). Dorsal surface temperature was evaluated using an infrared digital thermography camera (T530, Teledyne FLIR) after 5 h. Thermographic images were analyzed with FLIR Tools software to ensure standardized temperature normalization.

### 4.4 Fecal Lipid Measurement

Fecal lipid content was determined using a modified Folch extraction method. Dried fecal samples were collected from individually housed mice over the indicated period and weighed. For each sample, 1000 mg of powdered feces was homogenized in normal saline, followed by extraction with chloroform–methanol (2:1, v/v). After centrifugation, the lower organic phase containing lipids was collected and evaporated to dryness. The extracted lipid residue was weighed to determine total fecal lipid content. Fecal lipid excretion was expressed as the percentage of lipid mass relative to the total fecal dry weight.

### 4.5 Oral Fat Tolerance Test

For the oral fat tolerance test, mice were fasted for 12 h with free access to water. Tyloxapol (MCE, HY‐B1068) was administered by intraperitoneal injection at a dose of 500 mg/kg. After 30 min, baseline (0 h) blood samples were collected, followed by oral gavage of corn oil (MCE, HY‐Y1888) at 10 ml/kg body weight. Additional blood samples were collected at 0.5, 1, 2, 3, and 4 h after gavage. Serum triglyceride levels were measured using a commercial enzymatic assay kit (Nanjing Jiancheng, A110‐1‐1) according to the manufacturer's instructions.

### 4.6 Histological Analysis and Immunohistochemical Staining

Fresh mouse tissues were fixed in 10% neutral‐buffered formalin for at least 24 h, embedded in paraffin, and sectioned at 5–10 µm thickness. For hematoxylin and eosin (H&E) staining, sections of BAT, iWAT, eWAT, and liver were deparaffinized, rehydrated, and stained with hematoxylin and eosin following standard protocols. After dehydration and mounting. For immunohistochemical analysis of UCP1, paraffin‐embedded sections were deparaffinized, rehydrated, and subjected to antigen retrieval in citrate buffer. After blocking with 5% BSA, sections were incubated overnight at 4°C with primary UCP1 antibodies (UCP11‐A, Alpha Diagnostic; 23673‐1‐AP, Proteintech), followed by incubation with HRP‐conjugated secondary antibodies for 1 h at room temperature. UCP1 was visualized using a DAB substrate, followed by dehydration and mounting. All stained sections were examined under a light microscope. Histological quantification of lipid droplet size was performed using Fiji software. At least five random fields per section and three sections per animal were analyzed.

### 4.7 Transmission Electron Microscopy Analysis

Fresh BAT tissues were carefully dissected to minimize mechanical damage and immediately fixed in 2.5% glutaraldehyde prepared in 0.1 m phosphate buffer (pH 7–7.5) at 4°C for 2–4 h. Following fixation, samples were washed three times with 0.1 m phosphate buffer (pH 7.4). Post‐fixation was performed in 1% osmium tetroxide (150910, EMS) in 0.1 m phosphate buffer at room temperature for 2 h, followed by additional washes in buffer. Tissues were dehydrated through a graded ethanol and acetone series, then infiltrated with a mixture of acetone and resin (1210819, SPI), and finally embedded in pure resin. Polymerization was conducted at 60°C for 48 h. Ultrathin sections (60–80 nm) were cut, stained with uranyl acetate and lead citrate, and examined using a transmission electron microscope (HT7650, HITACHI) for image acquisition and analysis.

### 4.8 Quantification of Mitochondrial Diameter and Cristae Abundance

Mitochondrial diameter was determined by measuring the transverse diameter of individual mitochondria in TEM images using ImageJ software. Cristae abundance was evaluated as the ratio of the inner to outer mitochondrial membrane perimeter using ImageJ.

### 4.9 Isolation of SVF Cells From Brown and Inguinal White Adipose Tissues

For brown adipocytes, interscapular BAT was harvested from newborn C57BL/6J mice and digested for 30 min at 37°C in isolation buffer containing 123 mm NaCl, 5 mm KCl, 1.3 mm CaCl_2_, 5 mm glucose, and 100 mm HEPES (pH 7.4), supplemented with 4% bovine serum albumin and 1 mg/mL collagenase B. The digested suspension was filtered through a 40 µm cell strainer and centrifuged at 200 × *g* for 5 min to collect SVF cells, which were cultured in DMEM supplemented with 20% fetal bovine serum until confluence.

For inguinal white adipocytes, iWAT depots were isolated from two‐week‐old C57BL/6J mice and digested for 40–45 min at 37°C in enzyme buffer containing dispase II (2.4 U/mL), collagenase B (1.5 U/mL), and CaCl_2_ (10 mm) in DMEM. The digested suspension was filtered through a 70 µm cell strainer and centrifuged at 700 × *g* for 5 min to isolate SVF cells, which were cultured in DMEM supplemented with 20% fetal bovine serum until confluence.

### 4.10 Generation of Immortalized Brown Preadipocytes

SVF cells derived from neonatal BAT were used to generate immortalized brown preadipocytes. Upon reaching approximately 80% confluence, cells were infected with a neomycin‐resistant retroviral vector pBabe encoding the SV40 large T antigen, kindly provided by Prof. Dongning Pan (Fudan University, Shanghai, China). Following infection, cells were subjected to neomycin (G418) selection for at least one week and further purified by single‐cell cloning. Immortalized clones were expanded and subsequently induced to differentiate into mature brown adipocytes.

### 4.11 Differentiation of Primary Brown and Inguinal White Adipocytes

For primary adipocyte differentiation, confluent SVF cells were induced on Day 0 with lineage‐specific induction media. Brown adipocytes were differentiated in DMEM supplemented with 10% fetal bovine serum, 850 nm insulin, 1 nm triiodothyronine (T3), 0.5 µm dexamethasone, 0.5 mm IBMX, and 0.125 mm indomethacin. Inguinal white adipocytes were differentiated in DMEM/F12 containing 10% fetal bovine serum, 850 nm insulin, 1 nm T3, 0.5 µm dexamethasone, 0.5 mm IBMX, 0.125 mm indomethacin, and 1 µm rosiglitazone.

From Day 2 onward, culture medium was replaced every other day with maintenance medium. For brown adipocytes, maintenance medium consisted of DMEM with 10% fetal bovine serum, insulin (160 nm on Day 2 and 20 nm on Day 4), and 1 nm T3. For white adipocytes, DMEM/F12 supplemented with 10% fetal bovine serum, 850 nm insulin, 1 nm T3, and 1 µm rosiglitazone was used. Differentiation was completed by Day 6.

HEK293T cells and immortalized brown preadipocytes were maintained in DMEM supplemented with 10% fetal bovine serum at 37°C in a humidified atmosphere with 5% CO_2_. Immortalized brown preadipocytes were induced to differentiate upon reaching approximately 70% confluence. Cells were first cultured in DMEM containing 10% fetal bovine serum, 20 nm insulin, and 1 nm triiodothyronine (T3) until Day 0, followed by a 2‐day induction phase using DMEM supplemented with 20 nm insulin, 1 nm T3, 0.5 mm IBMX, 0.5 µm dexamethasone, and 0.125 mm indomethacin. Thereafter, cells were maintained in differentiation medium consisting of DMEM with 10% fetal bovine serum, 20 nm insulin, and 1 nm T3, refreshed every other day until Day 6.

All immortalized brown preadipocytes and primary brown or beige adipocytes were cultured and differentiated according to established protocols as previously described [28, 29].

### 4.12 Compound Library and Dosing Strategy

All 64 iridoid glycosides were obtained as a curated library from MedChemExpress (HY‐L0001) and prepared as 10 mm stock solutions according to the manufacturer's instructions, with most compounds dissolved in DMSO and three in water (Table S1). For primary screening, differentiated brown adipocytes were treated with each compound at a final concentration of 10 µm (1:1000 dilution), with matched vehicle controls (0.1% DMSO or water). This single‐dose design was used for hit identification rather than potency ranking.

### 4.13 Oil‐Red O Staining

Oil‐red O staining was performed using an Oil Red O staining Kit (C0158, Beyotime) according to the manufacturer's instructions. The Oil Red O‐positive area was quantified using Fiji software.

### 4.14 Mitochondrial Membrane Potential and Relative mtDNA Level

Mitochondrial membrane potential was assessed using the MitoProbe JC‐1 Assay Kit (C2006, Beyotime) according to the manufacturer's instructions. Total DNA was extracted from adipose tissues with the FastPure Cell/Tissue DNA Isolation Kit (DC102, Vazyme). Relative mitochondrial DNA (mtDNA) and nuclear DNA content were quantified by qPCR, using *β‐Actin* as the nuclear reference gene and *Cox2* as the mitochondrial reference gene. Primer sequences are provided in Table S4.

### 4.15 Microscale Thermophoresis Assay

Full‐length mouse *Ndufaf6* lacking the mitochondrial targeting sequence was cloned into the pET‐28a expression vector and transformed into *Escherichia coli* BL21(DE3). Protein expression was induced with isopropyl β‐D‐thiogalactopyranoside (IPTG). The recombinant His‐tagged NDUFAF6 protein was purified using Ni‐NTA agarose beads (SA004005, Smart Life Sciences) and eluted with imidazole. The eluate was concentrated and desalted using a 10 kDa molecular weight cutoff centrifugal filter (Merk, UFC9010) at 500 g for 30 min, followed by three washes with phosphate‐buffered saline (PBS). The purity and integrity of the purified protein were verified by SDS‐PAGE followed by Coomassie blue staining.

The purified His‐NDUFAF6 protein was labeled using the RED‐NHS 2nd Generation Protein Labeling Kit (MO‐L011, NanoTemper). For binding analysis, 5 µm labeled His‐NDUFAF6 was incubated with serial dilutions of agnuside (B20879, Shanghai Yuanye Bio‐Technology Co., Ltd), and the mixtures were loaded into capillaries (MO‐K022, NanoTemper). Microscale thermophoresis (MST) measurements were performed using the Monolith X instrument (NanoTemper, Germany). *F*_norm_ = *F*_1_/*F*_0_ (*F*_norm_: normalized fluorescence; *F*_1_: fluorescence after thermodiffusion; *F*_0_: initial fluorescence or fluorescence after T‐jump). The dissociation constant (*K*_d_) was determined using Prism software.

### 4.16 Synthesis of Biotin‐Conjugated Agnuside

Biotinylated agnuside was custom‐synthesized by Shanghai Nafu Biotechnology Co., Ltd. (Shanghai, China). The synthesis and biotin conjugation were carried out according to the manufacturer's standard protocols. The purity and structural integrity of the biotinylated compound were verified by high‐performance liquid chromatography and mass spectrometry, as provided by the supplier. Mass spectrometry results are presented in Supporting Table S2.

### 4.17 Biotinylated Agnuside Pull‐Down

For pull‐down assays, 100 µL of 2 mm biotinylated agnuside or biotin was incubated with 50 µL streptavidin‐agarose beads for 1.5 h at room temperature. Subsequently, lysates from mature adipocytes were added, and the mixtures were incubated for an additional 3 h at room temperature with gentle rotation. After incubation, the beads were collected by centrifugation and washed three times with PBST buffer (PBS, pH 7.4, 0.05% Tween‐20, 0.05% BSA). Two samples were eluted and subjected to mass spectrometry analysis, while the remaining three were resuspended in SDS loading buffer for western blot analysis.

### 4.18 Molecular Docking

The three‐dimensional structure of mouse NDUFAF6 (AlphaFold ID: AF‐A2AIL4‐F1) was retrieved from the AlphaFold database and prepared using the Protein Preparation Wizard, including hydrogen addition and energy minimization with the OPLS2005 force field. The primary druggable binding pocket was identified with the SiteMap module, and the docking grid was generated (box size: 20 × 20 × 20 Å) centered on this site. The structure of agnuside (CAS: 11027‐63‐7) was processed using LigPrep to generate energy‐minimized 3D conformers. Molecular docking was performed with the Glide XP module, and the top‐ranked binding pose was selected for further analysis. Protein–ligand interactions were visualized with PyMOL.

### 4.19 Molecular Dynamics Simulation

Molecular dynamics simulations were performed using GROMACS v2022.03 with the AMBER99SB‐ILDN force field to investigate the stability and dynamic properties of the NDUFAF6‐agnuside complex. The docked complex was solvated in a TIP3P water box with a minimum distance of 1.2 nm from the protein to the box edge, and neutralized with 0.154 mol/L NaCl. Ligand parameters were generated using AmberTools22 (GAFF) with RESP charges computed in Gaussian 16W. Following energy minimization with the steepest descent algorithm, the system was equilibrated under NVT and NPT conditions for 100 ps each at 300 K and 1 bar. Production MD was carried out for 100 ns in the NPT ensemble with a 2 fs integration step, applying periodic boundary conditions. Short‐range interactions were truncated at 1 nm, and long‐range electrostatics were treated using the PME method; all hydrogen bonds were constrained with LINCS.

Trajectory analyses included RMSD, RMSF, radius of gyration (*R*_g_), solvent‐accessible surface area (SASA), and hydrogen bond quantification. Gibbs free energy landscapes were constructed using GROMACS scripts, and binding free energy was estimated for the final 20 ns using the MM/PBSA approach. Per‐residue energy decomposition identified key residues contributing to binding. Representative conformational snapshots were extracted at 25 ns intervals to assess dynamic changes within the binding site.

### 4.20 CETSA

Mature brown adipocytes were lysed by five cycles of freeze–thaw, after which the lysates were incubated with 100 µm agnuside or vehicle control at room temperature for 1 h. Following incubation, the samples were aliquoted and subjected to a temperature gradient (37°C–67°C) for 3 min using a thermocycler. The samples were then immediately placed on ice and centrifuged at 20 000 g for 10 min at 4°C to isolate the soluble protein fraction. Supernatants containing equal amounts of protein were resolved by SDS‐PAGE and analyzed by western blotting with an anti‐NDUFAF6 antibody.

### 4.21 DARTS

Cell lysates were incubated with the indicated concentrations of agnuside for 1 hour. Subsequently, pronase (P8811, Sigma) was added at a final concentration of 5 µg/mL, and the mixture was incubated at room temperature for 20 min. The reactions were terminated by the addition of SDS‐PAGE loading buffer, and the samples were subjected to western blot analysis.

### 4.22 OCAR

Oxygen consumption rate (OCR) was measured using the Seahorse XF Analyzer (Agilent Technologies) according to the manufacturer's protocol. Mature adipocytes were seeded into Seahorse XF cell culture microplates at a density of 10 000 cells per well for primary brown or immortalized brown adipocytes, and 20 000 cells per well for primary beige adipocytes. Cells were incubated overnight at 37°C in a humidified atmosphere containing 5% CO_2_. Prior to the assay, cells were washed and incubated in Seahorse XF assay medium supplemented with 10 mm glucose, 1 mm pyruvate, and 2 mm glutamine at 37°C in a non‐CO_2_ incubator for 1 h to equilibrate temperature and pH. OCR was measured under basal conditions and in response to the sequential injection of mitochondrial stress reagents: oligomycin (2 µm), FCCP (1 µm), and a combination of rotenone and antimycin A (0.5 µm).

### 4.23 Plasmids

Full‐length mouse *Ndufaf6* cDNA lacking the mitochondrial targeting sequence was amplified by PCR and cloned into the lentiviral entry vector pENTR1A (Addgene). Lentiviral particles were produced by cotransfecting HEK293T cells with the transfer vector, pMD2.G, and psPAX2 (all from Addgene). Viral supernatants were collected 48 h post‐transfection.

### 4.24 Generation and Administration of Recombinant Adenovirus

A short hairpin RNA (shRNA) targeting *Ndufaf6* and *Ucp1* under the control of the *AdipoQ* promoter was constructed using an AAV9 vector system (OBIO Tech). For in vivo knockdown in brown adipocytes, AAV‐sh*Ndufaf6* or AAV‐sh*Ucp1* was injected into the interscapular and inguinal fat pads of anesthetized mice. A longitudinal incision was made over the interscapular region to expose the adipose tissue, and the viral suspension was administered at 4–5 distinct sites per fat pad, with a total injection volume of 50 µL and a final dose of 1 × 10^11^ viral genomes (vg) per fat pad. The shRNA target sequences were as follows:

shNdufaf6, 5′‐ATGTGAGAGATGTAGTATATG‐3′;

shUcp1, 5’‐GCTTCCAGTACCATTAGGTAT‐3’.

### 4.25 Western Blot Analysis

Protein extracts from tissues or cultured cells were prepared using lysis buffer supplemented with protease inhibitors. Following centrifugation at 16 000 × *g* for 10 min, supernatants were collected, and protein concentrations were measured with the BCA assay (Beyotime, P0011). Equal quantities of protein (30–40 µg) were subjected to SDS‐PAGE and then electroblotted onto PVDF membranes. After blocking, membranes were incubated with primary antibodies overnight at 4°C, followed by one hour of incubation with HRP‐conjugated secondary antibodies (YEASEN) at room temperature. Protein bands were detected using enhanced chemiluminescence. All immunoblots were quantified by densitometry and normalized to the corresponding loading controls. Quantitative data are presented alongside representative images.

### 4.26 Blue Native PAGE

Brown adipose tissue (50 mg; BAT) or 100 mg of inguinal white adipose tissue (iWAT) was harvested. Mitochondria were isolated and purified using a commercial kit (C3606, Beyotime) following the manufacturer's instructions. The mitochondrial fraction was lysed in buffer containing 50 mm NaCl, 50 mm imidazole, 2 mm 6‐aminohexanoic acid, 1 mm EDTA, and 2% digitonin (pH 7), and incubated on ice for 20 min. Lysates were centrifuged at 20 000 g for 20 min at 4°C, and the supernatant was mixed with native sample buffer (P0761, Beyotime). Protein samples were then loaded onto 4%–13% blue native gels and separated at 4°C using BN‐PAGE running buffer in accordance with the manufacturer's protocols (P0765, P0767, P0769, Beyotime). Following electrophoresis, proteins were transferred onto PVDF membranes and analyzed by western blotting with specific antibodies.

### 4.27 Isolation of Mitochondrial and Cytosolic Fractions

Mitochondrial and cytosolic fractions were isolated by differential centrifugation. Brown adipose tissue or differentiated adipocytes were homogenized in ice‐cold buffer and centrifuged at 600–800 g for 10 min to remove nuclei and debris. The supernatant was subsequently centrifuged at 10 000 g for 10–15 min at 4°C, yielding a mitochondrial pellet that was washed twice and used as the mitochondrial fraction. The corresponding 10 000 g supernatant was collected and designated as the cytosolic soluble fraction. As ultracentrifugation (>100 000 g) was not performed, this fraction may retain residual microsomal or vesicular components (e.g., endoplasmic reticulum), and thus represents an operational rather than a strictly purified cytosolic fraction. This limitation was taken into account during data interpretation.

### 4.28 Immunoprecipitation

To examine the effect of agnuside (AGN) on NDUFAF6 ubiquitination, immortalized brown adipocytes were transduced with a lentiviral vector encoding HA‐NDUFAF6 and differentiated to maturity. Cells were treated with MG132 (10 µm, 6 h) prior to lysis in buffer containing 100 mm NaCl, 0.5% Triton X‐100, 5% glycerol, 50 mm Tris‐HCl (pH 7.5), 1 mm PMSF, and a protease inhibitor cocktail. Cytosolic extracts were incubated with anti‐HA beads overnight at 4°C. Beads were washed three times with wash buffer, and the immunoprecipitates were subjected to western blotting. Ubiquitination of NDUFAF6 was detected using an antibody against endogenous ubiquitin.

To further investigate whether AGN influences the interaction of HSP90 with NDUFA10 and NDUFS3 in the context of NDUFAF6, equal amounts of brown adipose tissue (BAT) from mice were harvested after 5 h of cold exposure and subjected to cytosolic fractionation. The resulting supernatants were incubated with an anti‐HSP90 antibody overnight at 4°C, followed by three washes with wash buffer. Immunoprecipitated complexes were subsequently analyzed by western blotting with antibodies against NDUFA10 and NDUFS3.

### 4.29 RNA Extraction and Quantitative Real‐Time PCR (qPCR)

Total RNA was extracted from cells and tissues using the Total RNA Extraction Reagent (R401, Vazyme). Equal amounts of RNA were reverse transcribed with ABScript III RT Master Mix for qPCR with gDNA Remover (RK20429, ABclonal). Quantitative real‐time PCR was performed using the Universal SYBR Green Fast qPCR Mix (RK21203, ABclonal) on a LightCycler 480 Instrument II (Roche), following the manufacturer's protocols. Primer sequences used for qPCR analysis are provided in Supporting Table S4.

### 4.30 Proteomic Analysis

Following cold exposure at 4°C for 5 h, brown adipose tissue samples (15 mg) were collected from mice treated with agnuside (10 mg/kg, i.p., daily for 4 weeks) for proteomic analysis. Proteomic profiling was conducted by Shanghai Applied Protein Technology Co., Ltd.

### 4.31 Determination of Intracellular Agnuside Levels

After differentiation, immortalized brown adipocytes cultured in 15‐cm dishes were treated with 50 µm agnuside for 2, 4, 6, and 8 h. Following treatment, cells were washed twice with ice‐cold PBS, and mitochondria were isolated using a commercial kit (C3601, Beyotime) according to the manufacturer's instructions. The levels of agnuside in whole‐cell lysates and isolated mitochondrial fractions were measured. All quantitative analyses were performed by Beijing Biotech Pack Scientific Co., Ltd.

### 4.32 Antibodies

The following primary antibodies were used: α‐Tubulin (11224‐1‐AP), NDUFA9 (complex I; 20312‐1‐AP), SDHB (complex II; 10620‐1‐AP), UQCRC1 (complex III; 21705‐1‐AP), COX4 (complex IV; 11242‐1‐AP), and ATP5A1 (complex V; 66037‐1‐Ig), HSP90 (60318‐1‐Ig), anti‐Ubiquitin (10201‐2‐AP), and NDUFS3 (15066‐1‐AP) (Proteintech); β‐Actin (AC026) (ABclonal); PPARγ (2443), AKT (46971), and phospho‐AKT (Ser473) (40607) (Cell Signaling Technology); UCP1 (UCP11‐A) (Alpha Diagnostic); NDUFAF6 (STJ198363) (St John's Laboratory); and NDUFA10 (sc‐376357) (Santa Cruz Biotechnology).

Secondary antibodies included anti‐rabbit IgG (RA1008, Vazyme) and anti‐mouse IgG (RA1009, Vazyme), which were used for western blotting following immunoprecipitation.

### 4.33 Statistical Analysis

Statistical analyses were performed using GraphPad Prism v8.0. Densitometric quantification of western blot bands was conducted using ImageJ. Data are presented as mean ± SEM. For comparisons between two groups, an unpaired two‐tailed Student's *t*‐test was applied when normal distribution was assumed; otherwise, the Mann–Whitney nonparametric test was used. For multiple‐group comparisons, one‐way or two‐way ANOVA was performed as appropriate. Energy metabolism parameters, including VO_2_, VCO_2_, and heat production, were analyzed using ANCOVA with lean mass as a covariate. A value of *p* < 0.05 was considered statistically significant. All in vitro experiments were repeated at least three times, and all in vivo experiments were repeated at least two times.

## Author Contributions

Q.Z. designed the project, performed most of the experiments, acquired the data, and wrote the manuscript. L.X. analyzed the data and provided important reagents for the experiments. S.D. and Z.S. helped in animal experiments. Q.W., B.L., and Y.Z. helped with primary adipocytes. Y.G shared important materials and amended the manuscript. All the authors discussed the results and commented on the manuscript.

## Conflicts of Interest

The authors declare no conflicts of interest.

## Supporting information




**Supporting File 1**: advs76095‐sup‐0001‐FigureS1‐S16.doc.

**Supporting File 2**: advs76095‐sup‐0002‐TableS1‐S4.pptx.

## Data Availability

The data that support the findings of this study are available from the corresponding author upon reasonable request.
