# Supplementary material for: Agnuside Stabilizes the Complex I Assembly Factor NDUFAF6 to Reinforce Mitochondrial Efficiency and Thermogenic Responsiveness
Source: Adv Sci (Weinh). 2026 Jun 16:e16501. Online ahead of print. doi: 10.1002/advs.202516501 (PMC13337116; doi:10.1002/advs.202516501)
Supplement: Supplementary file 1 — Supporting File 1: advs76095‐sup‐0001‐FigureS1‐S16.doc. [file ADVS-9999-e16501-s001.doc]

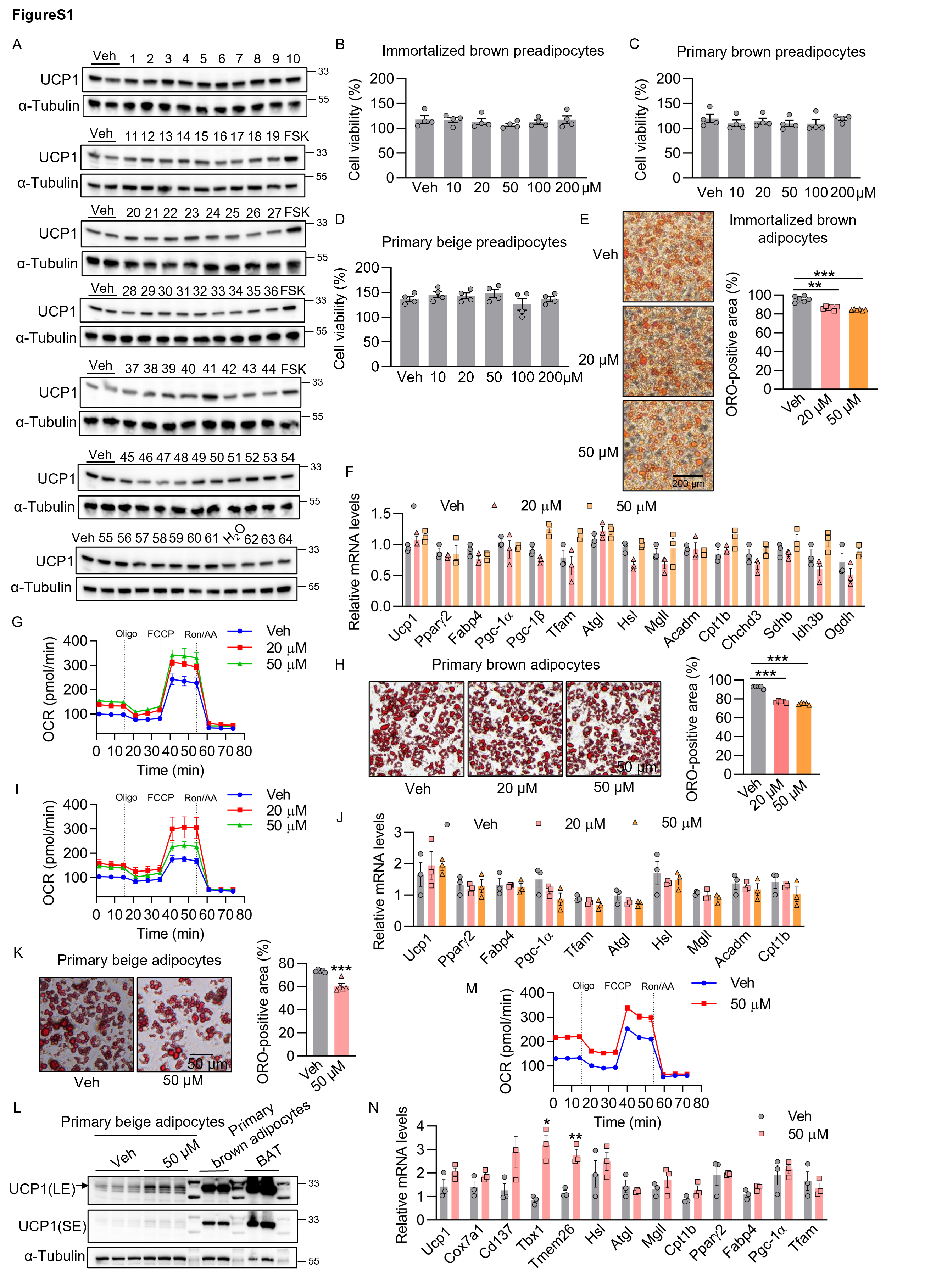


**Figure S1. Screening and functional characterization of agnuside in adipocytes.**

(A) Western blot analysis of UCP1 protein expression in mature brown adipocytes treated with each of the 64 compounds (10 μM) listed in Table S1 for 12 h. Densitometric quantification is shown in Figure 1B.

(B-D) MTT assays of immortalized brown preadipocytes (B), primary brown preadipocytes (C) isolated from neonatal BAT, and primary beige preadipocytes (D) isolated from iWAT of 2-week-old mice. Cells were treated with the indicated concentrations of agnuside (AGN) for 24 h (n = 4 per group).

(E) Representative Oil Red O staining of immortalized brown adipocytes. Cells were differentiated for 6 days, treated with AGN at the indicated concentrations for 12 h, and subjected to Oil Red O staining. Quantification of lipid droplet accumulation is shown on the right (n = 6 per group).

(F) mRNA expression analysis in brown adipocytes treated as in (E) (n = 3 per group).

(G) Oxygen consumption rate (OCR) measurements in brown adipocytes treated as in (E) (n = 6-8 per group).

(H, K) Representative Oil Red O staining of primary brown adipocytes (H) and primary beige adipocytes (K). Primary brown preadipocytes from neonatal BAT and primary beige preadipocytes from iWAT of 2-week-old mice were differentiated into mature adipocytes, treated with AGN at the indicated concentrations for 24 h, and subjected to Oil Red O staining. Quantification of lipid droplet accumulation is shown on the right (n = 5 per group).

(I, M) OCR measurements in primary brown adipocytes (I) and primary beige adipocytes (M) treated as in (H) and (K) (n = 6-8 per group).

(J, N) mRNA expression analysis in primary brown adipocytes (J) and primary beige adipocytes (N) treated as in (H) and (K) (n = 3 per group).

(L) Validation of the authentic UCP1 band. Equal amounts of protein from primary beige adipocytes, primary brown adipocytes, and BAT tissue lysates were loaded together to confirm the UCP1 band position in primary beige adipocytes. SE, short exposure; LE, long exposure.

All experiments were independently repeated three times with consistent results. Data are presented as mean ± SEM of biologically independent samples. Statistical significance was determined by one-way ANOVA (E, H) or two-tailed unpaired Student’s t-test (K, N). **p* < 0.05, ***p* < 0.01, ****p* < 0.001.


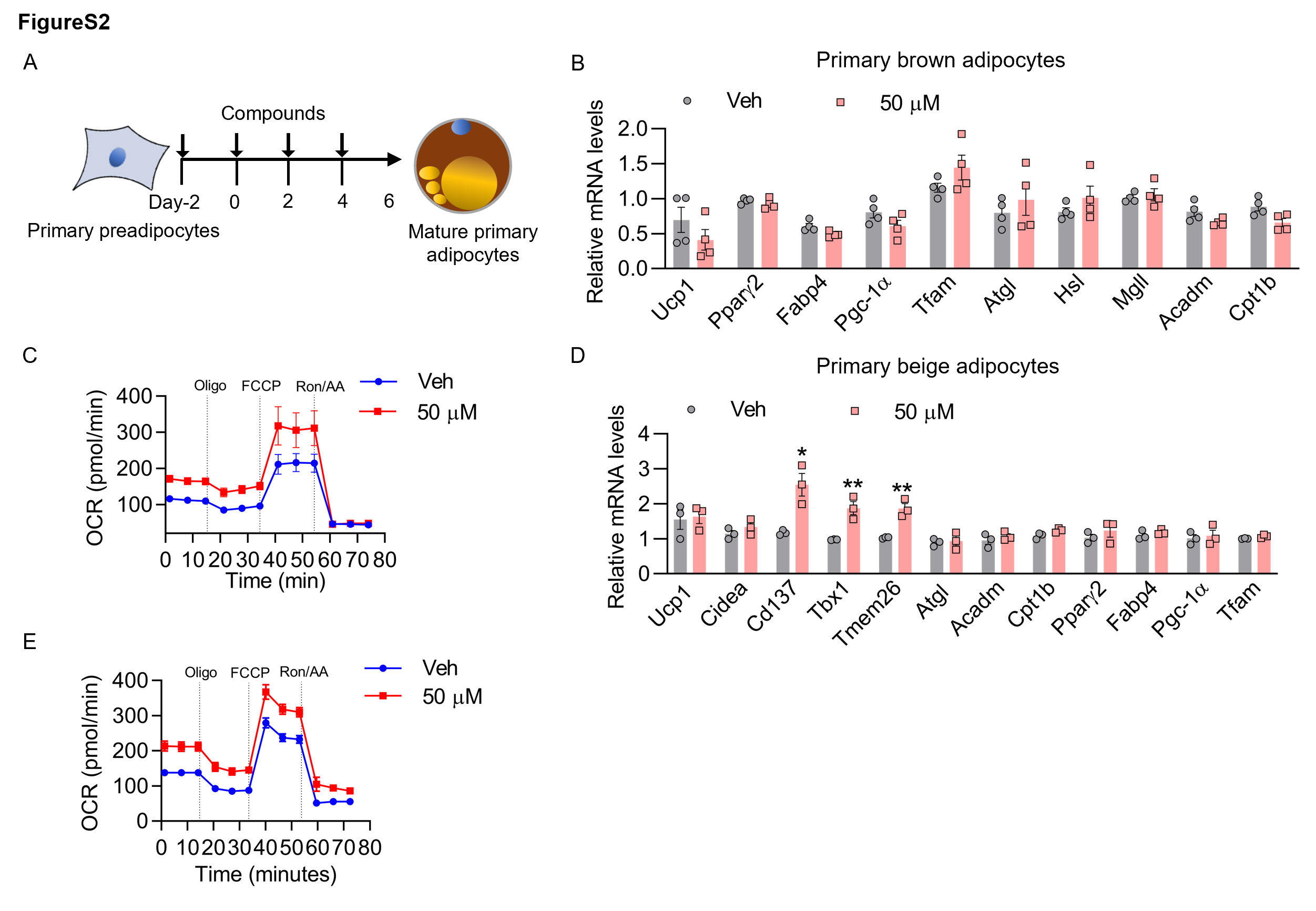


**Figure S2. Continuous agnuside treatment during adipocyte differentiation.**

(A) Schematic representation of the treatment protocol. Primary preadipocytes were exposed to AGN every 2 days from the onset of differentiation until full maturation.

(B, D) mRNA expression analysis in primary brown adipocytes (B) (n = 4 per group) and primary beige adipocytes (D) (n = 3 per group) treated as in (A).

(C, E) OCR in primary brown adipocytes (C) (n = 7-8 per group) and primary beige adipocytes (E) (n = 8 per group) treated as in (B, D).

All experiments were independently repeated three times with consistent results. Data are presented as mean ± SEM of biologically independent samples. Statistical significance was determined by a two-tailed unpaired Student’s t-test (D). **p* < 0.05, ***p* < 0.01.


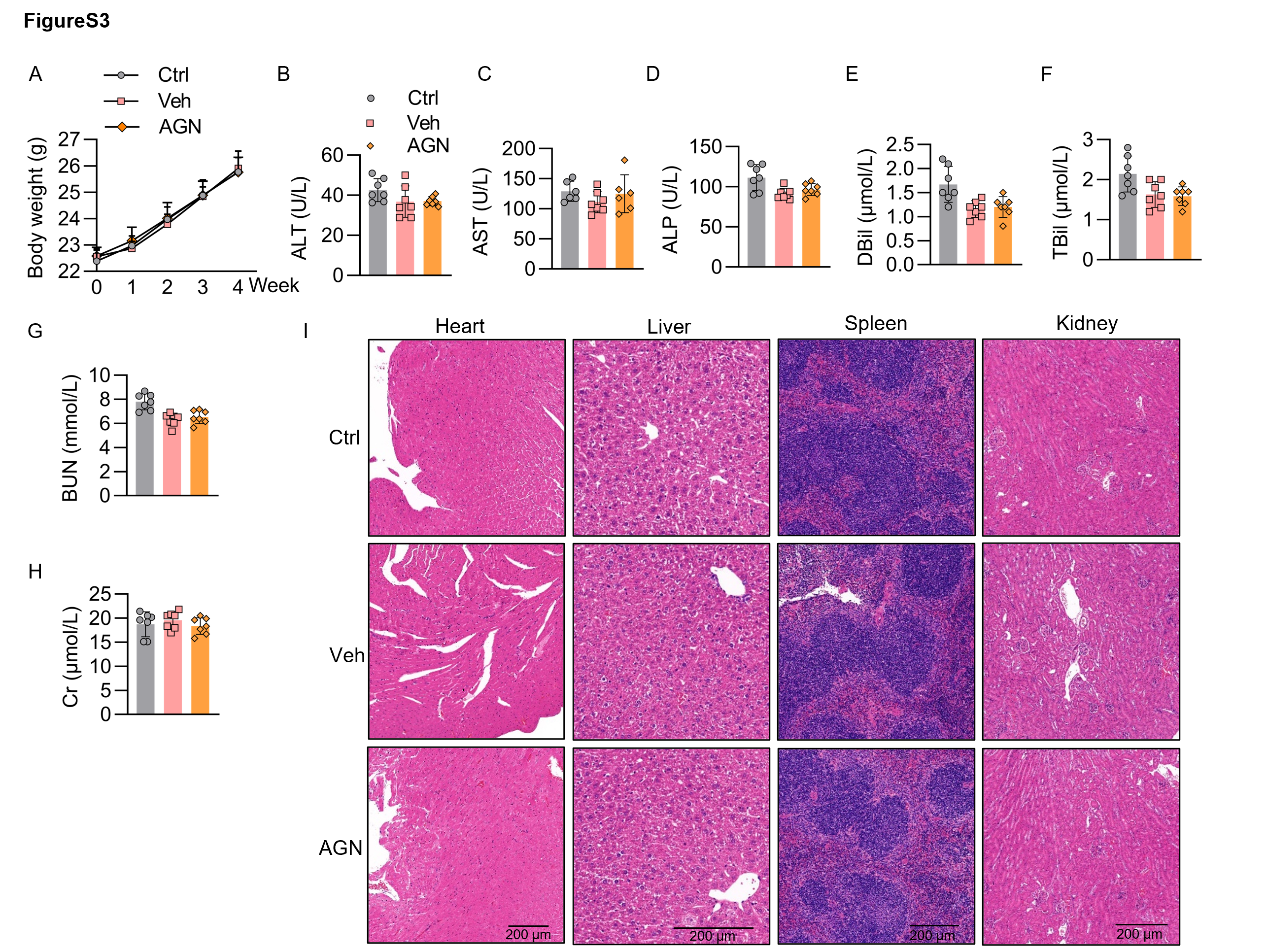


**Figure S3. Short-term agnuside administration does not induce systemic toxicity in mice.**

(A) Body weight trajectories of male C57BL/6J mice. Eight-week-old mice were divided into three groups: untreated control (Ctrl), vehicle-treated (Veh), and agnuside-treated (AGN). Mice in the AGN group received daily intraperitoneal injections of AGN (10 mg/kg), while the vehicle group received an equivalent volume of solvent, for 4 weeks. Body weight was recorded weekly (n = 7 per group).

(B-H) Serum alanine aminotransferase (ALT) (B), aspartate aminotransferase (AST) (C), alkaline phosphatase (ALP) (D), direct bilirubin (DBil) (E), total bilirubin (TBil) (F), blood urea nitrogen (BUN) (G), and creatinine (Cr) (H) levels in mice treated as in (A) (n = 6-7 per group).

(I) Representative H&E staining of heart, liver, spleen, and kidney sections from mice treated as in (A). Scale bar, 200 μm.

All experiments were independently repeated two times with consistent results. Data are presented as mean ± SEM of biologically independent samples.


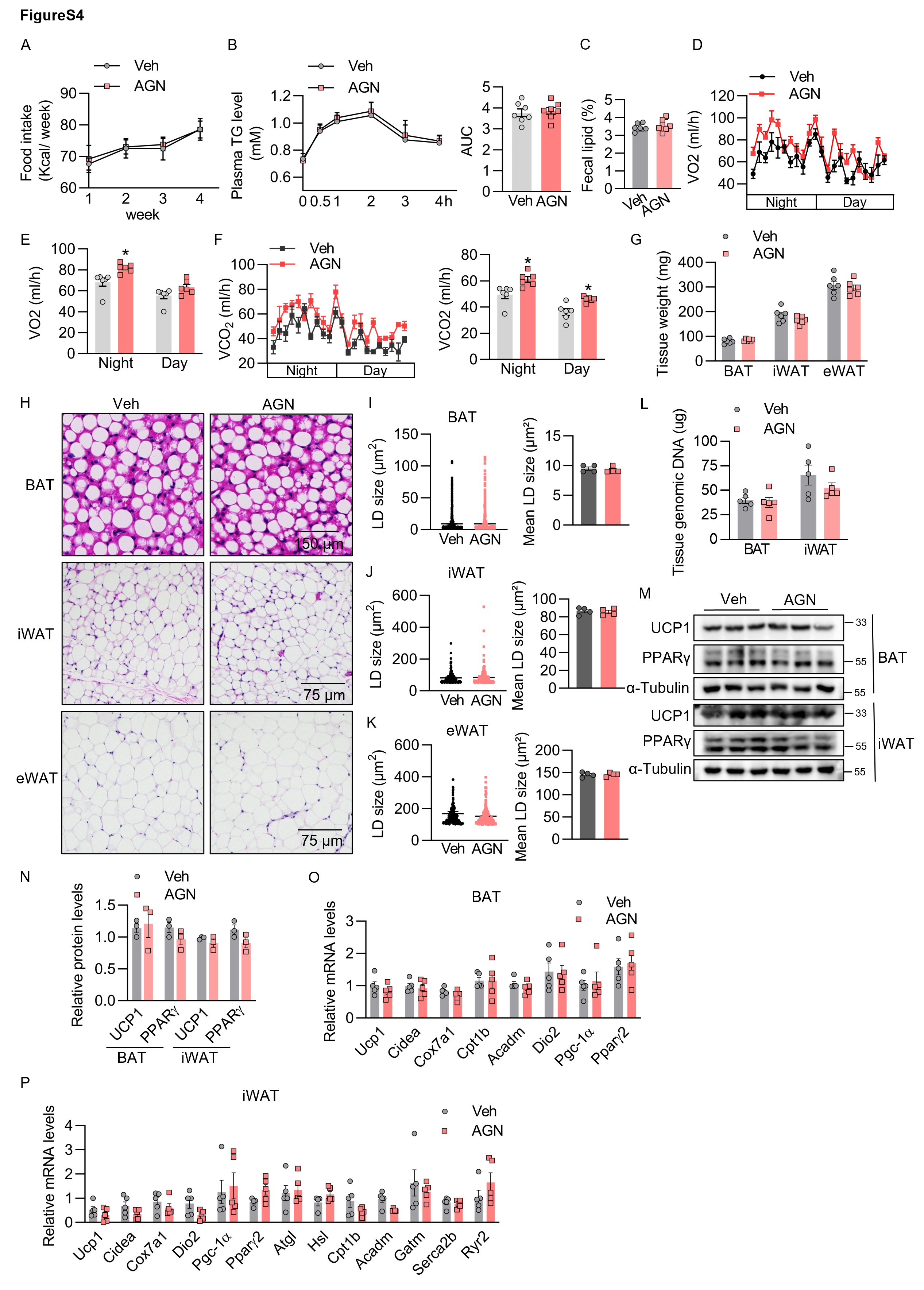


**Figure S4. Agnuside increases energy metabolism without altering food intake, lipid absorption, or adipose tissue morphology.**

(A) Weekly food intake of Veh and AGN-treated (10 mg/kg) mice during the 4-week treatment period (n = 6 per group).

(B) Oral fat tolerance test (OFTT) in mice treated as in (A). After a 12 h fast, mice were injected with tyloxapol. Thirty minutes later, corn oil was administered by oral gavage. Plasma triglyceride (TG) levels were measured at 0, 0.5, 1, 2, 3, and 4 h, and the area under the curve (AUC) is shown on the right (n = 7 per group).

(C) Percentage of fecal lipid content in mice treated as in (A) (n = 6-7 per group).

(D-F) Oxygen consumption (VO₂) trace (D) with quantification (E), and carbon dioxide production (VCO₂) trace (F) with quantification shown on the right (n = 6 per group).

(G) Tissue weights in mice treated as in (A) (n = 6 per group).

(H) Representative H&E staining of BAT, iWAT, and eWAT. Scale bar, 150 μm for BAT, 75 μm for iWAT and eWAT.

(I-K) Lipid droplet size distributions in BAT (I), iWAT (J), and eWAT (K) were quantified from the images in (H). n = 2000 droplets per group in BAT and n = 500 droplets per group in iWAT and eWAT. Pooled individual droplets are shown on the left, and the mean lipid droplet size per mouse is shown on the right (n = 4 per group).

(L) Genomic DNA content in BAT and iWAT from mice treated as in (A) (n = 5 per group).

(M, N) Western blot analysis of protein expression (M) in BAT and iWAT from mice treated as in (A), with densitometric quantification shown in (N) (n = 3 per group).

(O, P) mRNA expression analysis in BAT (O) and iWAT (P) from mice treated as in (A) and subjected to cold exposure (4 °C, 5 h) (n = 5 per group).

All experiments were independently repeated two times with consistent results. Data, except for the left panels in (I-K), are presented as mean ± SEM of biologically independent samples. Statistical significance was determined by ANCOVA with lean mass as a covariate (E, F). **p* < 0.05.


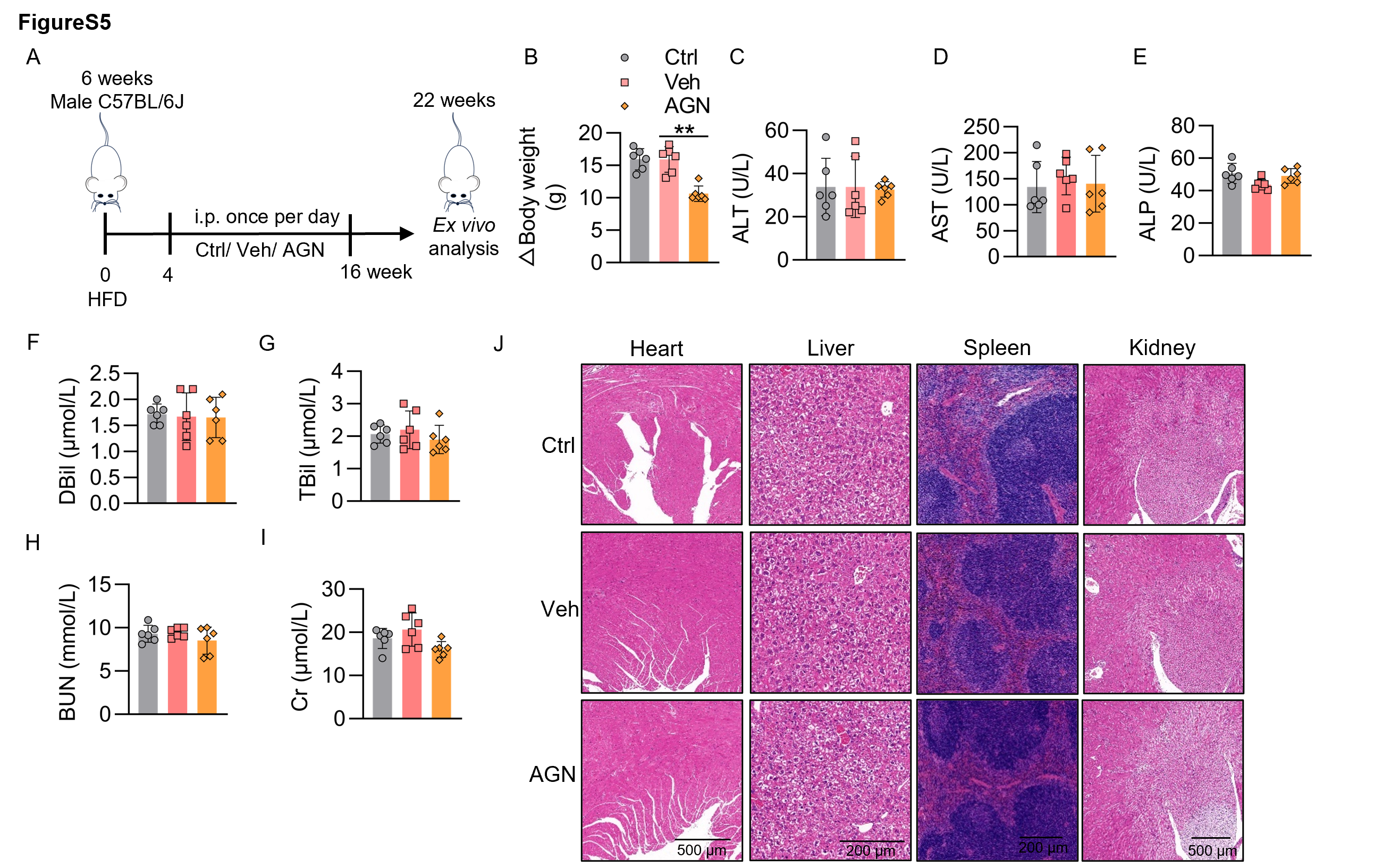


**Figure S5. Long-term agnuside administration does not induce systemic toxicity in mice.**

(A, B) Schematic of the experimental protocol (A) and changes in body weight (Δ body weight) during the treatment period (B) (n = 6 per group). Six-week-old male C57BL/6J mice were fed a high-fat diet (HFD) for 4 weeks and then divided into three groups: Ctrl, Veh, and AGN. Mice in the AGN group received daily intraperitoneal injections of AGN (7.5 mg/kg), while the veh group received an equivalent volume of solvent, for 12 weeks before phenotypic analyses.

(C-I) Serum ALT (C), AST (D), ALP (E), DBil (F), TBil (G), BUN (H), and Cr (I) levels in mice treated as in (A) (n = 6 per group).

(J) Representative H&E staining of heart, liver, spleen, and kidney sections from mice treated as in (A). Scale bars: 500 μm for heart and kidney; 200 μm for liver and spleen.

All experiments were independently repeated two times with consistent results. Data are presented as mean ± SEM of biologically independent samples. Statistical significance was determined by one-way ANOVA (B). ***p* < 0.01.


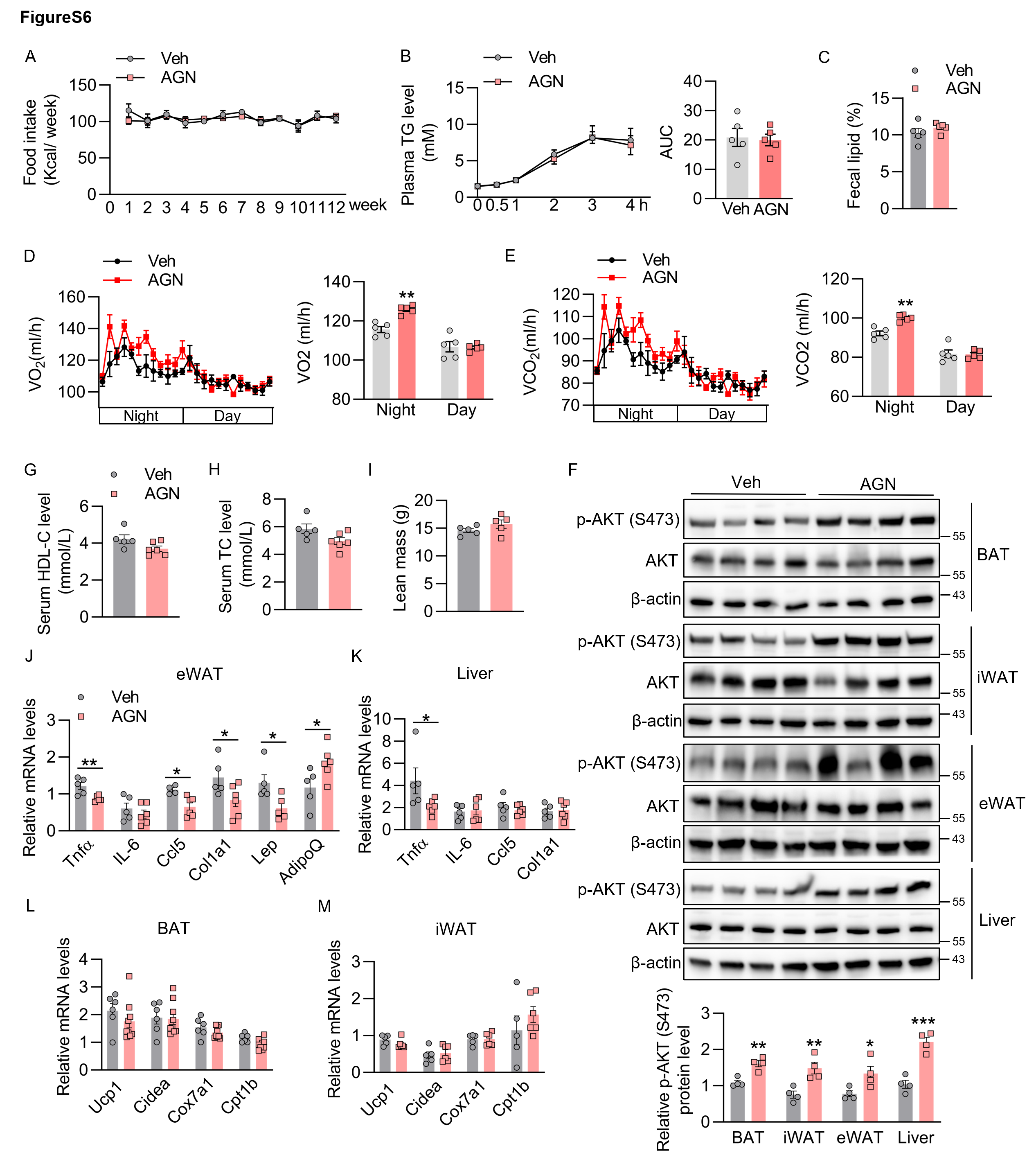


**Figure S6. Agnuside enhances metabolic parameters during high-fat feeding without altering food intake or lipid absorption.**

(A) Weekly food intake of Veh and AGN-treated (7.5 mg/kg, daily i.p.) mice during the 12-week high-fat diet (HFD) treatment period (n = 5 per group).

(B) OFTT in mice treated as in (A). TG levels were measured at 0, 0.5, 1, 2, 3, and 4 h after corn oil gavage, and AUC is shown on the right (n = 5 per group).

(C) Percentage of fecal lipid content in mice treated as in (A) (n = 5 per group).

(D-E) VO_2_ (D) and VCO_2_ (E) in mice treated as in (A), with corresponding quantification (n = 5 per group).

(F) Western blot analysis of protein expression in BAT, iWAT, eWAT, and liver from mice treated as in (A), with densitometric quantification shown below (n = 4 per group).

(G, H) Serum high-density lipoprotein cholesterol (HDL-C) (G) and total cholesterol (TC) (H) levels in mice treated as in (A) (n = 5-6 per group).

(I) Lean mass of mice treated as in (A), measured by body composition analysis (n = 5 per group).

(J-M) mRNA expression analysis in eWAT (J, n = 5-6 per group), liver (K, n = 5-6 per group), BAT (L, n = 6-9 per group), and iWAT (M, n = 5-6 per group) from mice treated as in (A).

All experiments were independently repeated two times with consistent results. Data are presented as mean ± SEM of biologically independent samples. Statistical significance was determined by ANCOVA with lean mass as a covariate (D, E) or two-tailed unpaired Student’s t-test (F, J-K). **p* < 0.05, ***p* < 0.01, ****p* < 0.001.


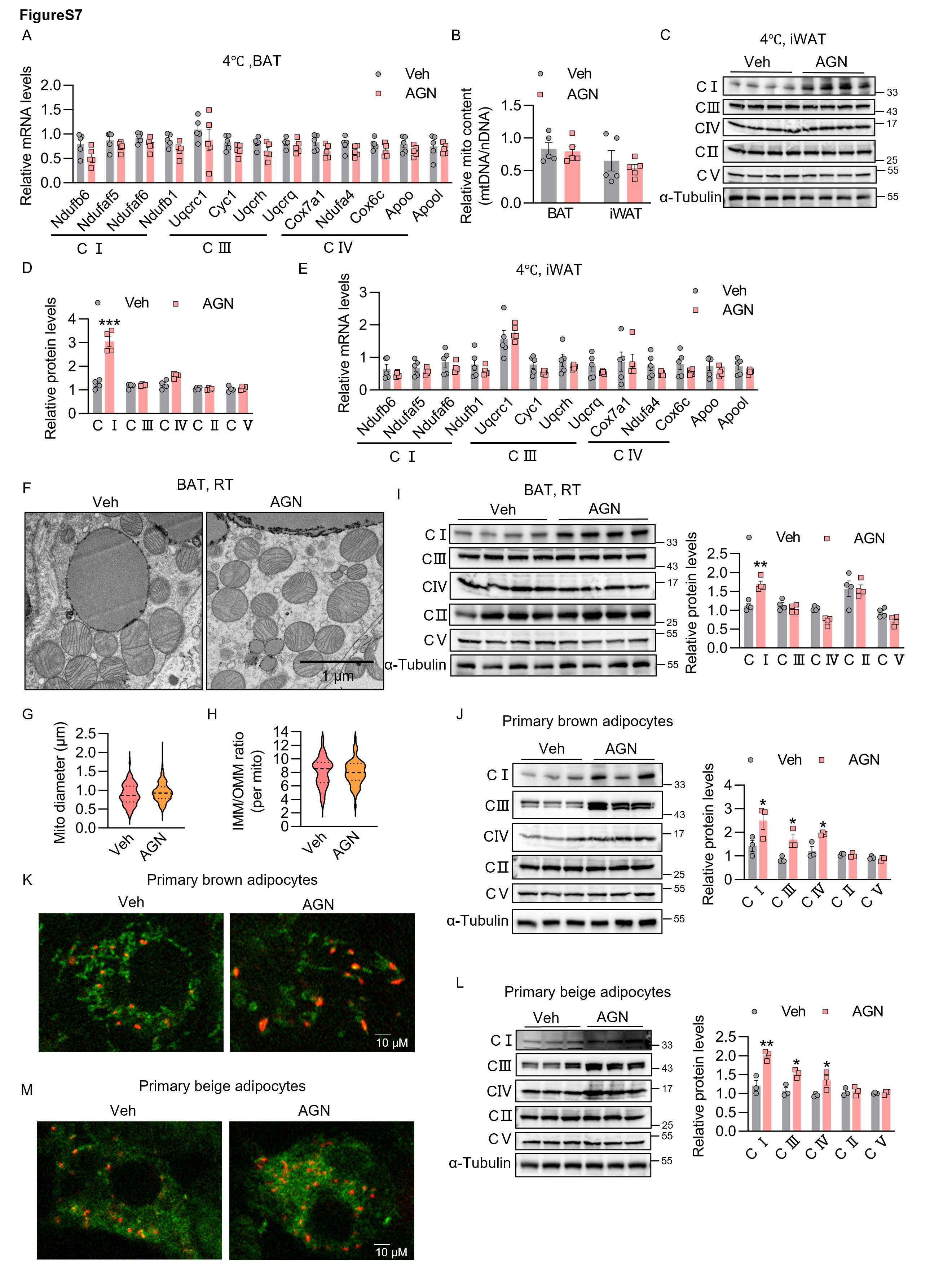


**Figure S7. Demand-dependent and cell-autonomous mitochondrial effects of agnuside in thermogenic adipose tissues.**

(A) mRNA expression analysis in BAT from mice treated with AGN (10 mg/kg, daily i.p.) for 4 weeks, followed by acute cold exposure (4 °C, 5 h) (n = 5 per group). BAT tissue was collected for analysis.

(B) Relative mitochondrial content in BAT and iWAT from mice treated as in (A) (n = 5 per group).

(C-D) Western blot analysis of representative respiratory complex subunits in iWAT from mice treated as in (A), with densitometric quantification shown in (D) (n = 4 per group).

(E) mRNA expression analysis in iWAT of mice treated as in (A) (n = 5 per group).

(F) Representative TEM images of BAT from AGN-treated mice maintained at room temperature.

(G, H) Quantification of mitochondrial diameter (G) and cristae abundance (H) from TEM images shown in (F). n = 300 mitochondria per group in (G); n = 60 mitochondria per group in (H).

(I) Western blot analysis of representative respiratory complex subunits in BAT from AGN-treated mice at room temperature, with densitometric quantification shown on the right (n = 4 per group).

(J, L) Western blot analysis of representative respiratory complex subunits in primary brown adipocytes (J) and primary beige adipocytes (L). Primary brown preadipocytes isolated from neonatal BAT and primary beige preadipocytes isolated from iWAT of 2-week-old mice were differentiated into mature adipocytes and treated with AGN for 24 h. Densitometric quantification is shown on the right (n = 3 per group).

(K, M) Representative assessment of mitochondrial membrane potential by JC-1 staining in mature primary brown adipocytes (K) and primary beige adipocytes (M) treated as in (J, L).

All experiments were independently repeated two times with consistent results. Data, except for panels (G, H), are presented as mean ± SEM of biologically independent samples. Statistical significance was determined by a two-tailed unpaired Student’s t-test (D, I, J, L). **p* < 0.05, ***p* < 0.01, ****p* < 0.001.


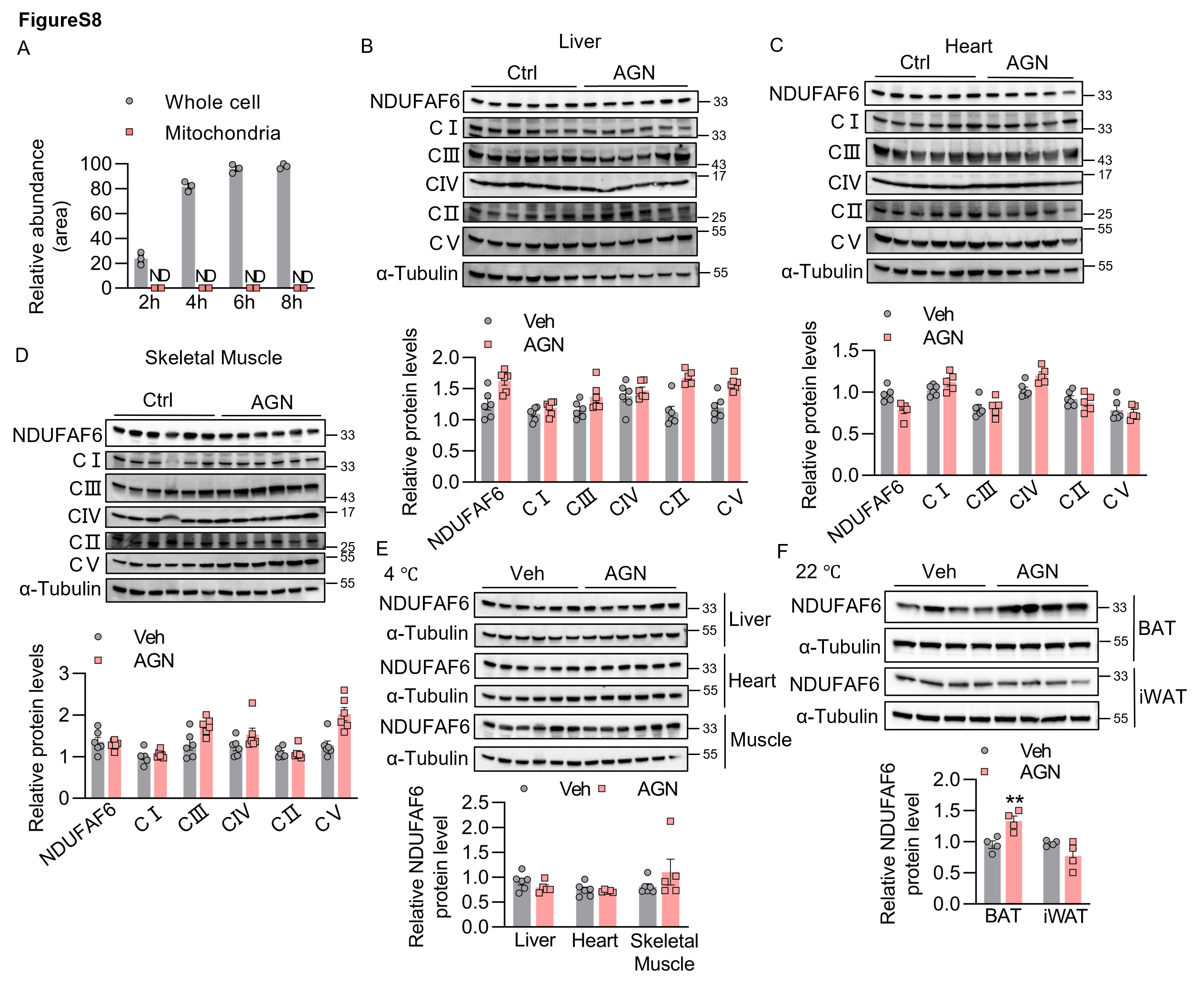


**Figure S8. Agnuside preferentially enhances NDUFAF6 in thermogenic adipose tissues.**

(A) LC-MS/MS analysis of AGN abundance in whole-cell and mitochondrial fractions of mature brown adipocytes (n = 3 per group). Immortalized brown preadipocytes were differentiated into mature adipocytes and treated with 50 μM AGN for 2, 4, 6, or 8 h. Mitochondrial fractions were isolated, and both fractions were analyzed. ND indicates not detected.

(B-D) Western blot analysis in multiple tissues, including liver (B), heart (C), and skeletal muscle (D), from 8-week-old male C57BL/6J mice treated with AGN under HFD feeding conditions for 12 weeks. AGN was administered intraperitoneally at 7.5 mg/kg. Quantification is shown in the bottom panel (n = 5-6 per group).

(E) Western blot analysis of NDUFAF6 protein levels in liver, heart, and skeletal muscle, from 10-week-old male C57BL/6J mice treated with AGN (10 mg/kg, i.p., 4 weeks). Quantification is shown in the bottom panel (n = 6 per group).

(F) Western blot analysis of NDUFAF6 protein levels in BAT and iWAT from 10-week-old male C57BL/6J mice treated with AGN (10 mg/kg, i.p.) for 4 weeks and then exposed to cold at 4 °C for 5 h. Quantification is shown in the bottom panel (n = 4 per group).

All experiments were independently repeated two times with consistent results. Data are presented as mean ± SEM of biologically independent samples. Statistical significance was determined by a two-tailed unpaired Student’s t-test (F). ***p* < 0.01.


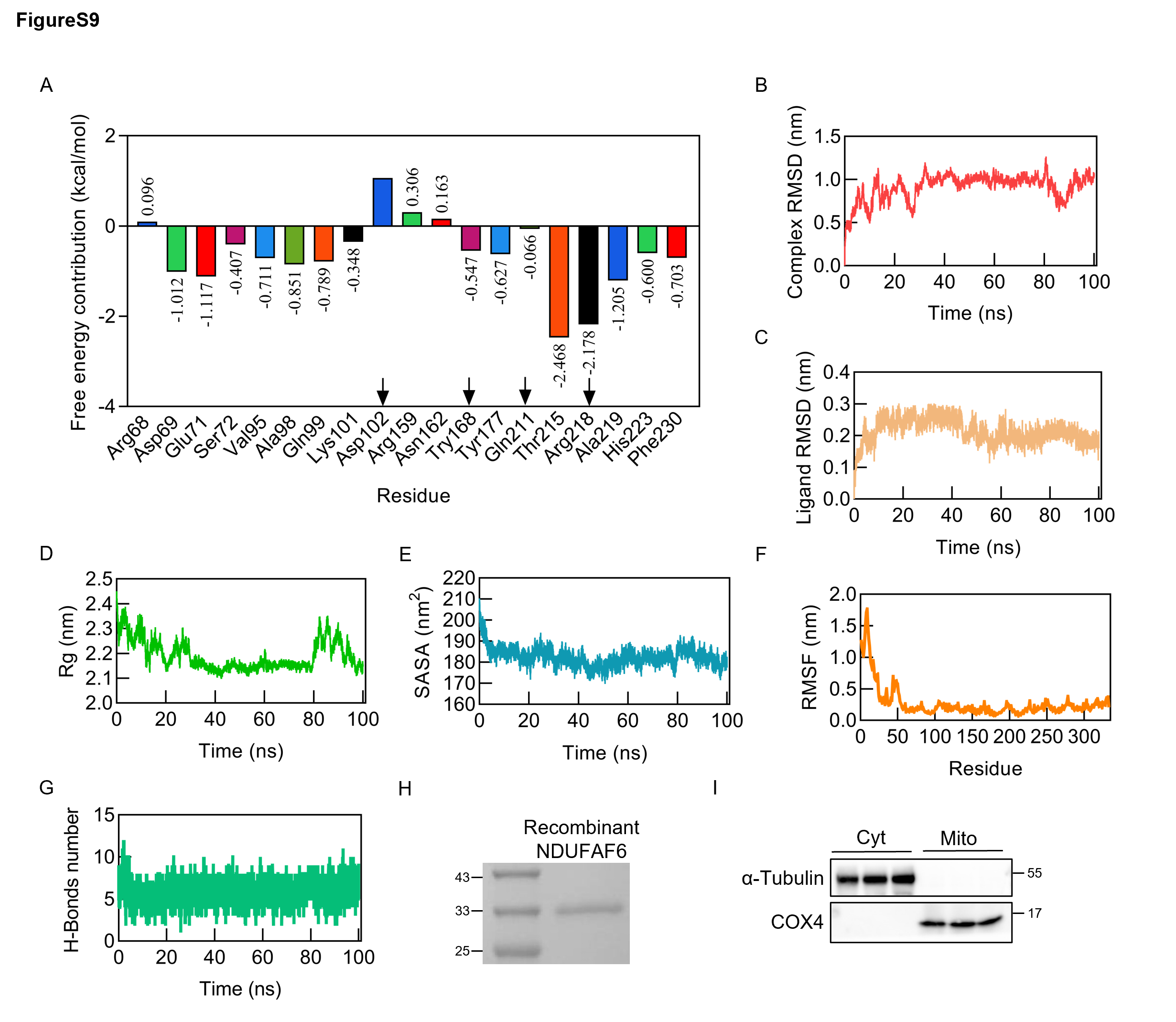


**Figure S9. Molecular dynamics and biochemical validation of the Agnuside-NDUFAF6 interaction.**

(A-G) Molecular dynamics (MD) simulations of the AGN-NDUFAF6 complex, including per-residue energy decomposition (A), complex RMSD (B), ligand RMSD (C), radius of gyration (Rg) (D), solvent-accessible surface area (SASA) (E), RMSF (F), and hydrogen bond (G) analysis.

(H) Coomassie-stained gel of purified NDUFAF6 protein.

(I) Western blot validation of BAT subcellular fractionation. α-Tubulin was used as a cytosolic marker and COX4 as a mitochondrial marker.

All experiments were independently repeated two times with consistent results.


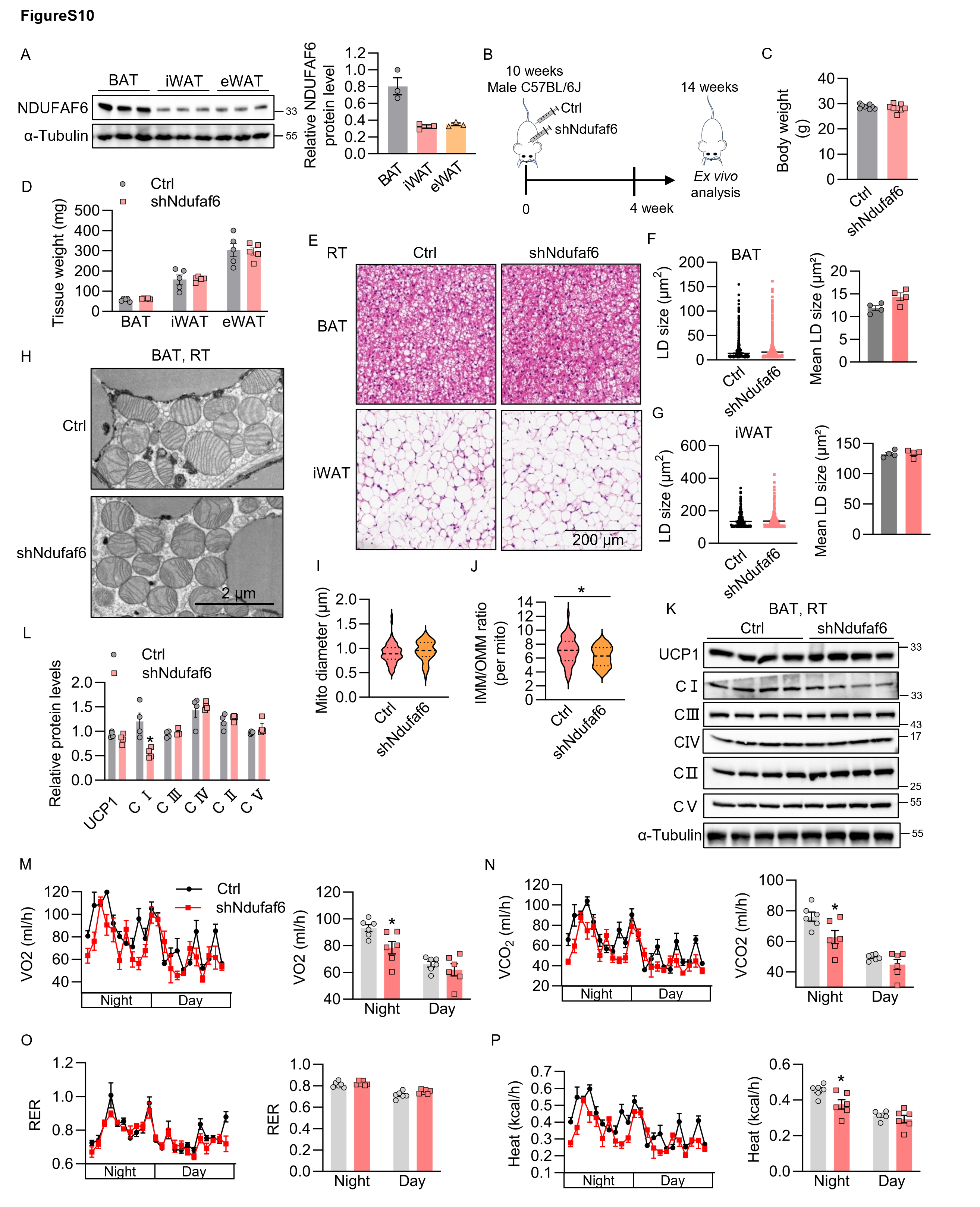


**Figure S10. NDUFAF6 is required for mitochondrial integrity and basal energy metabolism in thermogenic adipose tissue.**

(A) Western blot analysis of NDUFAF6 protein expression in BAT, iWAT, and eWAT, with densitometric quantification shown on the right (n = 3 per group).

(B) Schematic of the experimental protocol. Ten-week-old male mice received AAV-mediated *Ndufaf6* knockdown in BAT and iWAT, followed by 4 weeks of standard chow feeding at room temperature before phenotypic analyses.

(C, D) Body weight (C, n = 8-9 per group) and adipose tissue mass (D, n = 5 per group) of *Ndufaf6* knockdown mice.

(E) Representative H&E staining of adipose tissue from sh*Ndufaf6* mice maintained at room temperature. Scale bar, 200 μm.

(F-G) Lipid droplet size distributions in BAT (F) and iWAT (G) were quantified from the images (E). n = 3000 droplets per group in BAT and n = 500 droplets per group in iWAT. Pooled individual droplets are shown on the left, and the mean lipid droplet size per mouse is shown on the right (n = 4 per group).

(H) Representative TEM images of BAT from sh*Ndufaf6* mice at room temperature.

(I, J) Quantification of mitochondrial diameter (I) and cristae abundance (J) from TEM images shown in (H). n = 300 mitochondria per group in (I); n = 60 mitochondria per group in (J).

(K, L) Western blot analysis of representative respiratory complex subunits in BAT (K) from sh*Ndufaf6* mice at room temperature, with densitometric quantification shown in (L) (n = 4 per group).

(M-P) VO_2_ (M), VCO_2_ (N), respiratory exchange ratio (RER) (O), and heat production (P) in sh*Ndufaf6* mice, with corresponding quantification (n = 6 per group).

All experiments were independently repeated two times with consistent results. Data are presented as mean ± SEM of biologically independent samples. Statistical significance was determined by two-tailed unpaired Student’s t-test (J, L), or ANCOVA with lean mass as a covariate (M, N, P). **p* < 0.05.


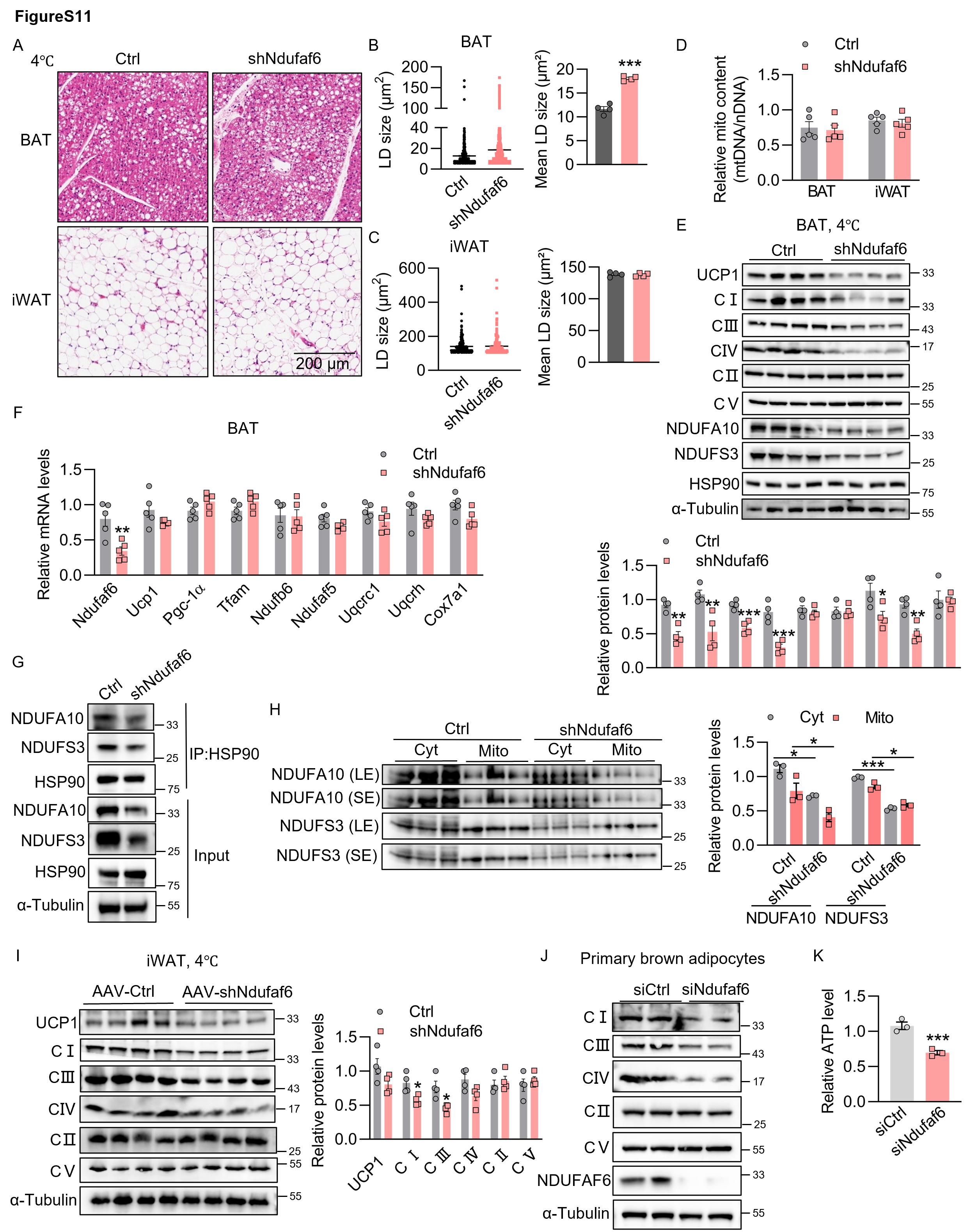


**Figure S11. NDUFAF6 is required for mitochondrial respiratory assembly and ATP production under cold challenge.**

(A) Representative H&E staining of adipose tissue from sh*Ndufaf6* mice at 4℃ for 5 h. Scale bar, 200 μm.

(B-C) Lipid droplet size distributions in BAT (B) and iWAT (C) were quantified from the images (A). n = 3000 droplets per group in BAT and n = 500 droplets per group in iWAT. Pooled individual droplets are shown on the left, and the mean lipid droplet size per mouse is shown on the right (n = 4 per group).

(D) Relative mitochondrial content in BAT and iWAT from mice treated as in (A) (n = 5 per group).

(E) Western blot analysis in BAT from mice treated as in (A), with densitometric quantification shown below (n = 4 per group).

(F) mRNA expression analysis in BAT from mice treated as in (A) (n = 5 per group).

(G) Co-immunoprecipitation of cytosolic HSP90 with NDUFA10 and NDUFS3 in BAT of mice treated as in (A). Cytosolic fractions were immunoprecipitated with an anti-HSP90 antibody, and associated proteins were detected by western blotting. Note (G, H): The cytosolic fraction was obtained as the 10,000 g supernatant without additional ultracentrifugation, and may therefore include minor microsomal contamination.

(H) Western blot analysis of NDUFA10 and NDUFS3 protein expression in cytosolic and mitochondrial fractions of BAT from sh*Ndufaf6* mice treated as in (A) (n = 3 per group). SE, short exposure; LE, long exposure.

(I) Western blot analysis of representative respiratory complex subunits in iWAT from mice treated as in (A), with densitometric quantification shown on the right (n = 4 per group).

(J) Western blot analysis of representative respiratory complex subunits in primary adipocytes. Primary preadipocytes isolated from neonatal mice were differentiated for 3 days, transfected with siRNA, and harvested for protein analysis on day 6.

(K) Relative ATP levels in primary adipocytes treated as in (J) (n = 3 per group).

All experiments were independently repeated two or three times with consistent results. Data are presented as mean ± SEM of biologically independent samples. Statistical significance was determined by a two-tailed Mann-Whitney test (B), two-tailed unpaired Student’s t-test (E, F, I, K), or one-way ANOVA (H). **p* < 0.05, ***p* < 0.01, ****p* < 0.001.


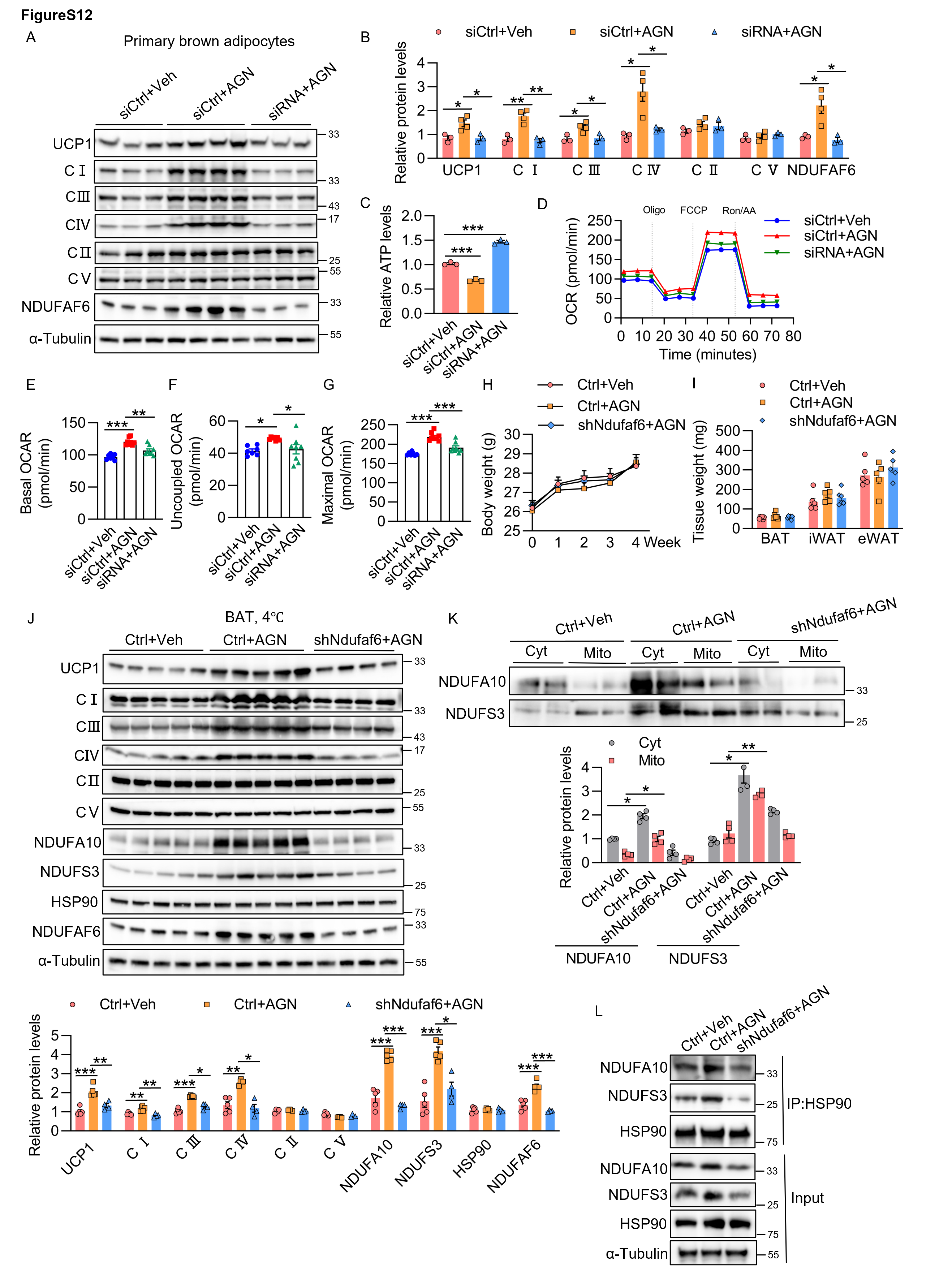


**Figure S12. NDUFAF6 is required for agnuside-induced respiratory complex assembly and mitochondrial function.**

(A-B) Western blot analysis of representative respiratory complex subunits (A) in primary adipocytes. Primary preadipocytes isolated from neonatal mice were differentiated for 3 days, transfected with siRNA, further differentiated to day 6, and then treated with 50 μM AGN for 24 h. Densitometric quantification is shown in (B) (n = 3-4 per group).

(C) Relative ATP levels in primary brown adipocytes treated as in (A) (n = 3 per group).

(D-G) The complete OCR trace (D) in primary brown adipocytes treated as in (A) (n = 7-8 per group). Basal (E), uncoupled (F), and maximal (G) OCR were quantified (n = 7-8 per group).

(H-I) Body weight (H) (n = 5-9 per group) and adipose tissue mass (I) (n = 5-6 per group) of mice. Ten-week-old male mice received *AdipoQ*-Cre-driven AAV-sh*Ndufaf6* injections into BAT and iWAT depots, followed by a two-week recovery period to ensure effective knockdown. Mice were then treated with AGN (10 mg/kg, i.p.) or vehicle daily for four weeks under continuous normal chow feeding, with body weight monitored weekly.

(J) Western blot analysis in BAT from mice in (H) following 5 h cold challenge (4 °C), with densitometric quantification shown below (n = 4-5 per group).

(K) Western blot analysis of NDUFA10 and NDUFS3 protein expression in cytosolic and mitochondrial fractions of BAT from sh*Ndufaf6* mice treated as in (J) (n = 2 per group). Note (K, L): The cytosolic fraction was obtained as the 10,000 g supernatant without additional ultracentrifugation, and may therefore include minor microsomal contamination.

(L) Co-immunoprecipitation of HSP90 with NDUFA10 and NDUFS3 in cytosolic fractions of BAT from mice treated as in (J). Cytosolic lysates were immunoprecipitated with anti-HSP90 antibody, and precipitated proteins were analyzed by western blotting.

All experiments were independently repeated two or three times with consistent results. Data are presented as mean ± SEM of biologically independent samples. Statistical significance was determined by one-way ANOVA (B, C, E, F, G, J, K). **p* < 0.05, ***p* < 0.01, ****p* < 0.001.


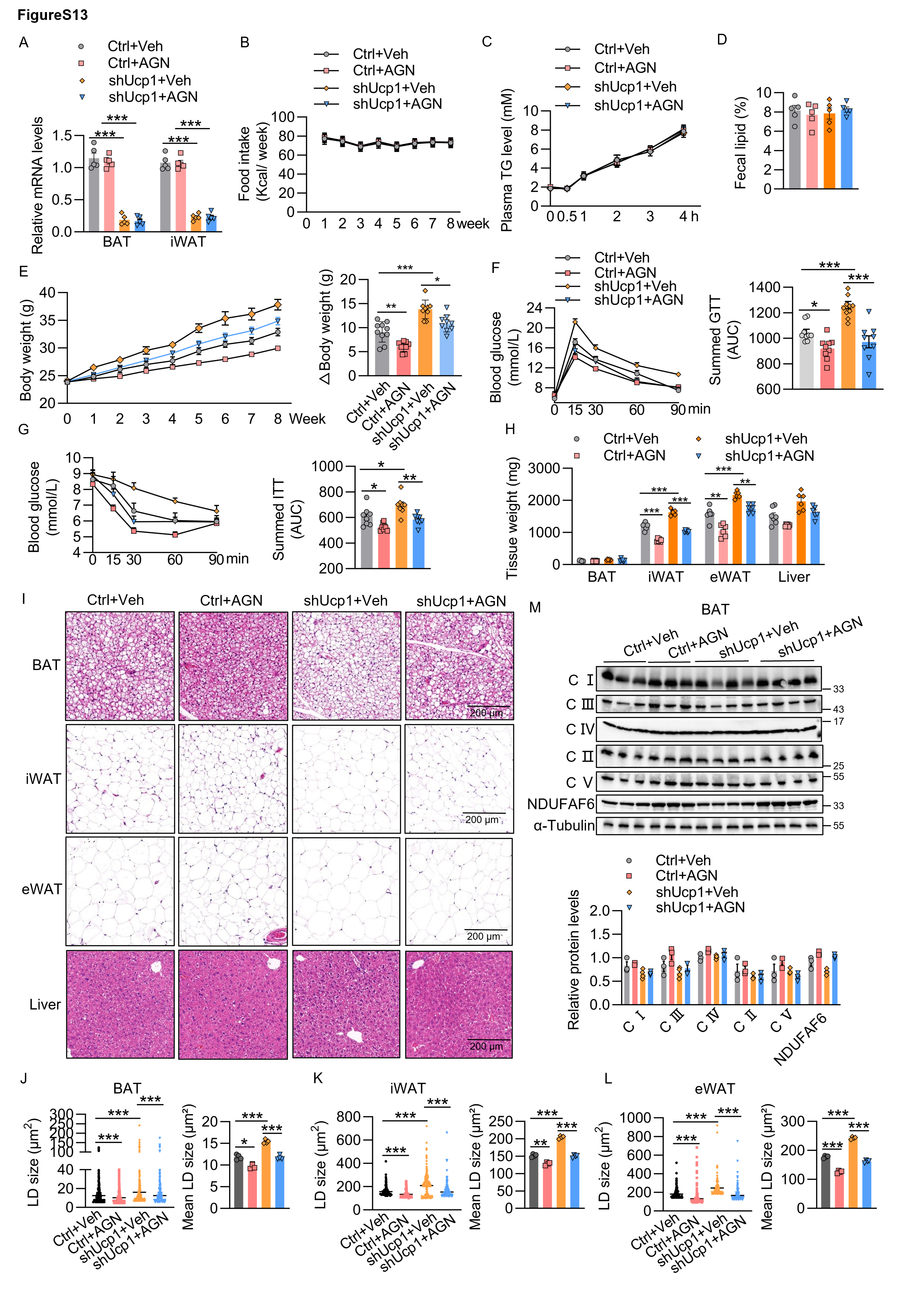


**Figure S13. Agnuside exerts metabolic benefits independently of UCP1 under thermoneutral high-fat feeding.**

(A) *Ucp1* mRNA level in BAT and iWAT (n = 5 per group). Ten-week-old male mice received AAV-mediated *Ucp1* knockdown in BAT and iWAT, followed by 2 weeks of recovery at room temperature. Mice were then maintained at thermoneutrality (30 °C) under HFD feeding with daily intraperitoneal injections of AGN (7.5 mg/kg) or vehicle for 8 weeks.

(B) Weekly food intake during the 8-week high-fat diet (HFD) treatment period described in (A) (n = 5-7 per group).

(C) OFTT in mice treated as in (A). Plasma TG levels were measured at 0, 0.5, 1, 2, 3, and 4 h after corn oil gavage (n = 7 per group).

(D) Percentage of fecal lipid content in mice treated as in (A) (n = 5 per group).

(E) Body weight of mice treated as in (A) (n = 8-10 per group).

(F-G) Glucose tolerance test (GTT) (F) (n = 9-11 per group) and insulin tolerance test (ITT) (G) in mice treated as in (A) (n = 7-9 per group).

(H) Tissue weight of BAT, iWAT, eWAT, and liver from mice treated as in (A) (n = 6-7 per group).

(I) Representative H&E staining of BAT, iWAT, eWAT, and liver from mice treated as (A). Scale bar, 200 μm.

(J-L) Lipid droplet size distributions in BAT (J), iWAT (K), and eWAT (L) were quantified from the images (I). n = 3000 droplets per group in BAT and n = 500 droplets per group in iWAT and eWAT. Pooled individual droplets are shown on the left, and the mean lipid droplet size per mouse is shown on the right (n = 4 per group).

(M) Western blot analysis of representative protein expression in BAT from mice treated as in (A), with densitometric quantification shown below (n = 3-4 per group).

All experiments were independently repeated two times with consistent results. Data are presented as mean ± SEM of biologically independent samples. Statistical significance was determined by one-way ANOVA (A, E, F, G, H, J, K, L). **p* < 0.05, ***p* < 0.01, ****p* < 0.001.


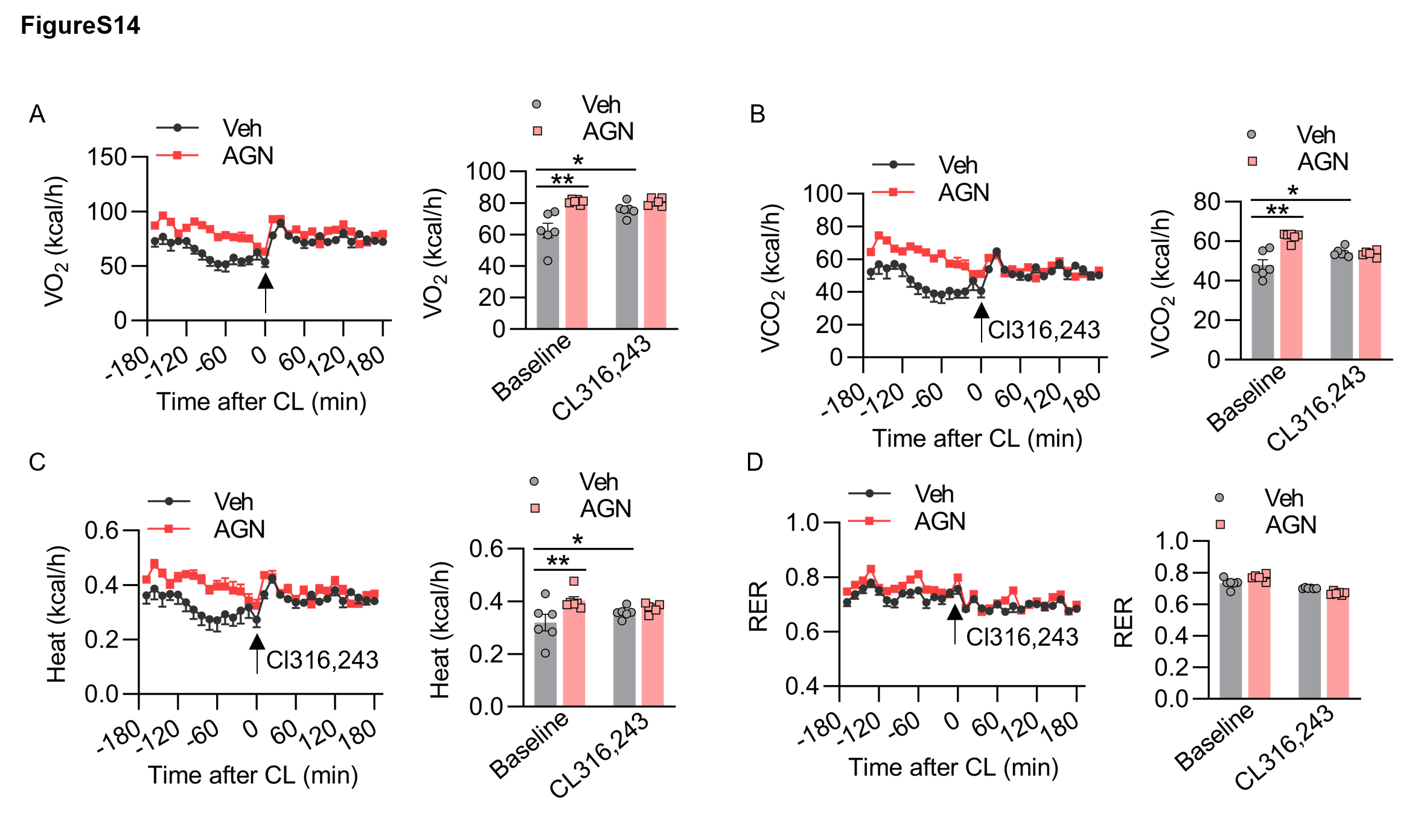


**Figure S14. Metabolic response to β3-Adrenergic stimulation in agnuside-treated mice under thermoneutrality.**

(A-D) Mice were maintained at thermoneutrality (30 °C) under HFD feeding and received daily intraperitoneal injections of AGN (7.5 mg/kg) or vehicle for 8 weeks. To assess β3-adrenergic-stimulated metabolic responses, mice were first monitored for 3 h to establish basal metabolic parameters, followed by intraperitoneal administration of CL316,243 at 1 μg/ g body weight and continued monitoring for an additional 3 h. VO₂ (A), VCO₂ (B), heat production (C), and RER (D) are shown with corresponding quantification (n = 6 per group).

All experiments were independently repeated two times with consistent results. Data are presented as mean ± SEM of biologically independent samples. Statistical significance was determined by ANCOVA with lean mass as a covariate (A, B, C). **p* < 0.05, ***p* < 0.01.


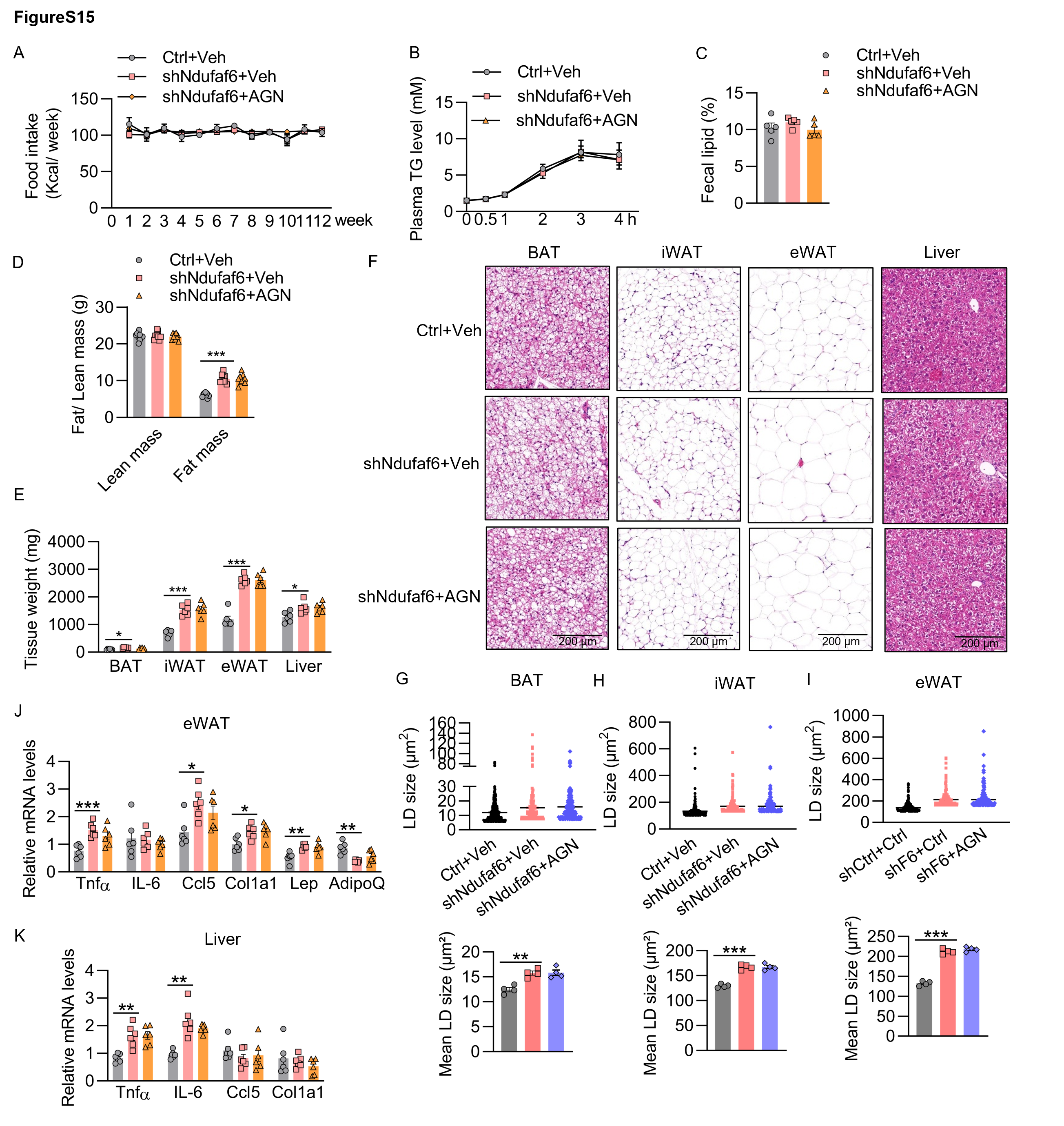


**Figure S15. Metabolic benefits of agnuside are attenuated in *Ndufaf6*-deficient mice during high-fat feeding.**

(A) Weekly food intake during the high-fat diet (HFD) treatment period (n = 5 per group). Eight-week-old mice received local injections of AAV expressing shRNA against *Ndufaf6* under the *AdipoQ* promoter into BAT and iWAT depots. Mice were maintained on standard chow at room temperature for 2 weeks to allow viral expression, followed by 12 weeks of HFD feeding with daily intraperitoneal injections of agnuside (7.5 mg/kg) or vehicle before phenotypic analyses.

(B) OFTT in mice treated as in (A). Plasma TG levels were measured at 0, 0.5, 1, 2, 3, and 4 h after corn oil gavage (n = 5 per group).

(C) Percentage of fecal lipid content in mice treated as in (A) (n = 5 per group).

(D) Fat mass and lean mass of mice treated as in (A) (n = 9 per group).

(E) Tissue weight of BAT, iWAT, eWAT, and liver from mice treated as in (A) (n = 6 per group).

(F) Representative H&E staining of BAT, iWAT, eWAT, and liver from mice treated as (A). Scale bar, 200 μm.

(G-I) Lipid droplet size distributions in BAT (G), iWAT (H), and eWAT (I) were quantified from the images (F). n = 3000 droplets per group in BAT and n = 500 droplets per group in iWAT and eWAT. Pooled individual droplets are shown on the top, and the mean lipid droplet size per mouse is shown on the bottom (n = 4 per group).

(J, K) mRNA expression analysis in eWAT (J) (n = 6 per group) and liver (K) (n = 6 per group) from mice treated as in (A).

All experiments were independently repeated two times with consistent results. Data are presented as mean ± SEM of biologically independent samples. Statistical significance was determined by one-way ANOVA (D-E, G-K). **p* < 0.05, ***p* < 0.01, ****p* < 0.001.

**
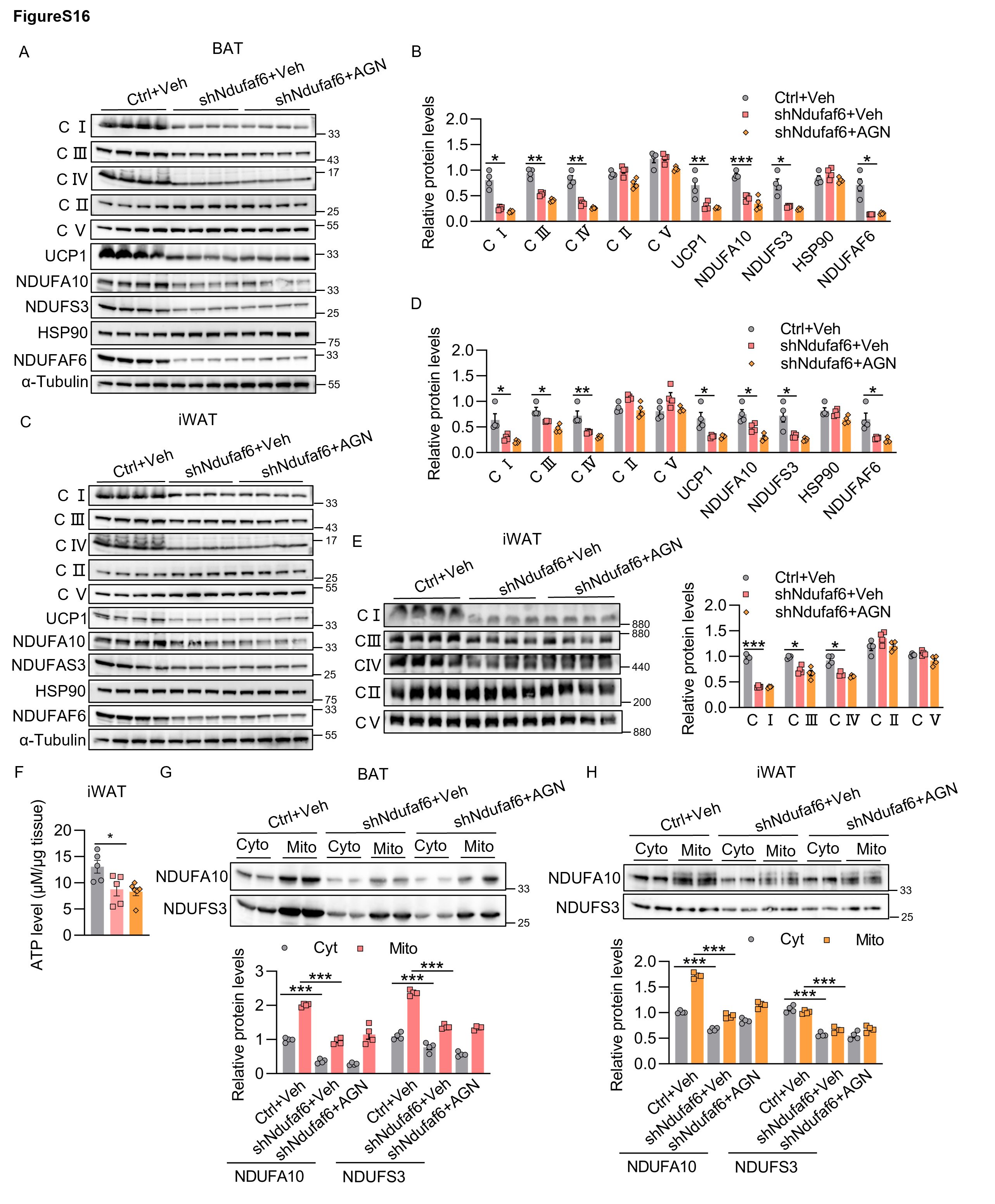
**

**Figure S16. NDUFAF6 is required for agnuside-induced respiratory complex assembly and ATP production.**

(A-D) Western blot analysis of representative protein expression in BAT (A) and iWAT (C) from *Ndufaf6* knockdown mice following 12 weeks of AGN treatment, with densitometric quantification shown in (B) and (D) (n = 4 per group).

(E) BN-PAGE analysis of mitochondria isolated from iWAT of mice treated as in (A), with quantification shown on the right (n = 4 per group).

(F) ATP levels in iWAT from mice treated as in (A) (n = 5-6 per group).

(G-H) Western blot analysis of NDUFA10 and NDUFS3 protein expression in cytosolic and mitochondrial fractions of BAT (G) and iWAT (H) from sh*Ndufaf6* mice treated as in (A) (n = 2 per group). Note (G, H): The cytosolic fraction was obtained as the 10,000 g supernatant without additional ultracentrifugation, and may therefore include minor microsomal contamination

All experiments were independently repeated two times with consistent results. Data are presented as mean ± SEM of biologically independent samples. Statistical significance was determined by one-way ANOVA (B, D, E-H). **p* < 0.05, ***p* < 0.01, ****p* < 0.001.
