# Supplementary material for: Agnuside Stabilizes the Complex I Assembly Factor NDUFAF6 to Reinforce Mitochondrial Efficiency and Thermogenic Responsiveness
Source: Adv Sci (Weinh). 2026 Jun 16:e16501. Online ahead of print. doi: 10.1002/advs.202516501 (PMC13337116; doi:10.1002/advs.202516501)
Supplement: Supplementary file 2 — Supporting File 2: advs76095‐sup‐0002‐TableS1‐S4.pptx. [file ADVS-9999-e16501-s002.pptx]

## Slide 1
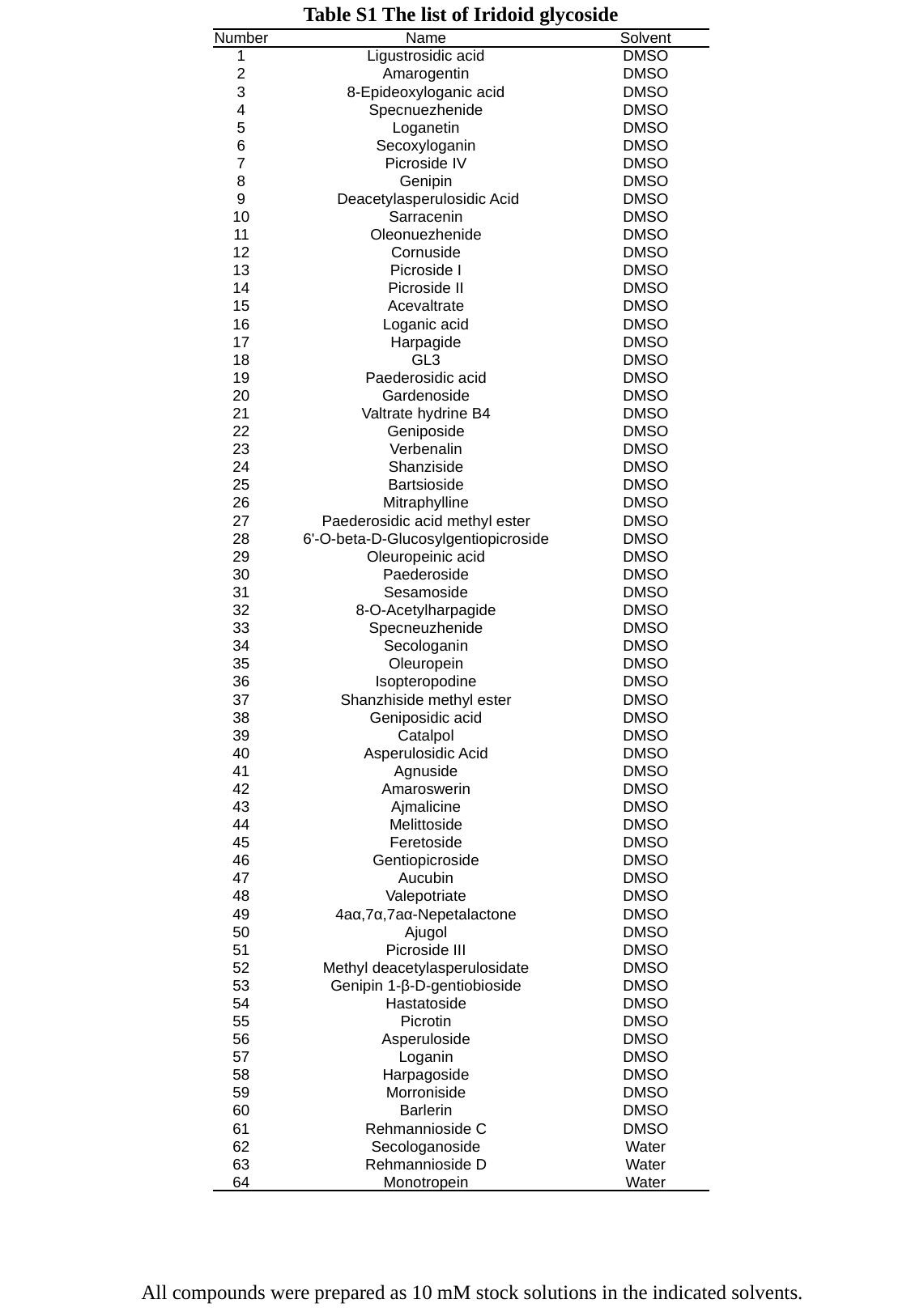

Table S1 The list of Iridoid glycoside
| Number | Name | Solvent |
| --- | --- | --- |
| 1 | Ligustrosidic acid | DMSO |
| 2 | Amarogentin | DMSO |
| 3 | 8-Epideoxyloganic acid | DMSO |
| 4 | Specnuezhenide | DMSO |
| 5 | Loganetin | DMSO |
| 6 | Secoxyloganin | DMSO |
| 7 | Picroside IV | DMSO |
| 8 | Genipin | DMSO |
| 9 | Deacetylasperulosidic Acid | DMSO |
| 10 | Sarracenin | DMSO |
| 11 | Oleonuezhenide | DMSO |
| 12 | Cornuside | DMSO |
| 13 | Picroside I | DMSO |
| 14 | Picroside II | DMSO |
| 15 | Acevaltrate | DMSO |
| 16 | Loganic acid | DMSO |
| 17 | Harpagide | DMSO |
| 18 | GL3 | DMSO |
| 19 | Paederosidic acid | DMSO |
| 20 | Gardenoside | DMSO |
| 21 | Valtrate hydrine B4 | DMSO |
| 22 | Geniposide | DMSO |
| 23 | Verbenalin | DMSO |
| 24 | Shanziside | DMSO |
| 25 | Bartsioside | DMSO |
| 26 | Mitraphylline | DMSO |
| 27 | Paederosidic acid methyl ester | DMSO |
| 28 | 6'-O-beta-D-Glucosylgentiopicroside | DMSO |
| 29 | Oleuropeinic acid | DMSO |
| 30 | Paederoside | DMSO |
| 31 | Sesamoside | DMSO |
| 32 | 8-​O-​Acetylharpagide | DMSO |
| 33 | Specneuzhenide | DMSO |
| 34 | Secologanin | DMSO |
| 35 | Oleuropein | DMSO |
| 36 | Isopteropodine | DMSO |
| 37 | Shanzhiside methyl ester | DMSO |
| 38 | Geniposidic acid | DMSO |
| 39 | Catalpol | DMSO |
| 40 | Asperulosidic Acid | DMSO |
| 41 | Agnuside | DMSO |
| 42 | Amaroswerin | DMSO |
| 43 | Ajmalicine | DMSO |
| 44 | Melittoside | DMSO |
| 45 | Feretoside | DMSO |
| 46 | Gentiopicroside | DMSO |
| 47 | Aucubin | DMSO |
| 48 | Valepotriate | DMSO |
| 49 | 4aα,7α,7aα-Nepetalactone | DMSO |
| 50 | Ajugol | DMSO |
| 51 | Picroside III | DMSO |
| 52 | Methyl deacetylasperulosidate | DMSO |
| 53 | Genipin 1-β-D-gentiobioside | DMSO |
| 54 | Hastatoside | DMSO |
| 55 | Picrotin | DMSO |
| 56 | Asperuloside | DMSO |
| 57 | Loganin | DMSO |
| 58 | Harpagoside | DMSO |
| 59 | Morroniside | DMSO |
| 60 | Barlerin | DMSO |
| 61 | Rehmannioside C | DMSO |
| 62 | Secologanoside | Water |
| 63 | Rehmannioside D | Water |
| 64 | Monotropein | Water |
All compounds were prepared as 10 mM stock solutions in the indicated solvents.

## Slide 2
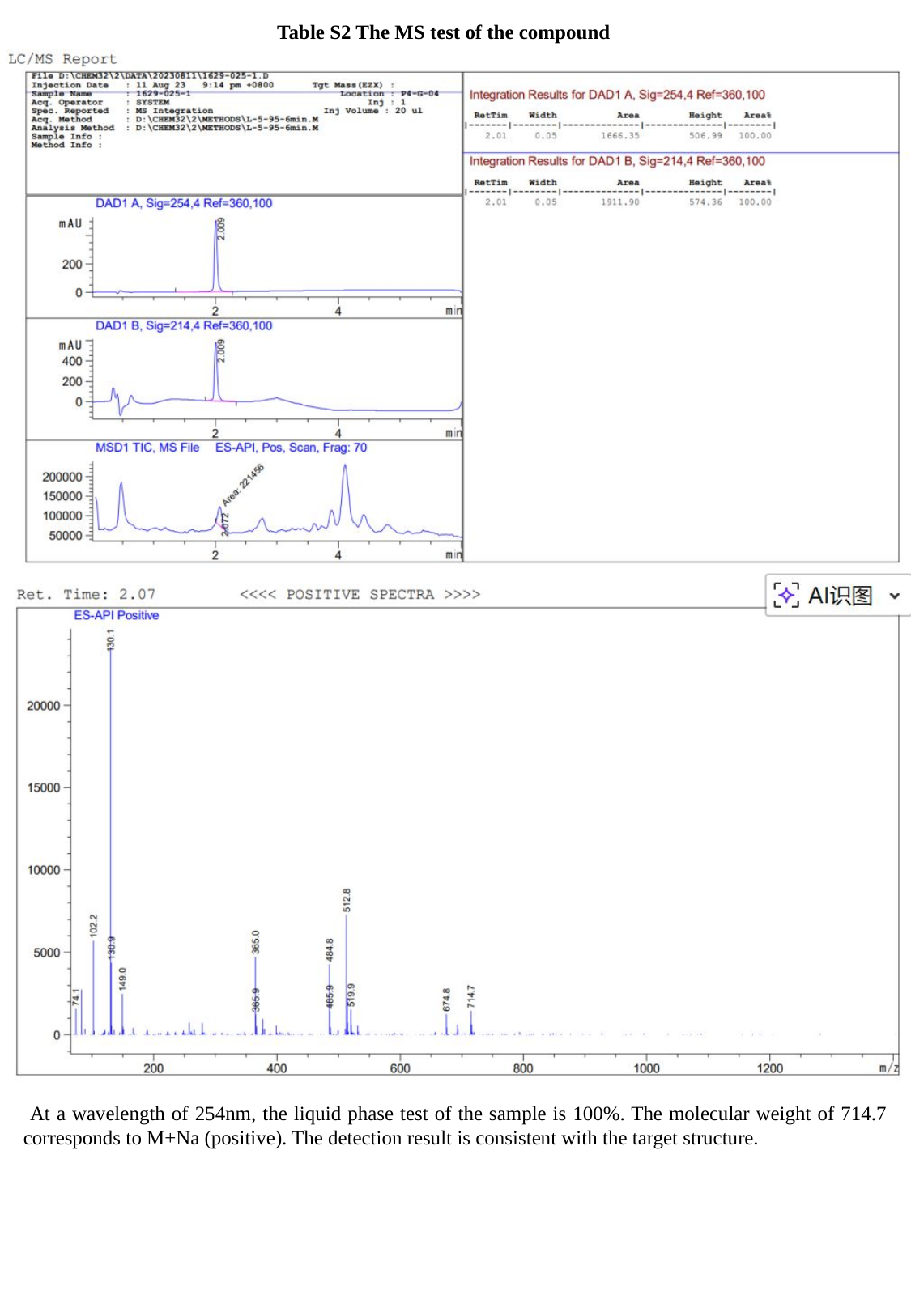

Table S2 The MS test of the compound
 At a wavelength of 254nm, the liquid phase test of the sample is 100%. The molecular weight of 714.7 corresponds to M+Na (positive). The detection result is consistent with the target structure.

## Slide 3
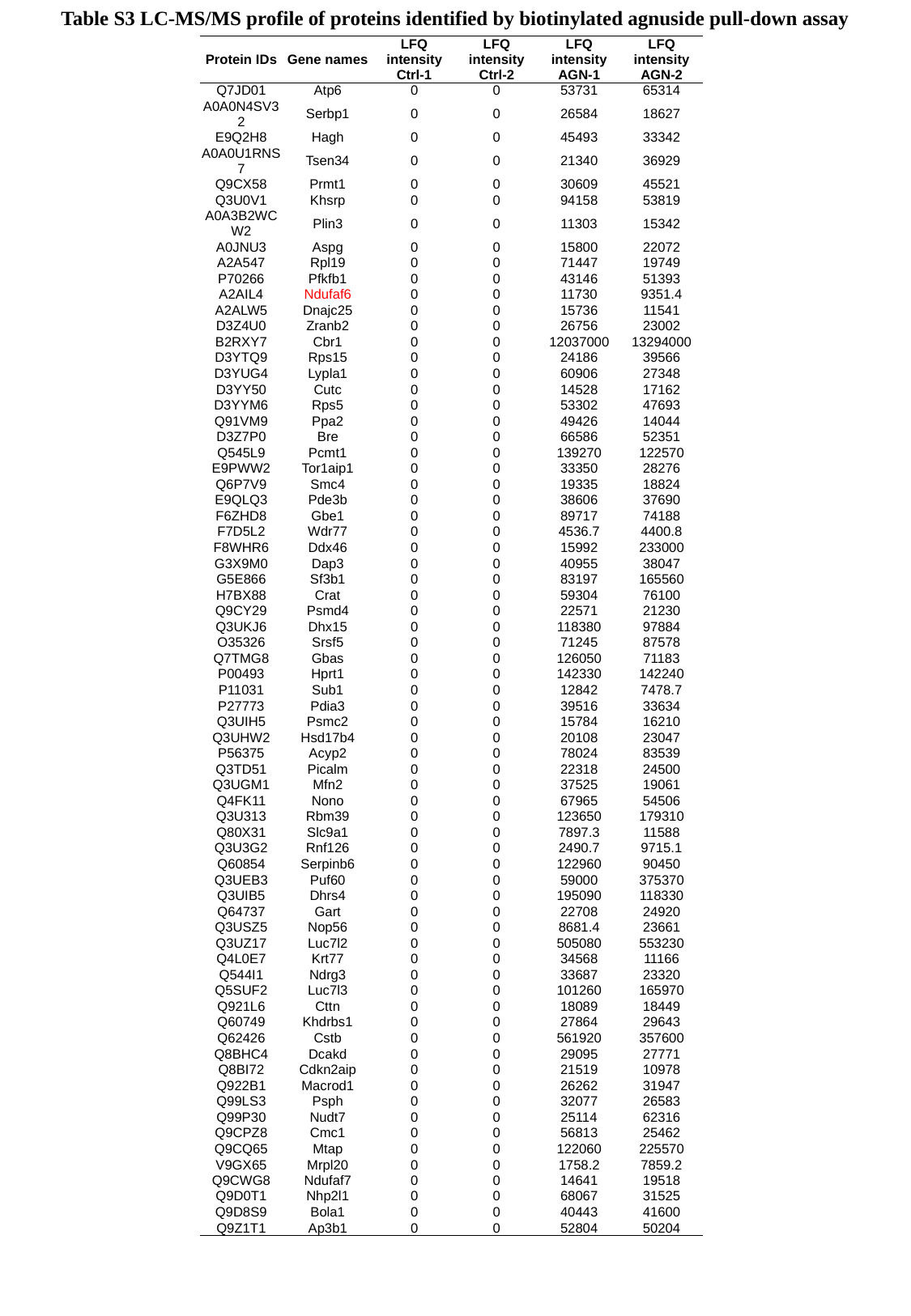

Table S3 LC-MS/MS profile of proteins identified by biotinylated agnuside pull-down assay
| Protein IDs | Gene names | LFQ intensity Ctrl-1 | LFQ intensity Ctrl-2 | LFQ intensity AGN-1 | LFQ intensity AGN-2 |
| --- | --- | --- | --- | --- | --- |
| Q7JD01 | Atp6 | 0 | 0 | 53731 | 65314 |
| A0A0N4SV32 | Serbp1 | 0 | 0 | 26584 | 18627 |
| E9Q2H8 | Hagh | 0 | 0 | 45493 | 33342 |
| A0A0U1RNS7 | Tsen34 | 0 | 0 | 21340 | 36929 |
| Q9CX58 | Prmt1 | 0 | 0 | 30609 | 45521 |
| Q3U0V1 | Khsrp | 0 | 0 | 94158 | 53819 |
| A0A3B2WCW2 | Plin3 | 0 | 0 | 11303 | 15342 |
| A0JNU3 | Aspg | 0 | 0 | 15800 | 22072 |
| A2A547 | Rpl19 | 0 | 0 | 71447 | 19749 |
| P70266 | Pfkfb1 | 0 | 0 | 43146 | 51393 |
| A2AIL4 | Ndufaf6 | 0 | 0 | 11730 | 9351.4 |
| A2ALW5 | Dnajc25 | 0 | 0 | 15736 | 11541 |
| D3Z4U0 | Zranb2 | 0 | 0 | 26756 | 23002 |
| B2RXY7 | Cbr1 | 0 | 0 | 12037000 | 13294000 |
| D3YTQ9 | Rps15 | 0 | 0 | 24186 | 39566 |
| D3YUG4 | Lypla1 | 0 | 0 | 60906 | 27348 |
| D3YY50 | Cutc | 0 | 0 | 14528 | 17162 |
| D3YYM6 | Rps5 | 0 | 0 | 53302 | 47693 |
| Q91VM9 | Ppa2 | 0 | 0 | 49426 | 14044 |
| D3Z7P0 | Bre | 0 | 0 | 66586 | 52351 |
| Q545L9 | Pcmt1 | 0 | 0 | 139270 | 122570 |
| E9PWW2 | Tor1aip1 | 0 | 0 | 33350 | 28276 |
| Q6P7V9 | Smc4 | 0 | 0 | 19335 | 18824 |
| E9QLQ3 | Pde3b | 0 | 0 | 38606 | 37690 |
| F6ZHD8 | Gbe1 | 0 | 0 | 89717 | 74188 |
| F7D5L2 | Wdr77 | 0 | 0 | 4536.7 | 4400.8 |
| F8WHR6 | Ddx46 | 0 | 0 | 15992 | 233000 |
| G3X9M0 | Dap3 | 0 | 0 | 40955 | 38047 |
| G5E866 | Sf3b1 | 0 | 0 | 83197 | 165560 |
| H7BX88 | Crat | 0 | 0 | 59304 | 76100 |
| Q9CY29 | Psmd4 | 0 | 0 | 22571 | 21230 |
| Q3UKJ6 | Dhx15 | 0 | 0 | 118380 | 97884 |
| O35326 | Srsf5 | 0 | 0 | 71245 | 87578 |
| Q7TMG8 | Gbas | 0 | 0 | 126050 | 71183 |
| P00493 | Hprt1 | 0 | 0 | 142330 | 142240 |
| P11031 | Sub1 | 0 | 0 | 12842 | 7478.7 |
| P27773 | Pdia3 | 0 | 0 | 39516 | 33634 |
| Q3UIH5 | Psmc2 | 0 | 0 | 15784 | 16210 |
| Q3UHW2 | Hsd17b4 | 0 | 0 | 20108 | 23047 |
| P56375 | Acyp2 | 0 | 0 | 78024 | 83539 |
| Q3TD51 | Picalm | 0 | 0 | 22318 | 24500 |
| Q3UGM1 | Mfn2 | 0 | 0 | 37525 | 19061 |
| Q4FK11 | Nono | 0 | 0 | 67965 | 54506 |
| Q3U313 | Rbm39 | 0 | 0 | 123650 | 179310 |
| Q80X31 | Slc9a1 | 0 | 0 | 7897.3 | 11588 |
| Q3U3G2 | Rnf126 | 0 | 0 | 2490.7 | 9715.1 |
| Q60854 | Serpinb6 | 0 | 0 | 122960 | 90450 |
| Q3UEB3 | Puf60 | 0 | 0 | 59000 | 375370 |
| Q3UIB5 | Dhrs4 | 0 | 0 | 195090 | 118330 |
| Q64737 | Gart | 0 | 0 | 22708 | 24920 |
| Q3USZ5 | Nop56 | 0 | 0 | 8681.4 | 23661 |
| Q3UZ17 | Luc7l2 | 0 | 0 | 505080 | 553230 |
| Q4L0E7 | Krt77 | 0 | 0 | 34568 | 11166 |
| Q544I1 | Ndrg3 | 0 | 0 | 33687 | 23320 |
| Q5SUF2 | Luc7l3 | 0 | 0 | 101260 | 165970 |
| Q921L6 | Cttn | 0 | 0 | 18089 | 18449 |
| Q60749 | Khdrbs1 | 0 | 0 | 27864 | 29643 |
| Q62426 | Cstb | 0 | 0 | 561920 | 357600 |
| Q8BHC4 | Dcakd | 0 | 0 | 29095 | 27771 |
| Q8BI72 | Cdkn2aip | 0 | 0 | 21519 | 10978 |
| Q922B1 | Macrod1 | 0 | 0 | 26262 | 31947 |
| Q99LS3 | Psph | 0 | 0 | 32077 | 26583 |
| Q99P30 | Nudt7 | 0 | 0 | 25114 | 62316 |
| Q9CPZ8 | Cmc1 | 0 | 0 | 56813 | 25462 |
| Q9CQ65 | Mtap | 0 | 0 | 122060 | 225570 |
| V9GX65 | Mrpl20 | 0 | 0 | 1758.2 | 7859.2 |
| Q9CWG8 | Ndufaf7 | 0 | 0 | 14641 | 19518 |
| Q9D0T1 | Nhp2l1 | 0 | 0 | 68067 | 31525 |
| Q9D8S9 | Bola1 | 0 | 0 | 40443 | 41600 |
| Q9Z1T1 | Ap3b1 | 0 | 0 | 52804 | 50204 |

## Slide 4
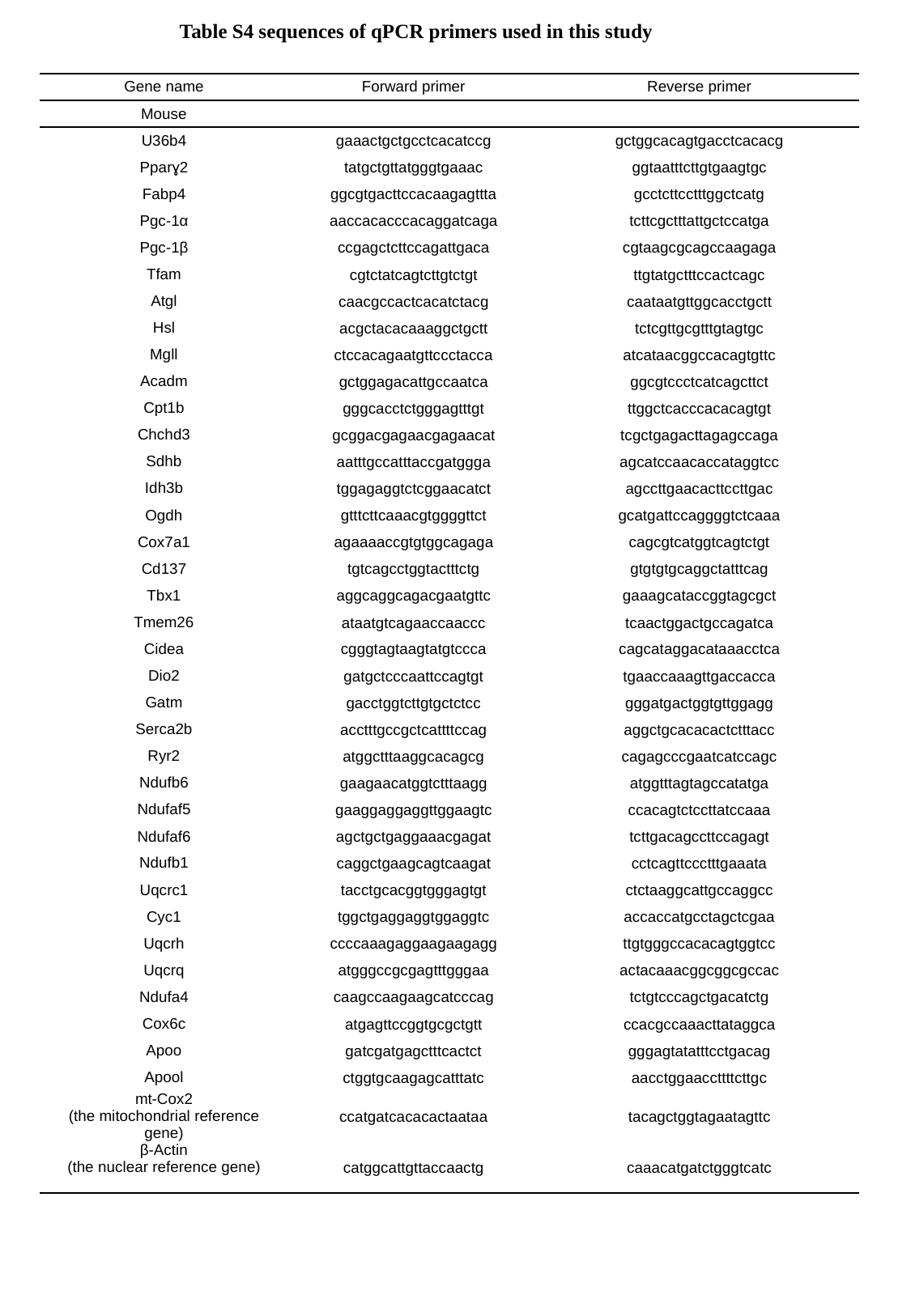

Table S4 sequences of qPCR primers used in this study
| Gene name | Forward primer | Reverse primer |
| --- | --- | --- |
| Mouse | | |
| U36b4 | gaaactgctgcctcacatccg | gctggcacagtgacctcacacg |
| Pparɣ2 | tatgctgttatgggtgaaac | ggtaatttcttgtgaagtgc |
| Fabp4 | ggcgtgacttccacaagagttta | gcctcttcctttggctcatg |
| Pgc-1α | aaccacacccacaggatcaga | tcttcgctttattgctccatga |
| Pgc-1β | ccgagctcttccagattgaca | cgtaagcgcagccaagaga |
| Tfam | cgtctatcagtcttgtctgt | ttgtatgctttccactcagc |
| Atgl | caacgccactcacatctacg | caataatgttggcacctgctt |
| Hsl | acgctacacaaaggctgctt | tctcgttgcgtttgtagtgc |
| Mgll | ctccacagaatgttccctacca | atcataacggccacagtgttc |
| Acadm | gctggagacattgccaatca | ggcgtccctcatcagcttct |
| Cpt1b | gggcacctctgggagtttgt | ttggctcacccacacagtgt |
| Chchd3 | gcggacgagaacgagaacat | tcgctgagacttagagccaga |
| Sdhb | aatttgccatttaccgatggga | agcatccaacaccataggtcc |
| Idh3b | tggagaggtctcggaacatct | agccttgaacacttccttgac |
| Ogdh | gtttcttcaaacgtggggttct | gcatgattccaggggtctcaaa |
| Cox7a1 | agaaaaccgtgtggcagaga | cagcgtcatggtcagtctgt |
| Cd137 | tgtcagcctggtactttctg | gtgtgtgcaggctatttcag |
| Tbx1 | aggcaggcagacgaatgttc | gaaagcataccggtagcgct |
| Tmem26 | ataatgtcagaaccaaccc | tcaactggactgccagatca |
| Cidea | cgggtagtaagtatgtccca | cagcataggacataaacctca |
| Dio2 | gatgctcccaattccagtgt | tgaaccaaagttgaccacca |
| Gatm | gacctggtcttgtgctctcc | gggatgactggtgttggagg |
| Serca2b | acctttgccgctcattttccag | aggctgcacacactctttacc |
| Ryr2 | atggctttaaggcacagcg | cagagcccgaatcatccagc |
| Ndufb6 | gaagaacatggtctttaagg | atggtttagtagccatatga |
| Ndufaf5 | gaaggaggaggttggaagtc | ccacagtctccttatccaaa |
| Ndufaf6 | agctgctgaggaaacgagat | tcttgacagccttccagagt |
| Ndufb1 | caggctgaagcagtcaagat | cctcagttccctttgaaata |
| Uqcrc1 | tacctgcacggtgggagtgt | ctctaaggcattgccaggcc |
| Cyc1 | tggctgaggaggtggaggtc | accaccatgcctagctcgaa |
| Uqcrh | ccccaaagaggaagaagagg | ttgtgggccacacagtggtcc |
| Uqcrq | atgggccgcgagtttgggaa | actacaaacggcggcgccac |
| Ndufa4 | caagccaagaagcatcccag | tctgtcccagctgacatctg |
| Cox6c | atgagttccggtgcgctgtt | ccacgccaaacttataggca |
| Apoo | gatcgatgagctttcactct | gggagtatatttcctgacag |
| Apool | ctggtgcaagagcatttatc | aacctggaaccttttcttgc |
| mt-Cox2 (the mitochondrial reference gene) | ccatgatcacacactaataa | tacagctggtagaatagttc |
| β-Actin (the nuclear reference gene) | catggcattgttaccaactg | caaacatgatctgggtcatc |
